# H3K27ac acetylome signatures reveal the epigenomic reorganization in remodeled non-failing human hearts

**DOI:** 10.1186/s13148-020-00895-5

**Published:** 2020-07-14

**Authors:** Jiayi Pei, Magdalena Harakalova, Thomas A. Treibel, R Thomas Lumbers, Bastiaan J. Boukens, Igor R. Efimov, Jip T. van Dinter, Arantxa González, Begoña López, Hamid El Azzouzi, Noortje van den Dungen, Christian G. M. van Dijk, Merle M. Krebber, Hester M. den Ruijter, Gerard Pasterkamp, Dirk J. Duncker, Edward E. S. Nieuwenhuis, Roel de Weger, Manon M. Huibers, Aryan Vink, Jason H. Moore, James C. Moon, Marianne C. Verhaar, Georgios Kararigas, Michal Mokry, Folkert W. Asselbergs, Caroline Cheng

**Affiliations:** 1 Department of Nephrology and Hypertension, DIGD, UMC Utrecht, University of Utrecht, Utrecht, Netherlands; 2 Department of Cardiology, Division Heart & Lungs, UMC Utrecht, University of Utrecht, Utrecht, Netherlands; 3 Regenerative Medicine Utrecht (RMU), UMC Utrecht, University of Utrecht, Utrecht, Netherlands; 4 Department of Pathology, UMC Utrecht, University of Utrecht, Utrecht, Netherlands; 5 Institute of Cardiovascular Science, University College London, London, UK; 6 Department of Medical Biology, AMC, Amsterdam, Netherlands; 7 Department of Biomedical Engineering, GWU, Washington, D.C, USA; 8 Program of Cardiovascular Diseases, CIMA Universidad de Navarra and IdiSNA, Pamplona, Spain; 9 CIBERCV, Carlos III Institute of Health, Madrid, Spain; 10 Laboratory of Clinical Chemistry and Hematology, UMC Utrecht, Utrecht, Netherlands; 11 Department of Experimental Cardiology, UMC Utrecht, University of Utrecht, Utrecht, Netherlands; 12 Division of Experimental Cardiology, Department of Cardiology, Erasmus University Medical Center, Rotterdam, Netherlands; 13 Division of Paediatrics, UMC Utrecht, University of Utrecht, Utrecht, Netherlands; 14 Institute for Biomedical Informatics, UPENN, Philadelphia, USA; 15 Charité – Universitätsmedizin Berlin, and DZHK (German Centre for Cardiovascular Research), partner site, Berlin, Germany; 16 Institute of Cardiovascular Science, Faculty of Population Health Science, University College London, London, UK; 17 Health Data Research UK and Institute of Health Informatics, University College London, London, UK

**Keywords:** Myocardial remodeling, Histone acetylation, Transcriptome, Transcription factor

## Abstract

**Background:**

H3K27ac histone acetylome changes contribute to the phenotypic response in heart diseases, particularly in end-stage heart failure. However, such epigenetic alterations have not been systematically investigated in remodeled non-failing human hearts. Therefore, valuable insight into cardiac dysfunction in early remodeling is lacking. This study aimed to reveal the acetylation changes of chromatin regions in response to myocardial remodeling and their correlations to transcriptional changes of neighboring genes.

**Results:**

We detected chromatin regions with differential acetylation activity (DARs; *P*_adj._ < 0.05) between remodeled non-failing patient hearts and healthy donor hearts. The acetylation level of the chromatin region correlated with its RNA polymerase II occupancy level and the mRNA expression level of its adjacent gene per sample. Annotated genes from DARs were enriched in disease-related pathways, including fibrosis and cell metabolism regulation. DARs that change in the same direction have a tendency to cluster together, suggesting the well-reorganized chromatin architecture that facilitates the interactions of regulatory domains in response to myocardial remodeling. We further show the differences between the acetylation level and the mRNA expression level of cell-type-specific markers for cardiomyocytes and 11 non-myocyte cell types. Notably, we identified transcriptome factor (TF) binding motifs that were enriched in DARs and defined TFs that were predicted to bind to these motifs. We further showed 64 genes coding for these TFs that were differentially expressed in remodeled myocardium when compared with controls.

**Conclusions:**

Our study reveals extensive novel insight on myocardial remodeling at the DNA regulatory level. Differences between the acetylation level and the transcriptional level of cell-type-specific markers suggest additional mechanism(s) between acetylome and transcriptome. By integrating these two layers of epigenetic profiles, we further provide promising TF-encoding genes that could serve as master regulators of myocardial remodeling. Combined, our findings highlight the important role of chromatin regulatory signatures in understanding disease etiology.

## Introduction

Myocardial remodeling is defined as changes in the size, shape, structure, and function of the heart from cardiac injury due to various causes [1, 2]. It is a complex process resulting from the interplay between cardiomyocytes (hypertrophy), cardiac fibroblasts (fibrosis), vascular smooth muscle cells (vascular stiffening), vascular endothelial cells (endothelial dysfunction), and leukocytes (inflammation) [2]. Pathological myocardial remodeling has a poor prognosis related to a higher risk of heart failure and sudden cardiac death [3]. Given the scarce availability of patient and control cardiac biopsies in humans, most mechanistic studies on myocardial remodeling are based on animal models or cultured cells [4–6] that do not necessarily represent the patient’s situation completely [7–9]. Human myocardial biopsies have been used to investigate remodeling between health and disease on the level of the methylome, transcriptome, and proteome [10–14]. However, chromatin regulation, defined as the dynamic modification of chromatin architecture to control gene expression [15, 16], has not been systematically investigated. However, a previous study mapped the epigenome in failing human hearts [17]. Although the epigenetic regulation of specific classes of genes has been suggested to contribute to the phenotypic response throughout the mild-stage to the end-stage heart failure [18], there is still a lack of insight into the early stage of cardiac dysfunction, which could be elucidated by studying chromatin regulation changes between healthy and remodeled non-failing human hearts.

Chromatin immunoprecipitation and sequencing (ChIP-seq) is widely utilized to study chromatin regulation [19]. Key DNA regulatory regions and pathways in several (mostly inflammatory) diseases have been identified using ChIP-seq [20, 21]. Histone 3 lysine 27 acetylation (H3K27ac) is found at both active enhancers and promoters, which are accessible to transcription factors (TFs), polymerases, and other members of the transcriptional complex in order to regulate transcription of genes [22]. Histone acetylome studies using the H3K27ac mark have successfully identified chromatin regulation changes under healthy and diseased conditions [23, 24]. Additionally, the H3K27ac level correlates with gene expression levels [16]. Other histone marks that are indicative of transcription include promoter-regions-associated H3K4me3 and H3K9ac and gene-bodies-associated H3K36me3 and H3K79me3 [25]. A recent paper further indicates that the H3K27ac and H3K36me3 marks serve as the best predictive marks for pathological gene programming in diseased human cardiomyocytes when compared with other histone marks [17]. Additionally, studies have shown that H3K27ac exhibits the best correlation with both active promoters and enhancers when compared with other marks [26, 27]. Despite the rapid increase of ChIP-seq data over the last decade [28, 29], the H3K27ac landscape that defines myocardial remodeling has been only scarcely investigated [17], mainly due to the lack of proper samples and controls.

To improve our understanding of myocardial remodeling at the DNA regulatory level, in this study, we employ a ChIP-seq approach to characterize the H3K27ac binding landscape. Our findings provide new insights into the early disease etiology, revealing genome-wide histone acetylation changes between rare remodeled non-failing myocardial biopsies from patients with severe aortic stenosis (AS) [30] and control samples from non-transplanted donor hearts. Firstly, our study yields a unique list of differentially acetylated regions (DARs) between these two groups. Secondly, by studying the chromatin dynamics, we show the well-organized chromatin structure that physically facilitates gene regulation. Enrichment analysis using annotated genes from DARs indicate altered extracellular matrix (ECM) organization and cell metabolism regulation in remodeled myocardium. Thirdly, we investigate the correlation between histone acetylation changes and the transcriptome changes using data from our study and published studies. Fourthly, we present the differences between the acetylation levels and the mRNA expression level of markers specific for cardiomyocytes and 11 non-myocyte cell types in response to myocardial remodeling, suggesting the additional mechanism(s) between chromatin acetylation and gene transcription. Lastly, we have identified TF binding motifs that are enriched in DARs and genes coding for TFs predicted to bind to these enriched motifs. Notably, 64 TF-encoding genes are differentially expressed in remodeled myocardium versus controls, and they may serve as candidate master regulators of myocardial remodeling.

## Results

### DARs between non-failing remodeled hearts and control hearts

**Fig. 1:**
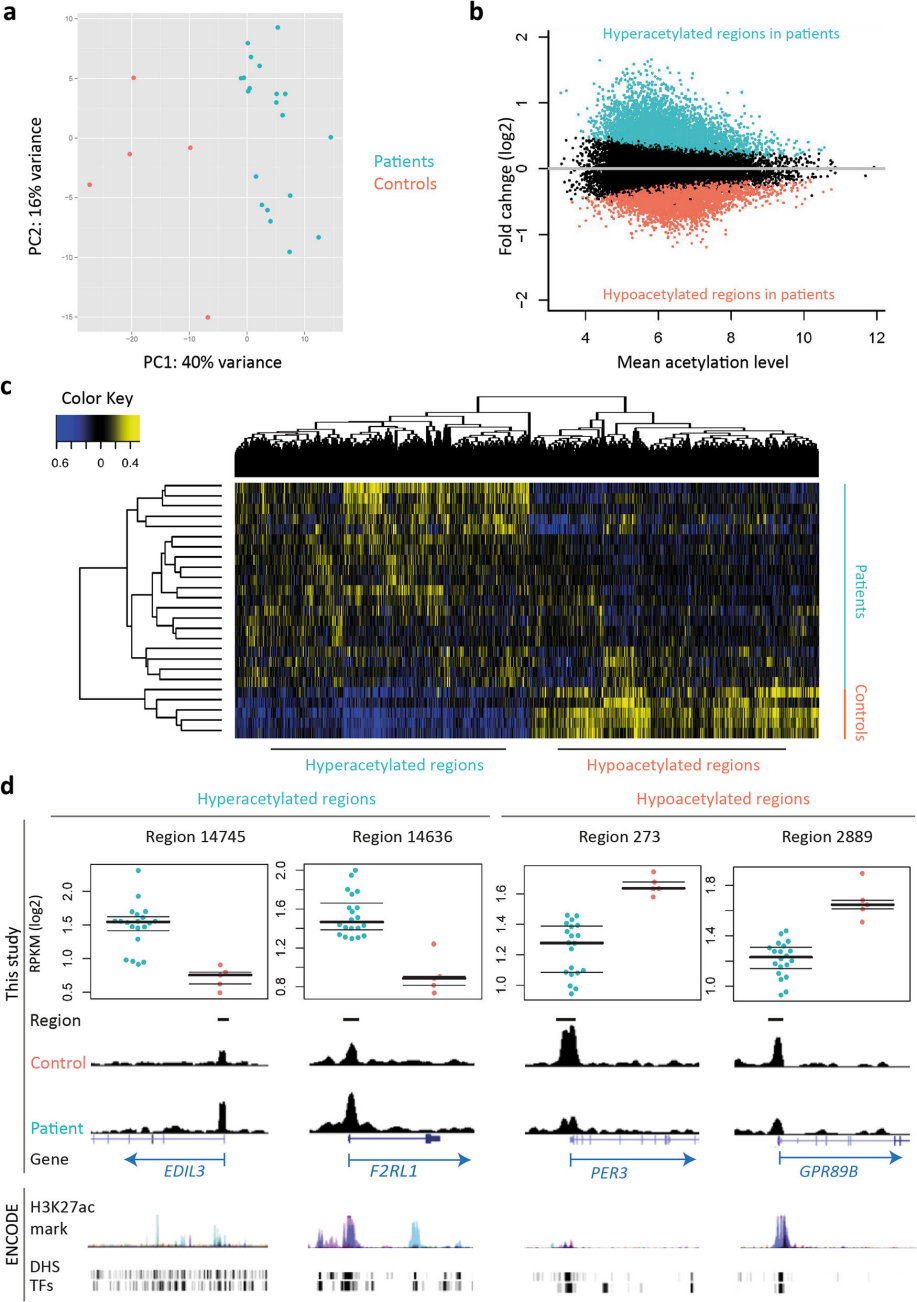
Differentially acetylated H3K27ac regions between patients and controls. **a** Principal component analysis (PCA) plot showing the clustering of patient and control samples based on H3K27ac profiles (using 500 regions with the highest variance). **b** MA plot showing the mean acetylation levels of all samples (*x*-axis) and the fold changes between two groups in the log_2_ scale (*y*-axis). Colored dots represent hyper (aqua)- and hypoacetylated (coral) regions in patients compared with controls respectively (adjusted *p* value < 0.05). **c** Heatmap depicts the clustering of samples based on all DARs acetylation levels. **d** Examples of DARs in the UCSC genome browser. Dot plots depict the acetylation level in patients (blue) and controls (orange). ENCODE = public ENCODE data default display. H3K27ac mark = ChIP-seq data of 7 cell types obtained from ENCODE. DHS = DNaseI hypersensitivity clusters in 125 cells. TFs = ChIP-seq data of 161 transcription factors

We studied histone acetylation activity using H3K27ac ChIP-seq in myocardium from AS patient hearts when compared with control hearts, followed by multiple in silico analyses to illustrate chromatin structural dynamics and biological functions enriched by annotated genes in remodeled myocardium (Supplementary Figure 1). On average, we obtained 40,745 ± 9656 and 16,734 ± 6896 regions using H3K27ac ChIP-seq in control and patient samples, respectively. Among detected H3K27ac regions, 27,879 regions were supported by at least two independent samples, out of which 11,358 unique regions showed significantly different H3K27ac occupancy between two groups (adjusted *p* value < 0.05, Fig. 1a, b, and c, Supplementary Table 1). From these, we identified 5634 regions with increased signal in patients (hyperacetylation) and 5724 regions with increased signal in controls (hypoacetylation). Examples of hyper- and hypoacetylated regions are shown in Fig. 1d. This altogether demonstrates extensive changes in histone acetylation upon myocardial remodeling.

### Adjacent DARs tend to have the same direction in their acetylation changes

**Fig. 2:**
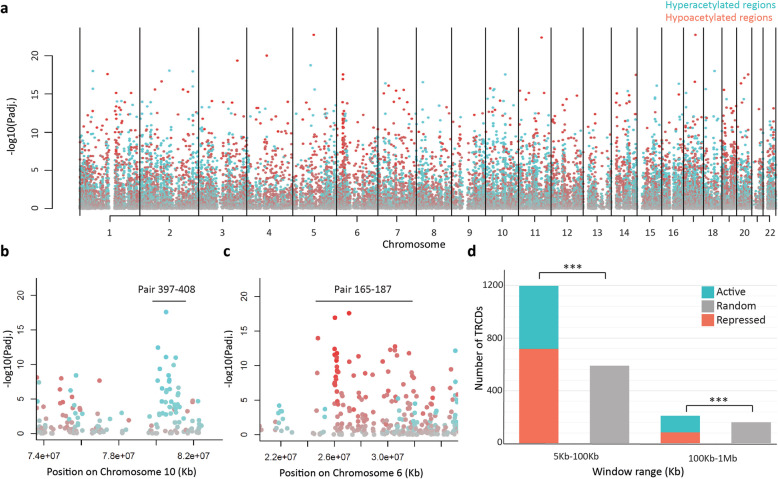
Distribution of tandem regulated chromatin domains (TRCDs). **a** Manhattan plot depicting the distribution of differentially H3K27 acetylated regions in patients vs. controls: non-significant regions (grey), hyperacetylated regions (aqua), and hypoacetylated regions (coral). **b** Zoomed-in view of clusters of TRCDs in the short range (indicated with the bar with higher acetylation level in patients than in controls on chromosome 10). **c** Zoomed-in view of clusters of TRCDs in the short range (indicated with the bar) with lower acetylation level in patients than in controls on chromosome 6. **d** Number of identified active and repressed TRCDs in short- and long-genomic distance respectively in real and randomly distributed dataset

A growing number of studies using chromatin conformation capture techniques (i.e., 4C-seq and Hi-C) have shown that compartments and topologically associating domains (TADs) are tissue-specific and contain preferential interactions of certain regulatory elements and genes [31]. Changes within and among TADs influence gene regulation, and such changes have been observed in cancer [31]. We found that regions with the same direction in acetylation change upon myocardial remodeling are more likely located next to each other (Fig. 2a, b, and c). Using the adjacent (5–100 kb) peak pairs, we have found that one DAR is more likely followed by regions with the same direction of acetylation change when compared with randomized regions (*p* value = 2.4e−108, Fig. 2d). Similar behavior was also observed in more distal (100 kb–1 Mb) peak pairs (*p* value = 0.00012, Fig. 2d, and Supplementary Table 2). This suggests that adjacent DARs with the same direction in acetylation change might function together as larger regulatory domains or potential subdomains within TADs.

### Annotated genes from DARs are well correlated with their transcriptome changes

We performed region-to-gene annotation from DARs. Briefly, we mapped genes located in the vicinity of DARs using a conservative window of ± 5 kb from the transcription start site (TSS), which is recognized to be within the promoter region range of most genes. In total, we annotated 1294 and 5886 genes in the hyper- and hypoacetylated regions, respectively (Supplementary Table 3). Examples of noteworthy annotated genes in the hyper- and hypoacetylated regions are shown in Fig. 1d.

**Fig. 3:**
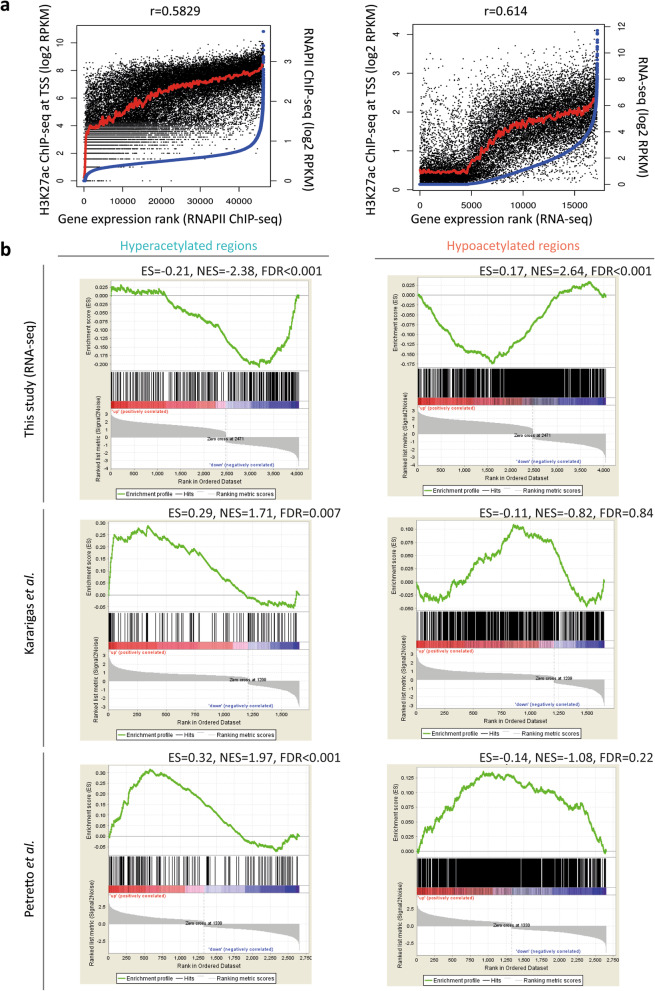
Correlation analysis of H3K27ac ChIP-seq data, RNAPII ChIP-seq data, and RNA-seq data. **a** Correlation between H3K27ac ChIP-seq vs. RNAPII ChIP-seq data (left plot) and RNA-seq data (right plot) in the same sample, respectively. H3K27ac ChIP-seq data is presented on the y-axis, whereas RNAPII ChIP-seq data and RNA-seq data are shown on the *x*-axis and *z*-axis (log_2_ scale). **b** Correlation between annotated genes from differentially acetylated regions and differentially expressed genes from transcriptome profiles from our study and two published studies. Differentially expressed genes per study are ranked by their fold changes and shown on the *x*-axis. The running correlation throughout the gene set is shown by the curve (green) and the running enrichment score (ES) is shown in the *y*-axis. Enrichment score normalized for gene set size (NES) and the false discovery rate (FDR) are shown above each plot. Black bars indicate annotated genes from differentially acetylated regions that are presented among the transcriptome profiles

First, we confirmed that the H3K27ac signal corresponded to gene expression levels as proxied by RNA polymerase II (RNAPII) occupancy and RNA sequencing (RNA-seq) per sample (Fig. 3a and Supplementary Figure 3). Next, we employed gene set enrichment analysis to examine the correlation of annotated genes from DARs with their mRNA expression changes in the transcription profiles obtained in this study and in previously published studies [11, 12]. Based on 4240 differentially expressed genes between patients and controls from the RNA-seq dataset in our study (*p* value < 0.05, Supplementary Figure 4 and Supplementary Table 4), we showed a significant correlation between annotated genes from the hypoacetylated regions and genes with lower expression levels in patients (FDR < 0.001, Fig. 3b). In contrast, annotated genes from the hyperacetylated regions were not enriched in the pool of genes with higher expression levels in patients when compared with controls. However, annotated genes from the hyperacetylated regions showed a statistically significant correlation with genes with higher expression levels in patients when compared with controls from two published studies (FDR = 0.007 and FDR < 0.001). There was no significant correlation between the annotated genes from the hypoacetylated regions in the pool of lower expressed genes in these two studies (FDR = 0.84 and FDR = 0.22, Fig. 3b). Taken together, these data suggest that the presence of an H3K27ac signal near TSS is positively correlated with the gene expression levels at the same sample level (Fig. 3a), whereas changed H3K27ac signals near TSS and differentially expressed genes between patients and controls are not always correlated (Fig. 3b).

### Annotated genes from DARs are enriched for remodeling-associated biological processes

**Fig. 4:**
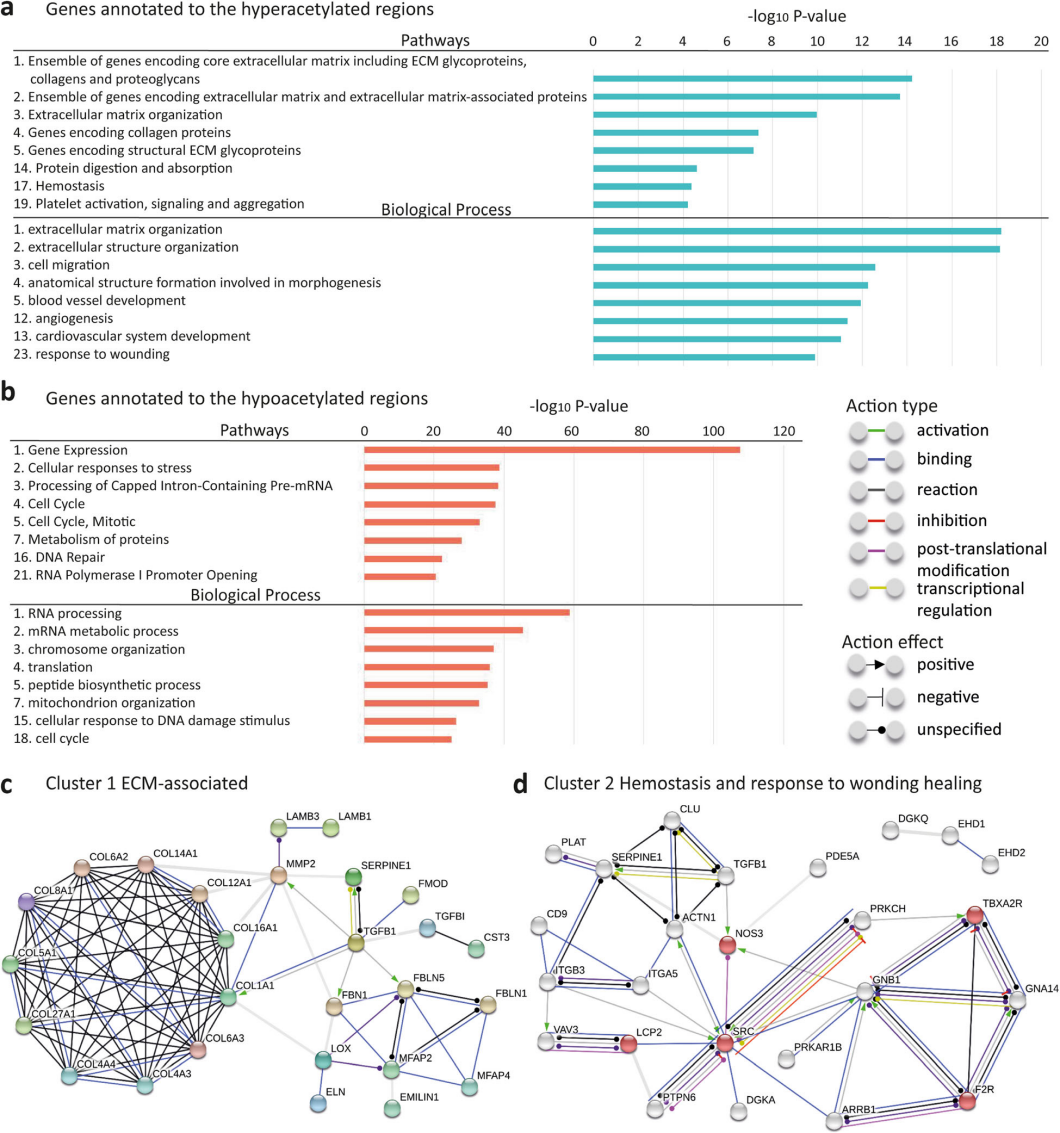
Functional analysis based on genes identified from DARs. **a** Top enriched Pathways and GO Biological Process based on 1924 annotated genes in the vicinity of hyperacetylated regions that locate within a ± 5 kb range from the transcription start site. **b** Top enriched Pathways and GO Biological Process based on 5885 annotated genes in the vicinity of hypoacetylated regions that locate within a ± 5 kb range from the TSS. **c** Interaction of extracellular matrix-related genes, including TGFβ pathway associated genes and a cluster of collagen encoding genes detected (STRING). **d** Interaction of genes that are involved in the wound healing process, gene-encoded proteins in platelet activation are indicated in red nodes (STRING)

Next, we studied their enriched biological functions and pathways (Supplementary Figure 5). The most enriched biological functions in genes close to the hyperacetylated regions were linked to extensive ECM regulation and cell binding (Fig. 4a). STRING analysis identified key gene-encoded proteins based on all genes involved in ECM-related processes, including *TGFB1*, *FBN1*, *MFAP2*, *FBLN5*, *MFAP4*, and a cluster of collagen encoding genes (Fig. 4c). Likewise, genes involved in enriched Pathway and Biological Process “Hemostasis” and “response to wounding” were annotated by STRING to provide information on interactions in the wound healing process (Fig. 4d). The most enriched GO Biological Processes in the hypoacetylated regions were summarized in Fig. 4b, and a considerable number of them were involved in (transcriptional) regulation of protein synthesis.

### Acetylation levels and transcriptional levels per cell-type-specific marker

**Fig. 5:**
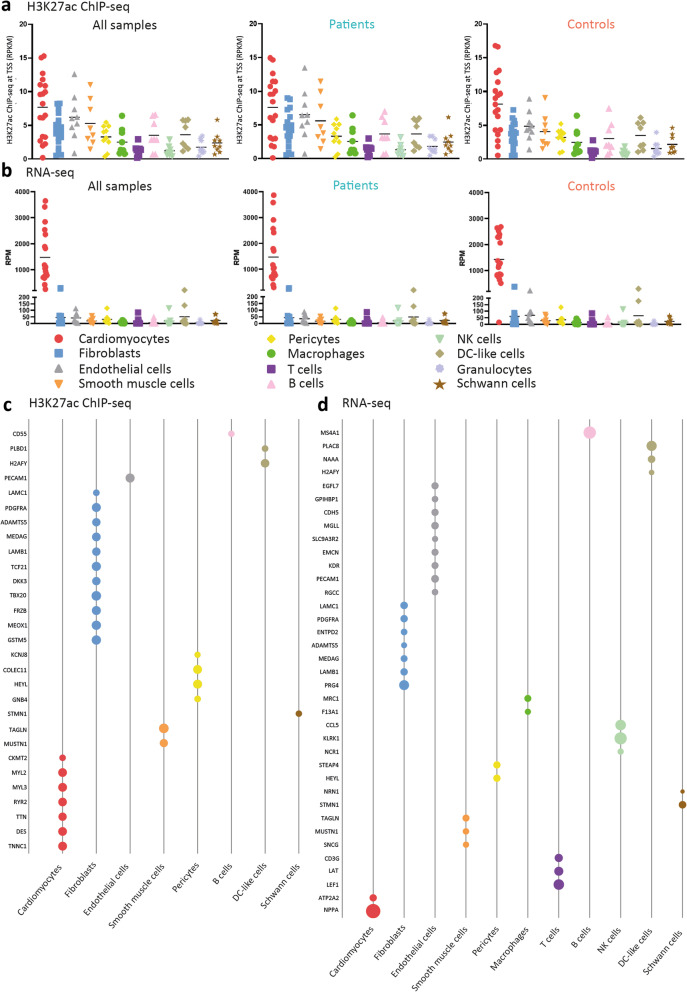
The promoter acetylation and mRNA expression levels of cell-type-specific markers. **a** The acetylation levels of all makers per cell type in all samples, patient samples, or controls. **b** The mRNA expression levels of all markers per cell type in all samples, patient samples, or controls. **c** Markers, which showed significantly different acetylation levels at the 2.5 kb upstream window from the transcription start sites between patients and controls, were shown. Each point represents a marker, and the fold change value was used and corresponds to the point size. **d** Markers, which showed significantly different mRNA expression level between patients and controls, were shown. Each point represents a marker, and the fold change value was used and corresponds to the point size. RPM, reads per million, which were normalized

To estimate from which cardiac cell type the H3K27ac signal and RNA-seq data are mainly derived, we further examined H3K27ac occupancy and mRNA expression level of cell-type-specific markers in our bulk data set. First, we collected validated markers for cardiomyocytes and 11 types of non-myocytes as shown by two recent studies in murine hearts in which single-cell RNA sequencing was used [32]. The promoter acetylation level and the mRNA expression level of each marker per cell type in all of our samples, in patients only, and in controls only, are shown in Fig. 5a and b, respectively. Interestingly, the acetylation levels of these marker genes remained consistent among the 12 cell types, and they were comparable between patients and controls. Their transcriptional levels were also consistent among the 11 non-myocyte cell types and remained comparable between patients and controls. However, the mRNA expression levels of cardiomyocyte-specific markers in all samples were more profoundly expressed when compared with all non-myocyte markers, and this expression pattern was also observed in both patients and controls. We further highlighted the individual markers whose nearest upstream regions were significantly differentially acetylated (Fig. 5c) and markers whose mRNA expression levels were significantly differentially expressed between patients and controls (Fig. 5d). Out of 19 cardiomyocytes markers, the nearest chromatin regions of 7 markers were significantly differentially acetylated, while another 2 markers were significantly differentially expressed (Fig. 5d). For the non-myocytes, some markers showed significant changes in both acetylation and transcriptional levels (i.e., fibroblast marker *LAMC1*). The comparable acetylation and mRNA expression levels of cell-type-specific markers between patients and controls suggest that the cell compositions in healthy and patient-derived cardiac tissues were not significantly different. Given the strikingly higher mRNA expression levels of markers for cardiomyocytes when compared with markers for 11 non-myocytes cell types, the RNA data were more likely to be representative of the transcriptional profile of the cardiomyocyte pool.

### Discovery of transcription factor binding motifs (TFBMs) and candidate TFs in regulating myocardial remodeling

**Fig. 6:**
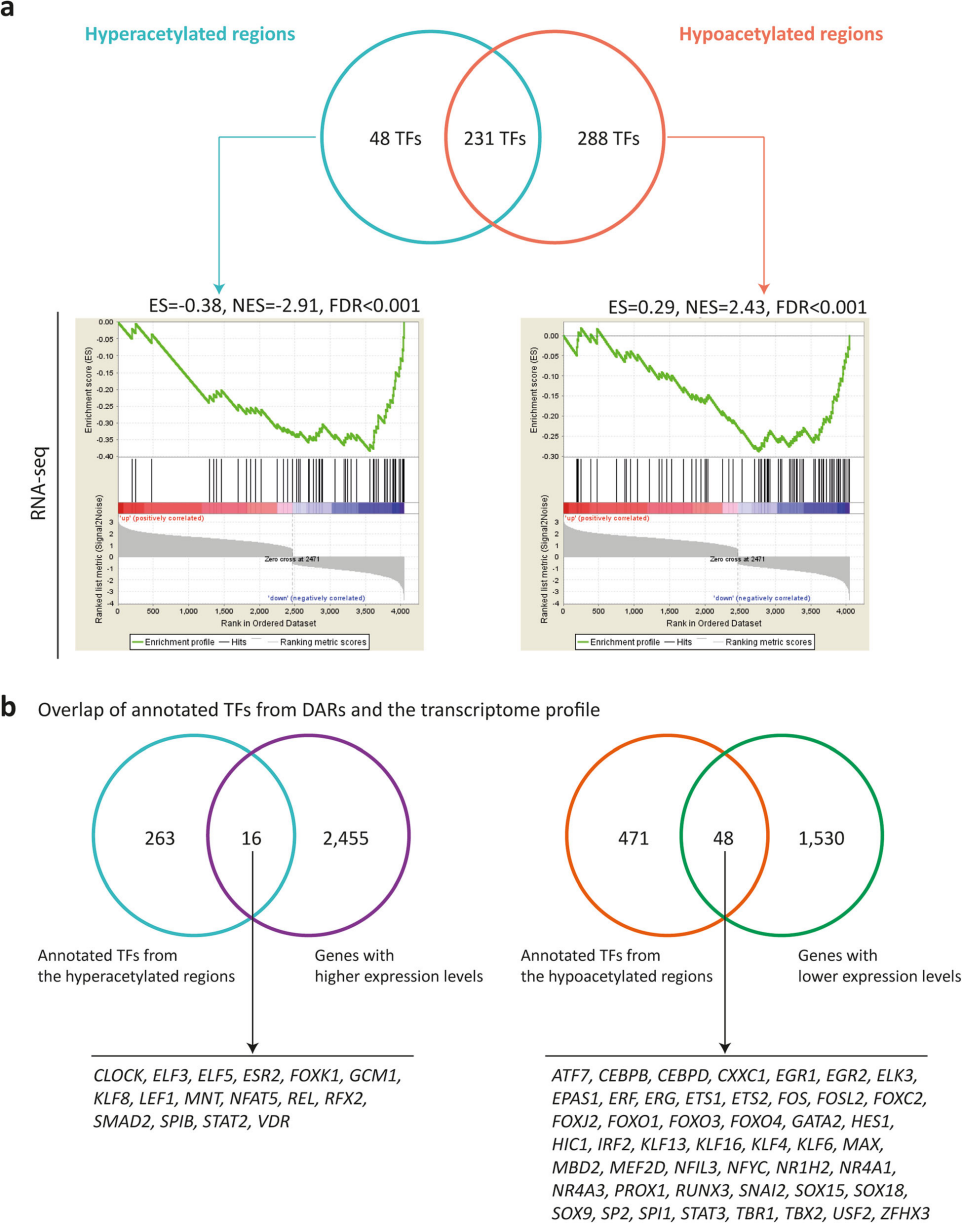
Transcription factors (TFs) annotated from enriched transcription factor binding motifs (TFBMs) in the differentially acetylated regions (DARs). **a** A Venn diagram shows the overlap of motif-encoded TFs that were linked to TFBMs found to be enriched in DARs. In the graph below are shown the gene set enrichment analysis of genes encoding these TFs with differentially expressed genes between patients and controls. Differentially expressed genes per study are ranked by their fold changes and shown on the *x*-axis. The running correlation throughout the gene set is shown by the curve (green) and the running enrichment score (ES) is shown in the *y*-axis. Enrichment score normalized for gene set size (NES) and the false discovery rate (FDR) are shown above each plot. Black bars indicate annotated genes from differentially acetylated regions that are presented among the transcriptome profiles. **b** A subset of TFs that present the same direction in the acetylation activity and mRNA expression change in remodeled myocardium as compared to the controls

To identify the possible upstream regulators of the observed chromatin activity changes, we investigated the occurrence of TFBMs in DARs. In total, we identified 304 and 540 TFBMs in the hyper- and hypoacetylated regions, respectively (Supplementary Table 5). We could assign 48 and 288 TFs with the ability to bind to TFBMs that were exclusively overrepresented in the hyper- and hypoacetylated regions, respectively (Fig. 6a). Also, we could assign 231 TFs to TFBMs that were overrepresented in both hyper- and hypoacetylated regions. Genes coding for TFs that were identified in the hypoacetylated regions significantly correlated with genes with lower mRNA expression levels in patients when compared with controls (FDR < 0.001, Fig. 6a). However, genes coded for the predicted TFs from the hyperacetylated regions were not enriched in the pool of upregulated genes. By overlapping the annotated TFs in the hyperacetylated regions (48 + 231 = 279 in total) with the differentially expressed genes, we identified 16 TFs (e.g., *SMAD2* and *ELF3*) that were enriched in the hyperacetylated regions and were also presented with higher mRNA levels in patients when compared with controls. In addition, the overlap analysis showed that 48 annotated TFs (e.g., *CEBPB* and *KLF6*) in the hypoacetylated regions (288 + 231 = 519 in total) also showed lower mRNA levels in patients versus controls (Fig. 6b).

To further investigate if the observed epigenetic regulation is mainly derived from the cardiomyocyte pool, we studied the involvement of those 64 TFs, which bind to enriched TFBMs and showed altered mRNA expression levels in remodeled hearts when compared with controls, using a gene set in hypertrophic human stem cell-derived cardiomyocytes from a published study [33]. Four suppressed TFs identified in our study (*EGR1*, *EGR2*, *EPAS1*, and *ZFHX3*) also showed lower mRNA expression levels in mechanically induced hypertrophic cardiomyocytes when compared with controls. This finding suggests that some of our identified TFs could be highly relevant for cardiomyocytes.

## Discussion

In this study, we report the first histone acetylome profile based on H3K27ac ChIP-seq to reveal changed chromatin activities in remodeled non-failing myocardium from AS patients as compared to healthy donor hearts. We identified 5634 hyperacetylated regions and 5724 hypoacetylated regions upon myocardial remodeling. We also showed that reorganized chromatin regions, which changed in the same direction in response to myocardial remodeling, tended to cluster together. Genes annotated to these altered regulatory regions are associated with the development of fibrosis formation and the regulation of cell metabolism, and their acetylation levels are correlated with their expression levels between patients and controls, as shown in the mRNA data obtained from our study and published studies. We further demonstrated the acetylation level and mRNA level of markers specific for cardiomyocytes and 11 non-myocyte cell populations. Lastly, by integrating the acetylome and transcriptome datasets, we identified a list of TFs that presented with the same direction in the acetylation change and the mRNA expression changes in remodeled myocardium. Several of these TFs, such as *EGR1* and *EPAS1*, have already been shown to be downregulated in hypertrophic cardiomyocytes [33]. These TFs could be promising candidates in elucidating the key TFs-DNA interactions that are involved in the pathomechanisms in remodeled myocardium, and may serve as potential druggable targets for future epigenetic therapies.

Recent studies demonstrate that alterations in chromatin domains could lead to altered gene expression and disease [34]. A review from van Steensel and Furlong on the spatial organization of the genome showed that the interaction between enhancer and promoter is often found within the same TAD and subsequently regulates transcription [35]. They further indicated the possible influence of active transcription on the chromatin architecture within the subdomains of TADs (sub-TADs). However, little is known about TAD regulation in human myocardium. A previous study mapped the global chromatin structural changes in heart failure using a murine model [36]. Here, we showed that adjacent regions, which might serve as putative (sub-)TADs, were more likely to interact in the same fashion, indicating well-organized chromatin structures that could physically facilitate the interactions of regulatory domains. Yet, additional studies are required to further elucidate these identified adjacent regions as (sub-)TADs using circular chromosome conformation capture and sequencing. We are the first to demonstrate that differences of H3K27ac occupancy are not limited to quantitative and qualitative analyses of detected regions between healthy and diseased conditions, but also provide insights into the highly ordered chromatin structural rearrangements in response to the disease.

A recent epigenetic study showed that gene expression levels in cardiomyocytes from end-stage failing human hearts correlated with levels of active histone marks, especially the H3K27ac mark [17]. Consistent with previous studies, we also observed that the H3K27ac occupancy level correlated well with both RNA polymerase II occupancy level using RNAPII ChIP-seq and gene expression level using RNA-seq per sample. Using differentially expressed gene profiles obtained in the present study and from two published studies, we further observed that the annotated gene set in DARs was statistically significantly correlated with differentially expressed genes. Nevertheless, it is important to note that not all datasets were significantly correlated, which could be due to the different number of genes between the gene set and the expression database and the different turnover ratios between histone acetylation and mRNAs [37]. Previous epigenomic studies that aimed to identify novel targets in heart diseases also clearly demonstrated the importance of combining transcriptome data with epigenetic data to reveal different perspectives of the epigenetic regulation of the disease [38]. For example, genes, which showed the same direction between H3K27ac signal and expression level in patients and controls (i.e., chr1:209,973,800-209,977,199/*IRF6* and chr16:56,639,200-56,646,949/*MT2A*), could improve our understanding of the affected biological networks and serve as potential targets for chromatin modifiers and/or gene therapy-related strategies in remodeled hearts.

The most enriched biological processes by genes annotated to the hyperacetylated regions are related to ECM regulation. ECM organization is an important component of cardiac remodeling, and extensive cardiac fibrosis is a hallmark of maladaptive hypertrophy [39, 40]. TGFβ, a key mediator in ECM formation, is regulated by the activation of SMAD3 and the phosphorylation of ATF2 [41]. We also identified *SMAD3* in the hyperacetylated regions and *ATF2* in the hypoacetylated regions. An important TGF*β*1-related signaling cascade is activated in cardiac fibroblasts from patients with coronary artery disease when compared with controls, and the inhibition of IL11, a main downstream target of TGF*β*1, leads to reduced fibrosis in the heart using murine model [42]. In line with these findings, we also observed a higher mRNA expression level of *TGFβ1* and *IL11* in patients versus controls. Furthermore, ECM homeostasis is also regulated by a tight balance between matrix metalloproteinases (MMPs), which degrade ECM proteins, and tissue inhibitors of metalloproteinases (TIMPs) [39, 43]. We annotated *MMP2*, *MMP23A*, *TIMP3*, and *TIMP4* in hyperacetylated regions and a group of MMPs that showed higher mRNA expression levels in patients. These data imply the activation of genes involved in fibrogenic pathways at the chromatin level and transcriptional level, which is in line with previous studies that reveal a strong fibrogenic response in left ventricular remodeling [39]. Although we performed bulk sequencing and the histological staining showed extensive fibrosis in remodeled myocardium, it is important to note that cardiomyocytes also express ECM-related genes [44]. Transcriptional processes and cell cycle were mostly enriched by genes annotated in the hypoacetylated regions, indicating the downregulation of cell metabolism in patients.

Cellular mechanotransduction has been demonstrated in the heart, and mechanical forces are greatly increased in AS [45, 46], suggesting the potential influence of mechanotransduction during myocardial remodeling. Among various pathways that are involved in the mechanical signaling, the Hippo pathway and its regulation of the YAP/TAZ protein complex have been highlighted to play a central role [46–48]. The core regulators of the Hippo pathways, including MST1/2, SAV1, TAO-family kinases (TAO), and MAP 4 K kinases, activate LATS1 and LATS2 kinases, which subsequently phosphorylate YAP/TAZ, thereby stimulating proteolysis of the YAP/TAZ complex and limiting its transcription regulatory capacity. In our study, we observed that the chromatin acetylation levels of MST1, MST2 (also known as STK3), and TAOK2 were higher in patients than controls. However, the mRNA expression levels of these genes remained comparable between both groups. In addition, the chromatin acetylation levels and the mRNA expression levels of MAP 4 K2-5 members and LAST1/2 were not significantly changed in patient hearts when compared with controls. Downstream targets YAP and TAZ also showed similar chromatin acetylation and mRNA expression levels in patient versus control hearts (Supplementary Table 8). As we have conducted bulk sequencing, any subtle changes in the Hippo pathway on single-cell type level might remain hidden. Furthermore, changes in pathways related to mechano-activation might mainly present itself on protein level (e.g., level of phosphorylation of the YAP/TAZ complex). Future single-cell (type)-sequencing-based studies in combination to proteomics studies are needed to further elucidate the effect of mechanical forces on different cell types in myocardial remodeling.

Sex differences in myocardial remodeling have been identified by previous studies [49–52]. For example, there is a higher expression level of genes involved in fibrosis and inflammation in male hypertrophic hearts compared with female hypertrophic hearts [53, 54]. In the present study, we did not observe a sex-based clustering of hearts with concentric remodeling based on differentially acetylated regions and differentially expressed genes (Supplementary Figure 6). This was most likely due to the limited number of healthy hearts from women and men (*n* = 2 and *n* = 3, respectively). Additionally, unlike left ventricular hypertrophy, concentric remodeling is defined as a normal cardiac mass despite a thickened left ventricular wall [55]. Therefore, the maladaptation of myocardial remodeling is worse in left ventricular hypertrophy than in concentric remodeling, which could also lead to the different observations on the sex dimorphism between this study and previous studies from us and others. Last but not least, the difference in location from which cardiac specimens were collected between our study and previous studies may also play a role. Combined, it is likely that the sex-specific acetylome and transcriptome were less profound in hearts with moderate myocardial remodeling than advanced myocardial remodeling. Certainly, future studies are needed to investigate sex differences with additional healthy control samples and a larger patient group size.

Notably, several TF-encoding genes that were identified in the present study have previously been shown affected on the gene expression level, either in animal models with remodeled myocardium by our group or in hypertrophic human stem cell-derived cardiomyocytes by others. Previous studies showed a list of TFs with altered mRNA expression levels in pigs with remodeled left ventricle when compared with controls, such as the upregulation of *SMAD3* and *SPIB*, and the downregulation of *FOSL2*, *MEF2D*, *STAT3*, and *ATF7* [56, 57]. These studies also identified TFs with significantly affected binding activities in hypertrophic conditions using protein/DNA array analysis, such as *EGR1*, *ETS1*, and *ETS2* [57]. In line with these reports, these same TFs were also found in the present study, which showed matched acetylation and/or mRNA levels changes in remodeled hearts when compared with controls. Furthermore, the mRNA expression levels of several TFs also showed consistent changing patterns in hypertrophic human stem cell-derived cardiomyocytes when compared with controls, including *EGR1*, *EGR2*, *EPAS1*, and *ZFHX3* [33]. The involvement of these TFs in cardiac remodeling has also been well established by other studies [58, 59].

Taken into account that we performed tissue-level bulk sequencing and the cellular heterogeneity in the heart, our data do not represent changes only in cardiomyocytes. Recent studies have been using single-cell sequencing to further reveal the transcriptional landscape per cell type in the heart, including cardiomyocytes, fibroblasts, and endothelial cells [60]. However, these studies either sorted cells from freshly harvested hearts or they isolated nuclei from snap-frozen human hearts. Due to the difficulty of obtaining intact cells from snap-frozen hearts, no study has shown the epigenome and/or transcriptional regulation at the single-cell resolution in remodeled human myocardium. Nomura and colleagues demonstrate that the transcriptional profiles in isolated cardiomyocytes are well correlated with the bulk transcriptional profiles of murine hearts [61]. Interestingly, by examining the mRNA expression level of markers for cardiomyocytes and 11 non-myocytes in our bulk sequencing data, we observed that cardiomyocyte markers displayed around 28–225 fold higher expression levels than all other cell type markers, suggesting that the majority of signals at the transcriptional level was likely derived from cardiomyocytes. It is important to note that cell-type-specific markers for cardiomyocytes and 11 non-myocyte cell types were based on findings derived from two single-cell sequencing studies using murine hearts. Although some studies showed conserved global gene expression between human and mouse hearts, others have shown a poor correlation between species [62–64]. Therefore, the analysis is limited in the way that using cell-type-specific markers of murine hearts may not translate one-to-one with marker expression on human cardiac cells. Additionally, these markers were not corrected to the number of cells per cell type. Yet, this observation is in line with the well-correlated transcriptional profiles between cardiomyocytes and bulk cardiac tissues shown by Nomura et al. [61]. Although the acetylation level of cardiomyocyte markers seemed to be slightly higher than other cell type markers, the profound mRNA expression pattern was not observed, suggesting additional regulatory machinery between histone acetylome and transcriptome. Additional studies on cell-type-specific enhancers are still needed to improve our understanding of the chromatin acetylation changes per cell composition. Nevertheless, top enriched TFBMs in cardiomyocytes from remodeled murine hearts after transverse aortic constriction were also obtained in our study [61]. The paracrine mechanisms between cardiomyocytes and non-cardiomyocytes play an important role in regulating cardiac function [65]. The direct cell-to-cell crosstalks (i.e., the TGF*β* signaling between cardiac fibroblasts and cardiomyocytes), and indirect cell-to-ECM crosstalks (i.e., via the integrin-related signaling), contribute throughout the (mal) adaptive process of cardiac remodeling [66]. Our data could offer valuable and extensive information on these biological crosstalks.

## Conclusions

In conclusion, the identified H3K27ac chromatin regulation landscape shows significant changes in remodeled myocardium as compared to controls. Enriched genes and TFBMs in the differentially acetylated regions highlight known disease-associated processes, such as fibrosis. TFs that exhibited the same direction in their acetylation and mRNA expression changes might shed light on the upstream signaling by providing the candidates to build and validate putative key TF-DNA networks. Taken together, the dataset as presented here from our study provides valuable new information and aid in the discovery of key pathways and transcription mechanisms in the underlying disease etiology. These findings also demonstrate the value of genome-wide chromatin analysis in understanding the molecular regulation and etiology of myocardial remodeling and cardiovascular disease in general. With the development of single-cell sequencing techniques in biobanked patient material and the accumulating evidence of cell-to-cell heterogeneity within the very same cell population in response to myocardial remodeling [29], we might gain knowledge on the chromatin profile at the single-cell resolution. This will allow us to examine gene expression, protein level, and the crosstalk between multiple cell types and to investigate the cell-to-cell variations after chromatin regulatory activity changes and structural reorganization, further contributing to the understanding of DNA mutations in regions with regulatory function and new drug target identification for future therapies.

## Materials and methods

### Study design and sample information

This study was approved by the Biobank Research Ethics Committee of University Medical Center Utrecht (protocol number 12/387), the Ethics Committee of UK National Research Ethics Service (07/H0715/101), and the Washington University School of Medicine Ethics Committee (Institutional Review Board). Written informed consent was obtained or in certain cases waived by the ethics committee when acquiring informed consent was not possible due to the death of the individual (control samples). All patients with cardiac remodeling due to AS were recruited prior to pre-operative assessment, which included a comprehensive evaluation with clinical history, resting blood pressure, 6-minute-walk test, electrocardiogram, transthoracic 2D-echocardiogram, and cardiac magnetic resonance (CMR). Inclusion criteria were patients > 18 years with severe AS (2 or more of the following: aortic valve area < 1 cm^2^, peak pressure gradient > 64 mmHg, mean pressure gradient > 40 mmHg, aortic valve velocity ratio < 0.25) undergoing aortic valve replacement ± coronary artery bypass grafting. Exclusion criteria were pregnancy/breastfeeding, estimated glomerular filtration rate < 30 mL/min/1.73 m^2^, CMR incompatible devices, inability to complete the protocol, previous valve surgery, or severe valve disease other than AS. Patient samples (*n* = 20, 13 men and 7 women) were obtained by either intraoperative scalpel or Tru-Cut needle biopsy. Due to the difficulty in obtaining healthy donor hearts, the number of control samples was limited (*n* = 5, 3 men and 2 women), and they were either autopsy material or non-transplanted donor hearts without signs of cardiac remodeling. Myocardial samples were collected and snap-frozen in liquid nitrogen, and stored at − 80 °C. For all samples, 8-μm-thick histological slices were stained for hematoxylin-eosin (Supplementary Figure 1). For detailed information per sample, please refer to Supplementary Table 6A and B.

### Chromatin immunoprecipitation and sequencing

To study the changes of histone acetylome between patient and control samples, we isolated chromatin from all frozen cardiac tissues. Briefly, all samples were sectioned at the thickness of 10 μm and chromatin was isolated using the MAGnify™ Chromatin Immunoprecipitation System kit (Life Technologies) according to the manufacturer’s instructions. The anti-histone H3K27ac antibody (ab4729, Abcam) was used for immunoprecipitation. Captured DNA was purified using the ChIP DNA Clean & Concentrator kit (Zymo Research). Libraries were prepared using the NEXTflex™ Rapid DNA Sequencing Kit (Bioo Scientific). Samples were PCR amplified, checked for the proper size range and for the absence of adaptor dimers on a 2% agarose gel. Barcoded libraries were sequenced 75 bp single-end on Illumina NextSeq500 sequencer. Sequencing reads were mapped against the reference genome (hg19 assembly, NCBI37) using the BWA package (mem –t 7 –c 100 –M –R) [67]. Multiple reads mapping to the same location and strand were collapsed to single read, and only uniquely placed reads were used for peak-calling. Peaks/regions were called using Cisgenome 2.0 (–e 150 -maxgap 200 –minlen 200) [68]. Region coordinates from all samples were stretched to at least 2000 base pairs and collapsed into a single common list. Overlapping regions were merged based on their outmost coordinates. Only regions supported by at least 2 independent datasets were further analyzed. Autosomal sequencing reads from each ChIP-seq library were overlapped back with the common region list to set the H3K27ac occupancy for every region-sample pair.

To further verify the detected H3K27ac signal corresponds to the transcription level, we incubated chromatins from 4 controls with anti-RBP1 antibody (PB-7G5), a subunit of RNA polymerase II (RNAPII), and performed the same ChIP-seq procedure [69]. To visualize the correlation between H3K27ac and RNAPII - log_2_, transformed reads per kilobase per million (RPKM) in the gene body were correlated to H3K27ac ChIP-seq signal on gene promoters (± 2.5 kb around the TSS). For more information, please refer to Supplementary Figure 3 and Supplementary Table 7.

### Identification of differentially acetylated regions

Next, we identified regions with different H3K27ac occupancy levels between patients and controls using DESeq2 with the standard settings (FDR Benjamini & Hochberg *P*_adj._ < 0.05) [70]. Supervised hierarchical clustering was performed with quantile normalized (limma::normalizeQantiles() function in R), log_2_ transformed, and median centered read counts per common region. To avoid the log_2_ transformation of zero values, one RPKM was added to each region. Genomic regions in which hyper- or hypoacetylated peaks/regions located in patients when compared with controls were defined based on the cutoff of adjusted *p* < 0.05 and are referred to as “DARs.” To visualize the clustering of the samples based on the median centered and log_2_ transformed levels of acetylation in DARs, we used heatmap.2() command from gplots package in R.

### Identification of tandem regulated chromatin domains

Next, we studied the direction of the acetylation changes in DARs to verify the chromatin spatio-temporal dynamics that play an important role in gene regulation. Adjacent regions that had the same fold change (FC) direction and the absolute log_2_FC > 0.4 were considered as possible (sub)compartments due to the spatial organization of the genome. Control datasets were generated by shuffling the acetylation fold change and significance values while retaining the locations and distances of adjacent peaks.

### Region-to-gene annotation and Gene Ontology enrichment analysis

We also performed in silico region-to-gene annotation using a conservative window of ± 5 kb from the transcription start site (TSS). Annotated gene sets were studied for their biological functions using ToppGene Suite tool ToppFun (default setting: Probability density function, FDR correction, *p* value cutoff of 0.05 and gene limit set between and including 1 and 2000 per pathway) [71]. STRING was used to identify the key gene-encoded proteins and their interactions within selected gene sets (minimum required interaction score: high confidence (0.700)) [72].

### Discovery of transcription factor binding motifs and their putative networks

In DARs, we also identified enriched transcription factor binding motifs (TFBMs) that could elucidate the putative networks between motif-encoded transcription factors and genes. Briefly, repeat masked sequences of the DARs were first overlapped with DNAse hypersensitivity site analysis from cardiac samples from the ENCODE project [28]. DNA sequences of those overlapping DNAse sites and control shuffled sequences were taken by MEME Suite AME tool [73] under the following settings: motif database: human (HOCOMOCO v10); background model frequencies: 0.25, 0.25, 0.25, and 0.25; total pseudo-count added to a motif columns: 0.25; Wilcoxon rank-sum test with threshold *P* < 0.05; number of multiple tests for Bonferroni correction: #Motifs × #Partitions Tested = 641 × 1 = 641. By overlapping identified motifs and annotated genes from DARs, we obtained a list of genes encoding for TFs that were potentially master regulators of myocardial remodeling at the chromatin level.

### RNA sequencing and gene expression analyses

Besides the correlation between the H3K27ac signal and RNAPII occupancy level, we also examined the H3K27ac signal in correlation with the transcriptome level as revealed by RNA sequencing (RNA-seq). Briefly, RNA was isolated from 4 controls according to the manufacturer’s instructions (BioLine) with minor changes. Libraries were generated using NEXTflex^TM^ Rapid RNA-seq Kit (Bio Scientific) and sequenced by the Nextseq500 platform (Illumina). Sequenced reads were annotated as described previously [69]. Reads per kilobase million (RPKM) that presented in all samples were included, and genes with the mean RPKM > 0.5 were considered expressed. RPKM on the log_2_ scale were correlated to call reads in H3K27ac ChIP-seq using R.

To compare the transcriptional landscapes between patient and control samples, we performed RNA-seq using the CEL-seq protocol—an adapted single-cell RNA-seq method that overcomes the challenge of the low input materials due to the limited biopsy size [74]. Briefly, 10 ng RNA from each sample was used. Primer design, linear mRNA amplification, library construction, and sequencing were performed as described previously [75]. Raw read file per sample was used, and differentially expressed genes between patient and control hearts were identified using the DESeq2 [70] within the Galaxy environment under the default settings. A *p* value cutoff of 0.05 was used.

### Gene set enrichment analysis (GSEA)

We performed gene set enrichment analyses using GSEA software v3.0 [76] and studied the enrichment of annotated genes from DARs in relation to the gene expression levels between patient and control hearts. TFs, from which their TFBMs were enriched in DARs, were also examined in the transcriptome profile. Briefly, we included genes with significantly different expression levels as revealed by the CEL-seq in the present study and two published studies using microarray [11, 12]. Differentially expressed genes in each of three transcriptome profiles were ranked based on their fold change and uploaded to GSEA as the expression datasets. The annotated gene set from hyper- or hypoacetylated regions was accessed for its positive or negative enrichment throughout each expression dataset with the standard settings.

### Hypertrophic markers in cardiomyocytes during remodeling

We also investigated candidates that could play an important role in cardiomyocytes from the bulk sequencing data. Briefly, a list of genes that showed the same direction of changes at the histone acetylation level and mRNA expression level was selected from our study. These genes were overlapped with genes with altered mRNA expression levels in hypertrophic human stem cell-derived cardiomyocytes due to mechanical stress [33].

## Supplementary information

**Additional file 1.** Supplementary Figure 1. An overview of the workflow in this study. †Detailed information of samples used in H3K27ac ChIP-seq, RNAPII ChIP-seq, and RNA-seq are listed in Supplementary Table 2. *: Standard RNA-seq and adjusted RNA-seq (3′-RNA-seq) were both performed, detailed information is shown in Supplementary Table 7.

**Additional file 2.** Supplementary Figure 2. Histology of cardiac tissues used in this study. Overview of representative slides stained with hematoxylin-eosin from cardiac samples in control and patient groups are shown. Higher magnification showing normal myocardium in control (panel A) and patient myocardium with hypertrophy of cardiomyocytes and interstitial fibrosis in the patient sample (panel B, both × 40 magnification).

**Additional file 3.** Supplementary Figure 3. Correlation plots between H3K27ac ChIP-seq (red) with RNAPII ChIP-seq (blue) and RNA-seq datasets (blue), respectively. Correlations in four control samples (from a to d) used in this study. Gene expression rank is based on RPKM values.

**Additional file 4 **Supplementary Figure 4. The mRNA expression level of cell-type-specific markers. Genes labeled red and blue were significantly up- and downregulated in patients versus controls (*p* < 0.05).

**Additional file 5.** Supplementary Figure 5. Gene Ontology (GO) analysis of genes annotated to DARs using ToppFun. a Molecular function enrichment for hyperacetylated regions, b Biological process for hyperacetylated regions, c Cellular component for hyperacetylated regions, d Molecular function for hypoacetylated regions, e Biological process for hypoacetylated regions, f Cellular component for hypoacetylated regions.

**Additional file 6.** Supplementary Figure 6. Sex-specific H3K27ac acetylome and transcriptome profiles between male and female patients with concentric remodeling. a Principal component analysis (PCA) plot showing the clustering of man and woman cardiac samples based on H3K27ac profiles (using 500 regions with the highest variance). b PCA plot showing the clustering of man and woman cardiac samples based on the transcriptome profiles (using 500 genes with the highest variance).

**Additional file 7 **Supplementary Table 1. Regions with different H3K27ac occupancy in patients compared with the control group (adjusted *p* value < 0.05).

**Additional file 8.** Supplementary Table 2. a Identified active tandem regulated chromatin domains (TRCDs) in the short range (5Kb–100Kb) in patients when compared with controls. b Identified active TRCDs in the long range (100Kb–1 Mb) in patients when compared with controls. C Identified repressed TRCDs in the short range (5Kb–100Kb) in patients when compared with controls. d Identified repressed TRCDs in the long range (100Kb–1 Mb) in patients when compared with controls. e Randomized TRCDs in the short range (5Kb–100Kb) in patients when compared with controls. f Randomized TRCDs in the long range (100Kb–1 Mb) in patients when compared with controls.

**Additional file 9.** Supplementary Table 3. a Genes in the vicinity of hyperacetylated regions using a ± 5 kb window. b Genes in the vicinity of hypoacetylated regions using a ± 5 kb window.

**Additional file 10.** Supplementary Table 4. Differentially expressed genes between patients and controls.

**Additional file 11.** Supplementary Table 5. Identified transcription binding motifs from differentially acetylated regions.

**Additional file 12.** Supplementary Table 6. a An overview of general information of all included human cardiac samples. b An overview of detailed clinical parameters of the included AS patients.

**Additional file 13.** Supplementary Table 7. Overview of included samples in H3K27ac ChIP-seq, RNAPII ChIP-seq, and RNA-seq experiments.

**Additional file 14.** Supplementary Table 8. The chromatin acetylation changes and the mRNA expression changes of key regulators and targets in the Hippo pathway.

## Data Availability

All relevant data are available within the article and the supplementary files. Because of the sensitive nature of the data collected for this study, requests to access the dataset from qualified researchers trained in human subject confidentiality protocols may be sent to the corresponding authors.

## Abbreviations

ChIP-seq: Chromatin immunoprecipitation and sequencing H3K27ac Histone 3 lysine 27 acetylation RNA-seq RNA sequencing TFs Transcription factors AS Aortic stenosis DARs Differentially acetylated regions TADs Topologically associating domains GSEA Gene set enrichment analysis TSS Transcription start site ECM Extracellular matrix TFBMs Transcription factor binding motifs MMPs Matrix metalloproteinases TIMPs Tissue inhibitors of metalloproteinases CMR Cardiac magnetic resonance FC Fold change RPKM Reads per kilobase million
