# Supplementary figures and images for: H3K27ac acetylome signatures reveal the epigenomic reorganization in remodeled non-failing human hearts
Source: Clin Epigenetics. 2020 Jul 14;12:106. doi: 10.1186/s13148-020-00895-5 (PMC7362435; doi:10.1186/s13148-020-00895-5)

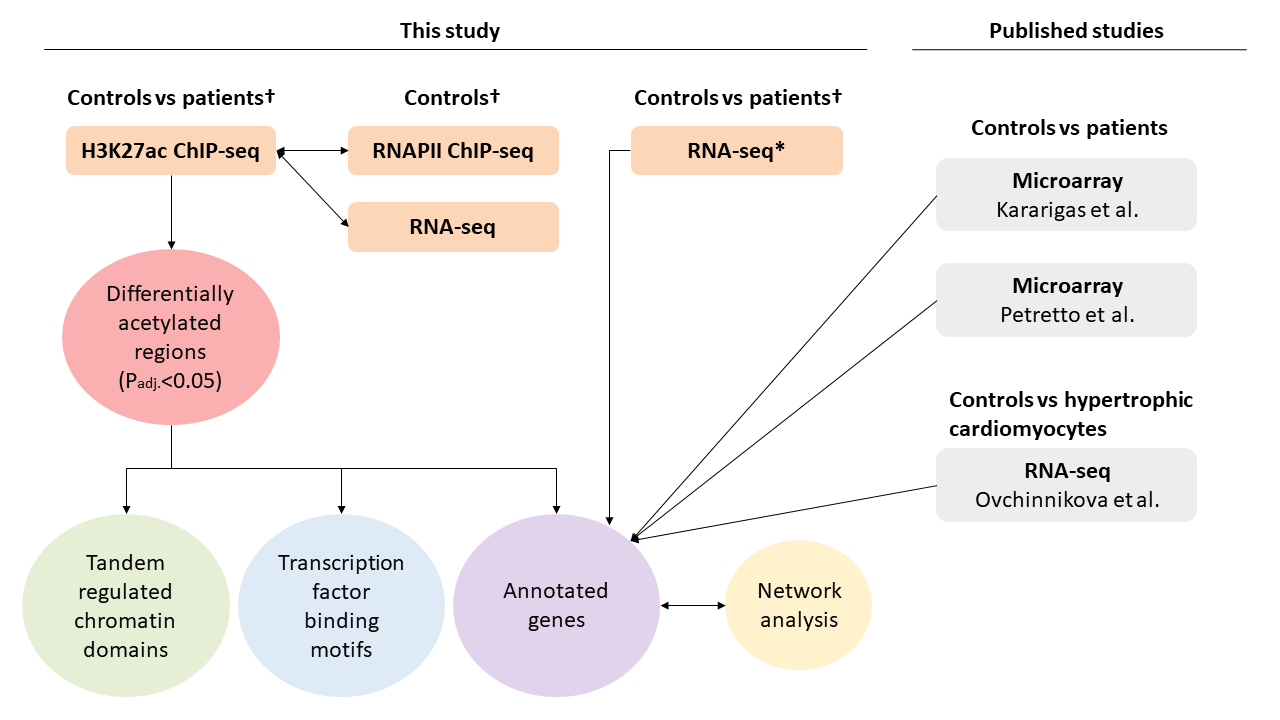

Supplement: Supplementary file 1 — Additional file 1. Supplementary Figure 1. An overview of the workflow in this study. †Detailed information of samples used in H3K27ac ChIP-seq, RNAPII ChIP-seq, and RNA-seq are listed in Supplementary Table 2. *: Standard RNA-seq and adjusted RNA-seq (3′-RNA-seq) were both performed, detailed information is shown in Supplementary Table 7. [file 13148_2020_895_MOESM1_ESM.tif]

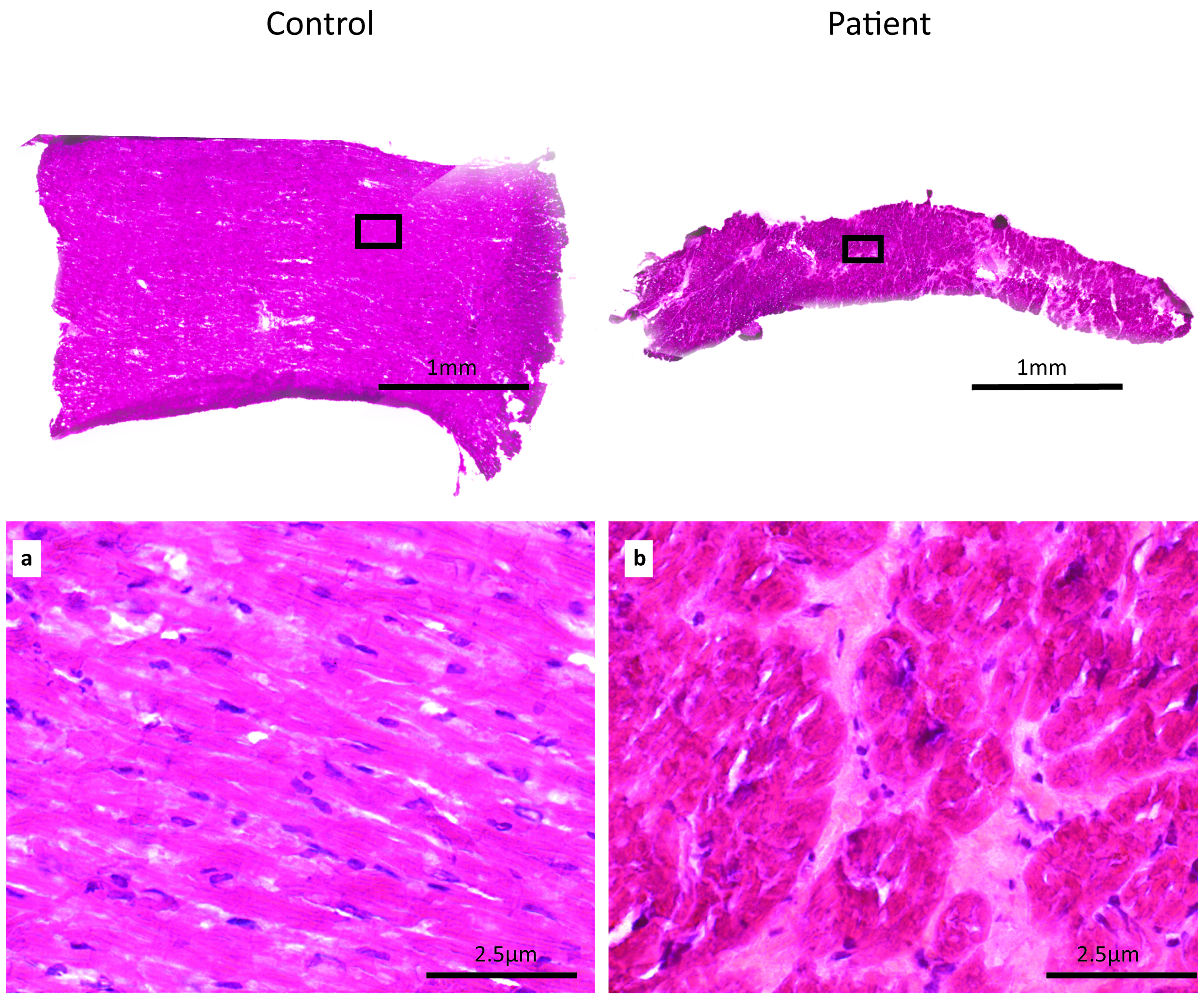

Supplement: Supplementary file 2 — Additional file 2. Supplementary Figure 2. Histology of cardiac tissues used in this study. Overview of representative slides stained with hematoxylin-eosin from cardiac samples in control and patient groups are shown. Higher magnification showing normal myocardium in control (panel A) and patient myocardium with hypertrophy of cardiomyocytes and interstitial fibrosis in the patient sample (panel B, both × 40 magnification). [file 13148_2020_895_MOESM2_ESM.tif]

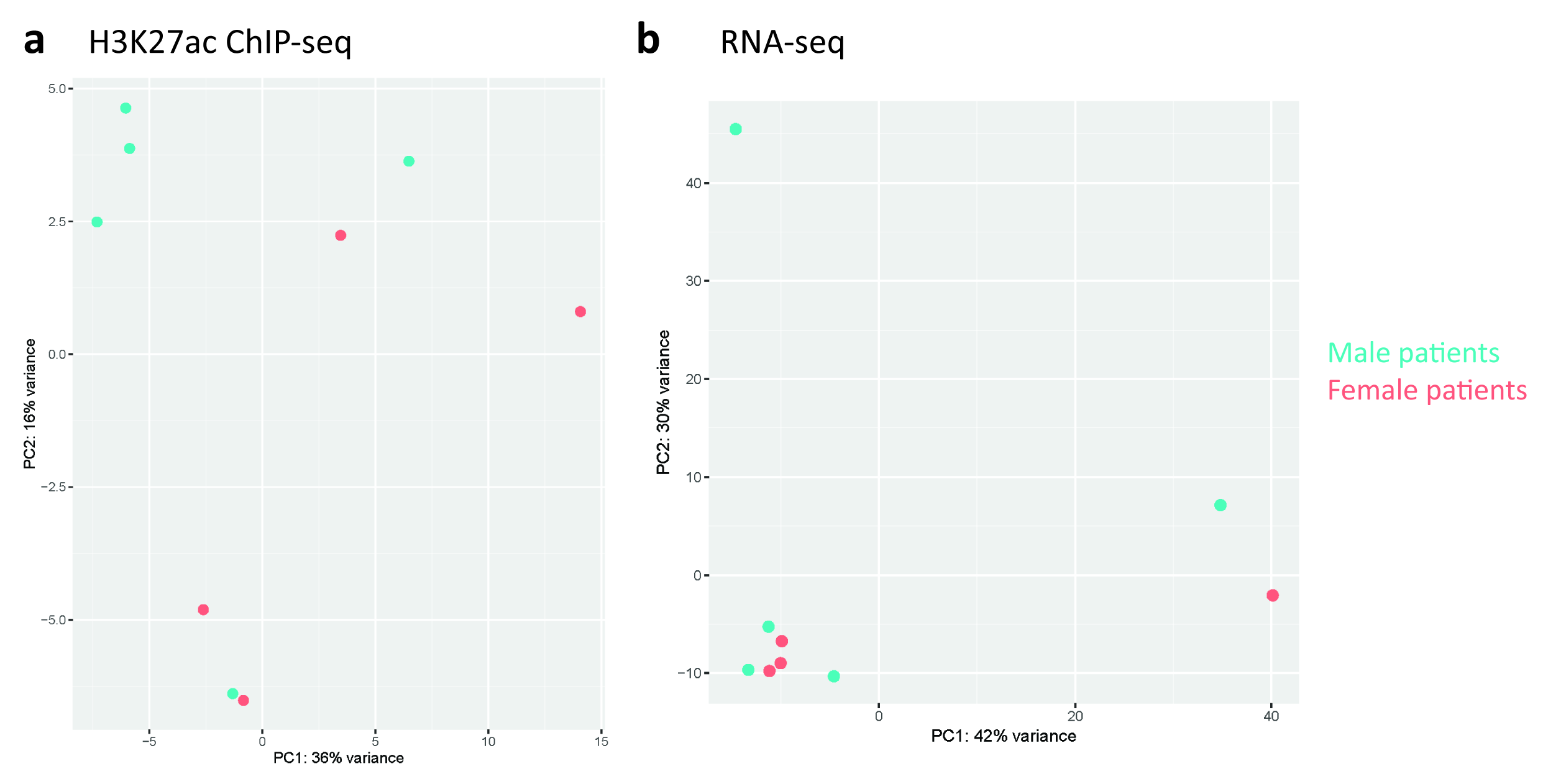

Supplement: Supplementary file 6 — Additional file 6. Supplementary Figure 6. Sex-specific H3K27ac acetylome and transcriptome profiles between male and female patients with concentric remodeling. a Principal component analysis (PCA) plot showing the clustering of man and woman cardiac samples based on H3K27ac profiles (using 500 regions with the highest variance). b PCA plot showing the clustering of man and woman cardiac samples based on the transcriptome profiles (using 500 genes with the highest variance). [file 13148_2020_895_MOESM6_ESM.tif]
