# Supplementary material for: H3K27ac acetylome signatures reveal the epigenomic reorganization in remodeled non-failing human hearts
Source: Clin Epigenetics. 2020 Jul 14;12:106. doi: 10.1186/s13148-020-00895-5 (PMC7362435; doi:10.1186/s13148-020-00895-5)

Cardiomyocytes

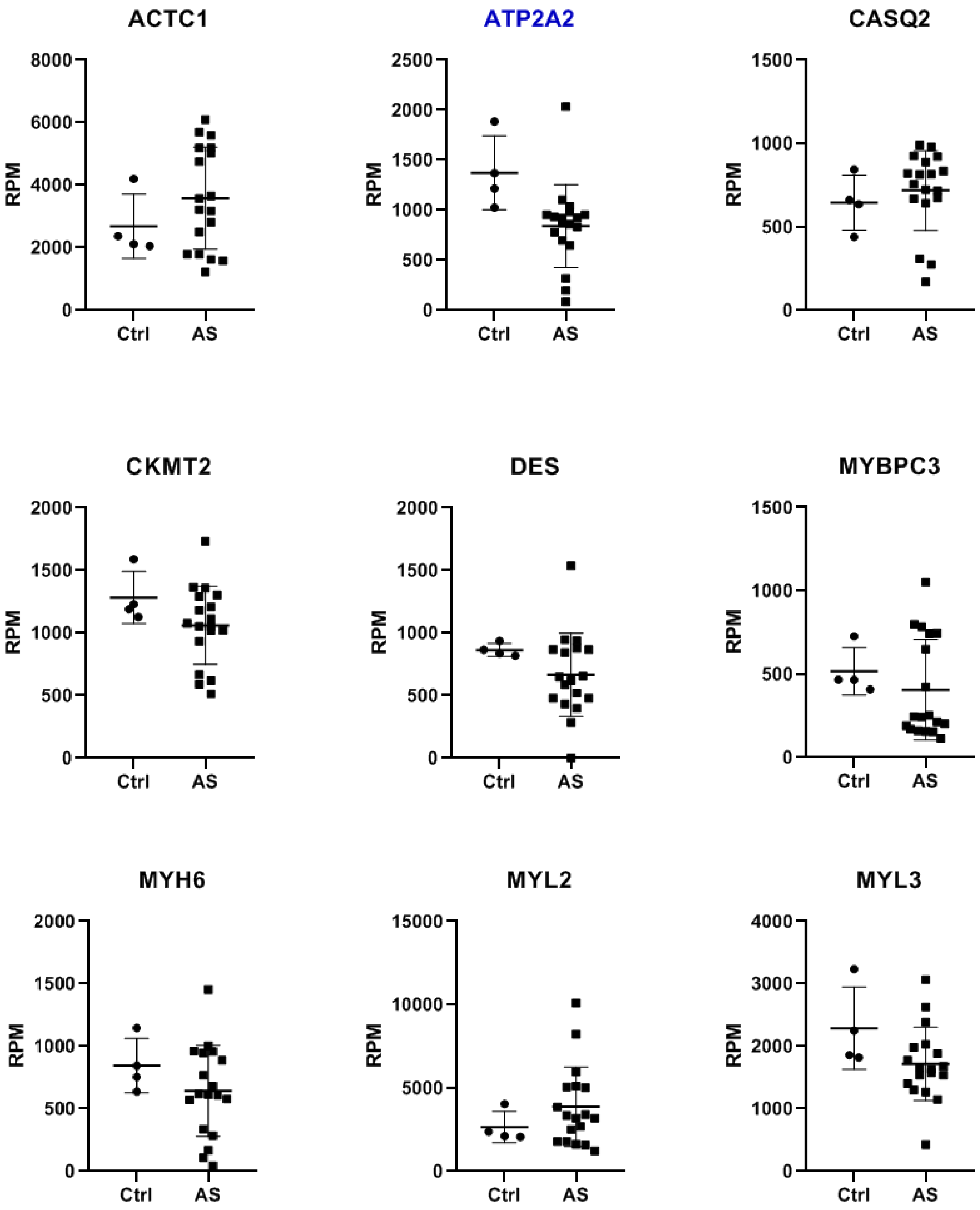

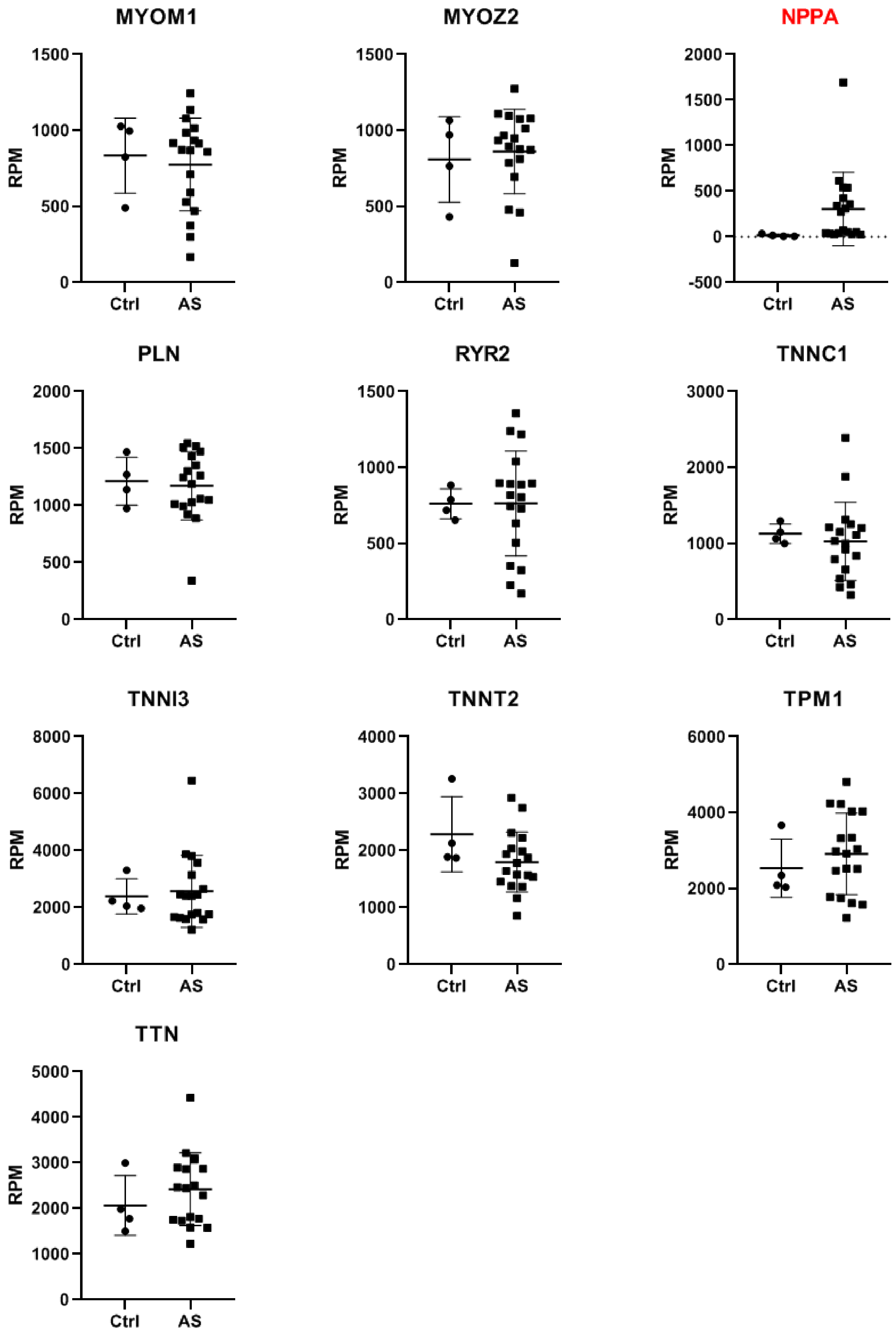
MYOM1
MYOZ2
NPPA
PLN
RYR2
TNNC1
TNNI3
TNNT2
TPM1
TTN
RPM
Ctrl
AS

Fibroblasts

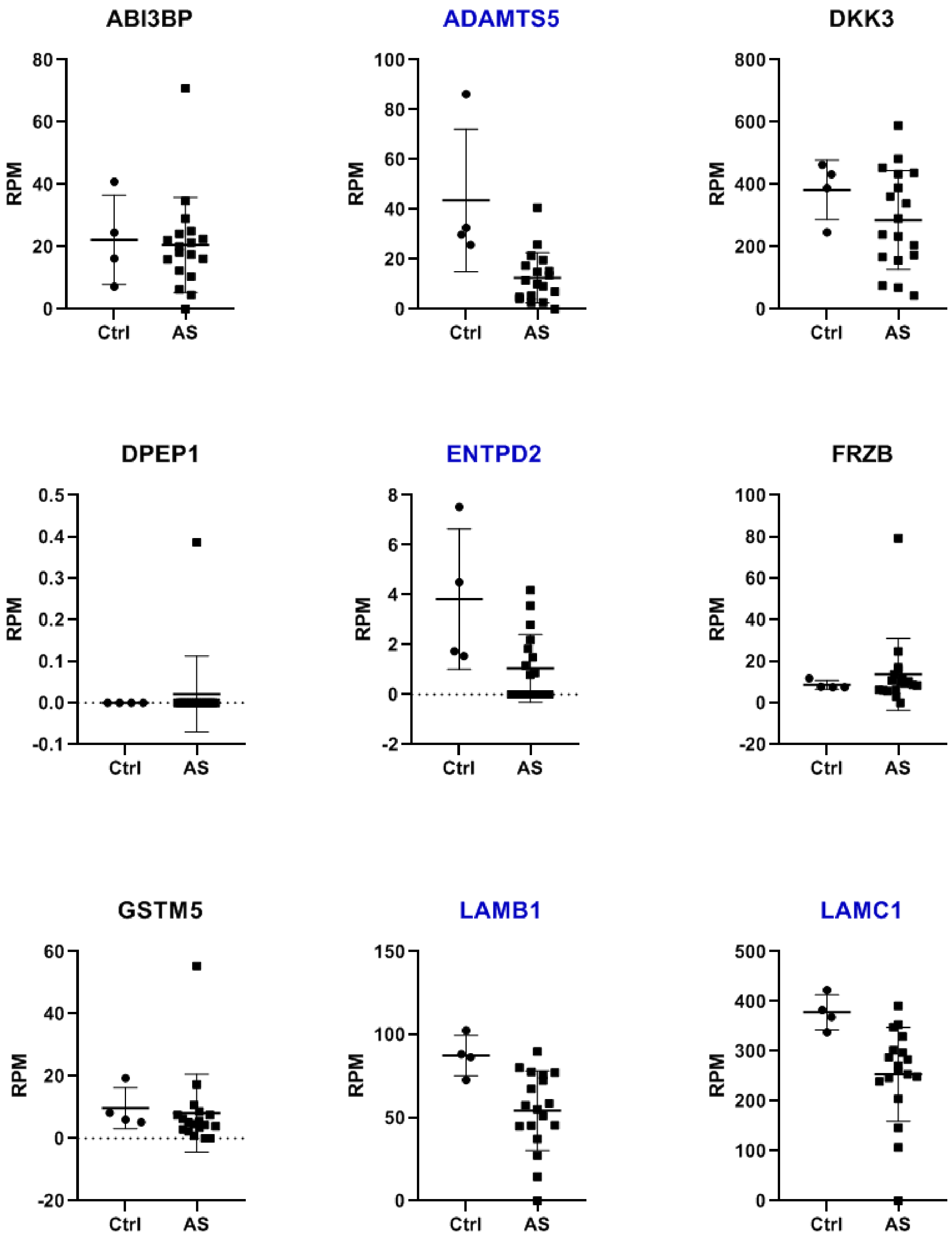

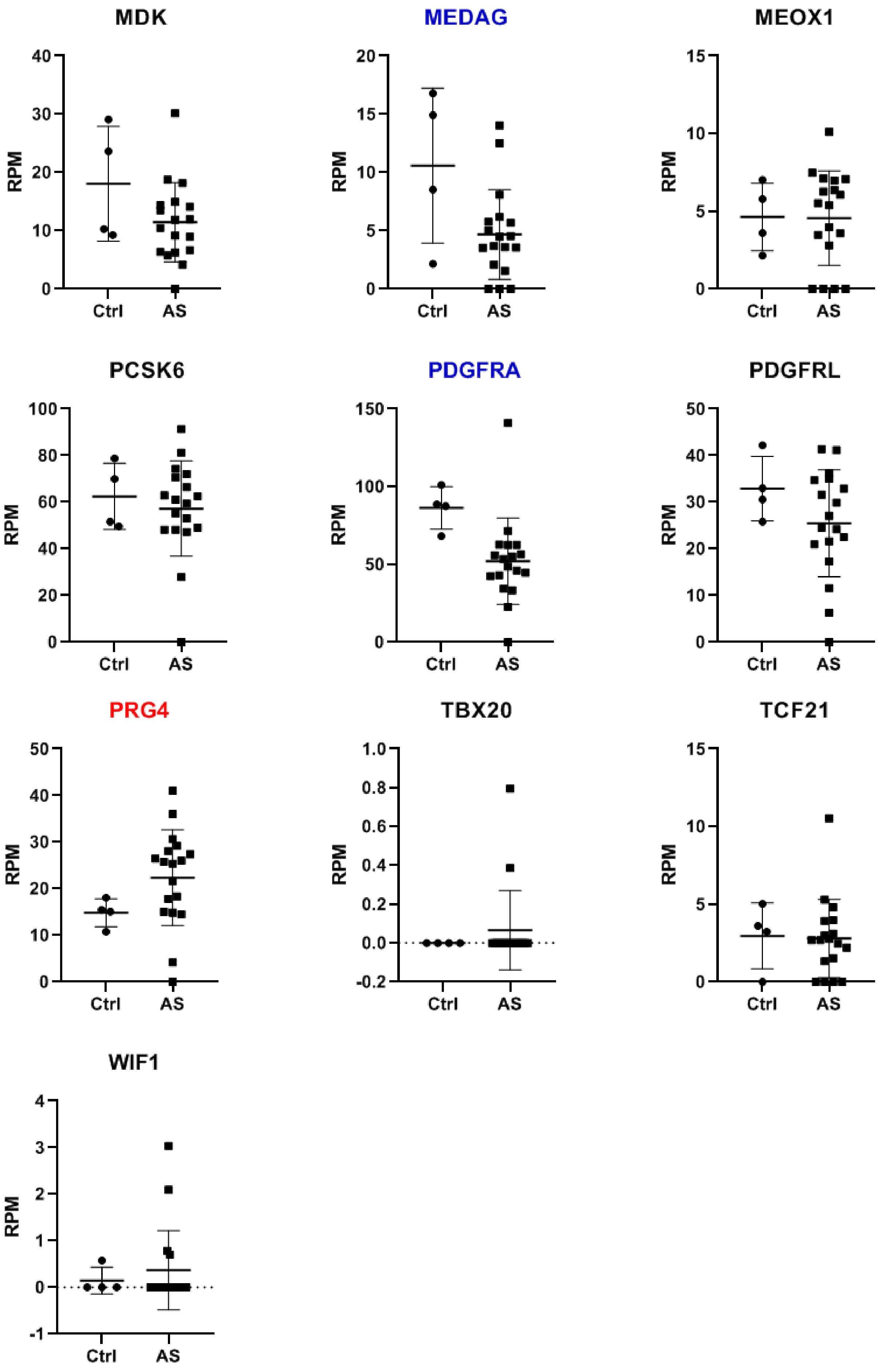
MDK
MEDAG
MEOX1
PCSK6
PDGFRA
PDGFRL
PRG4
TBX20
TCF21
WIF1
RPM
Ctrl
AS

Smooth muscle cells

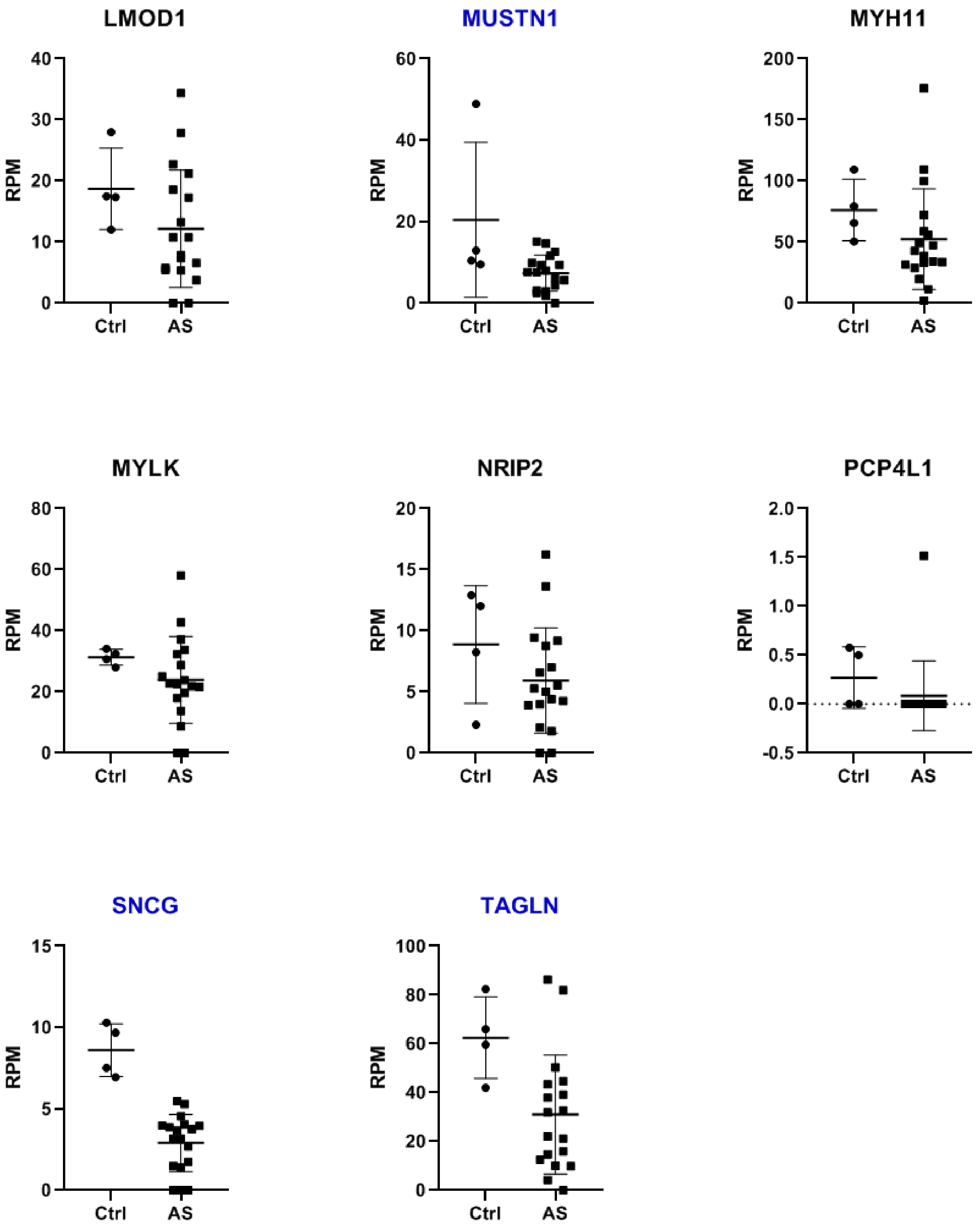

Endothelial cells

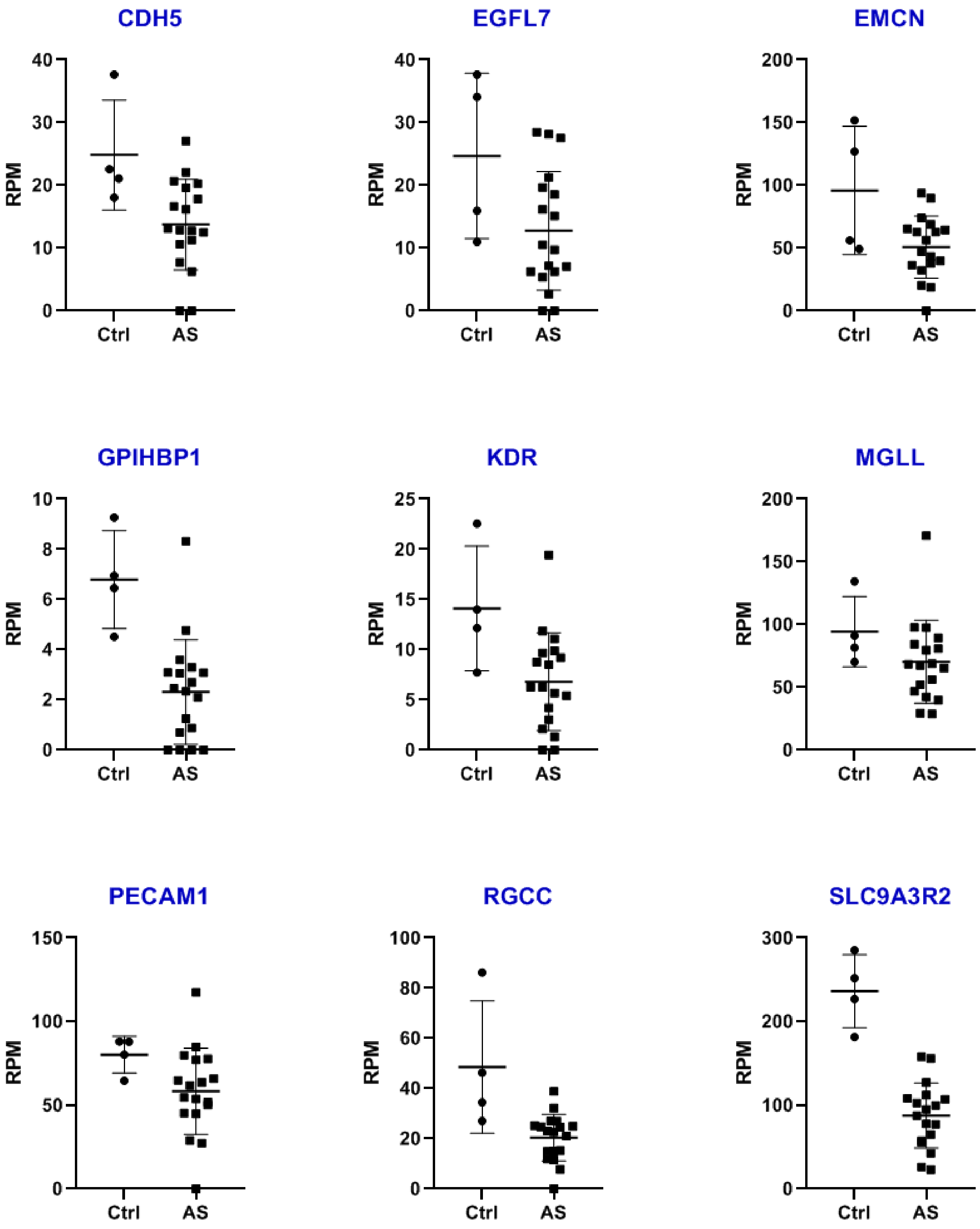

Pericytes

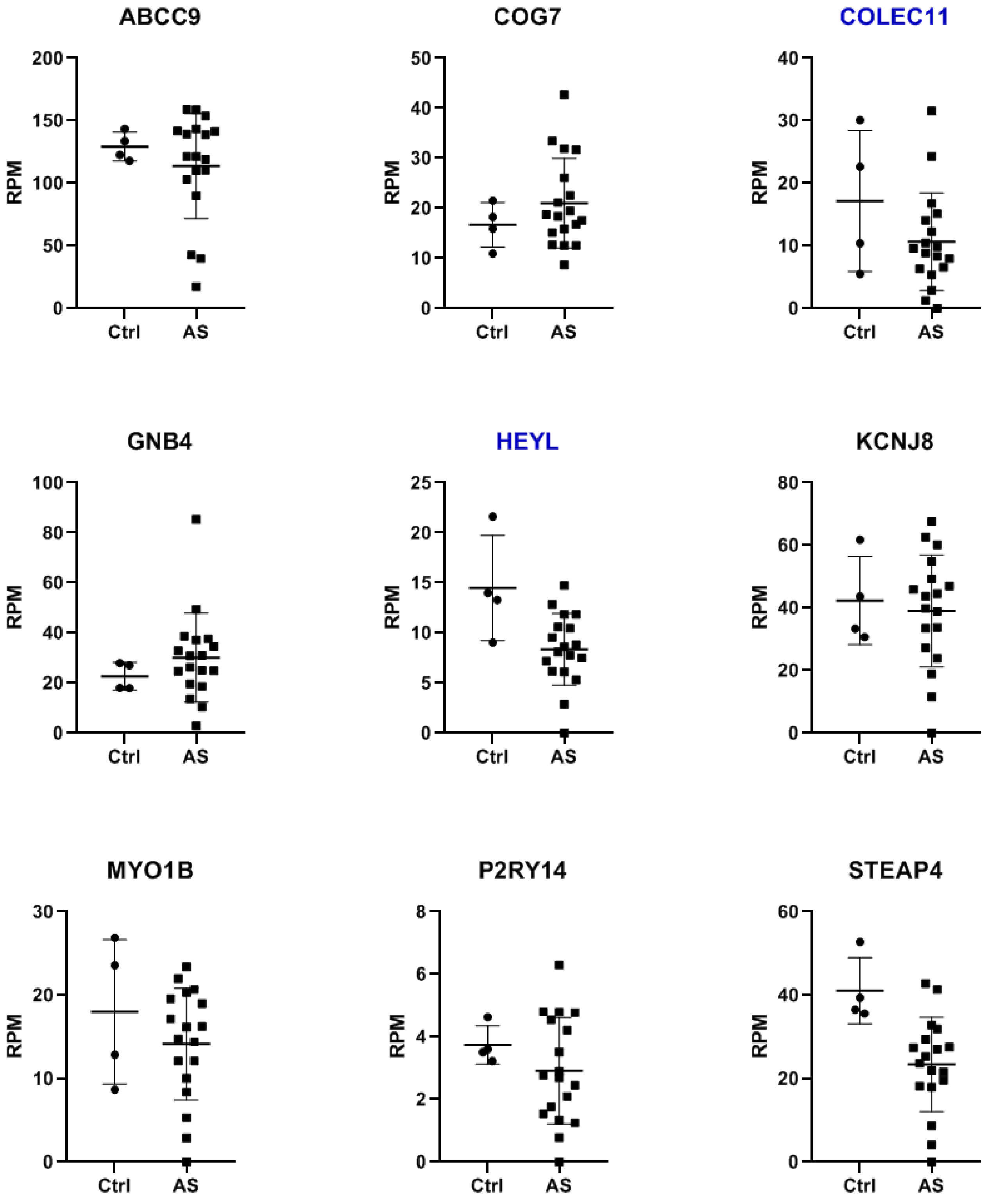

ABCC9
COG7
COLEC11
GNB4
HEYL
KCNJ8
MYO1B
P2RY14
STEAP4
RPM
Ctrl
AS

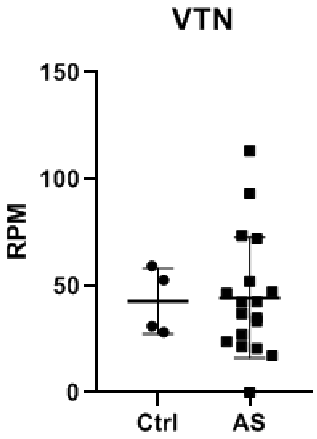

VTN
150
100
50
0
RPM
Ctrl
AS

Macrophages

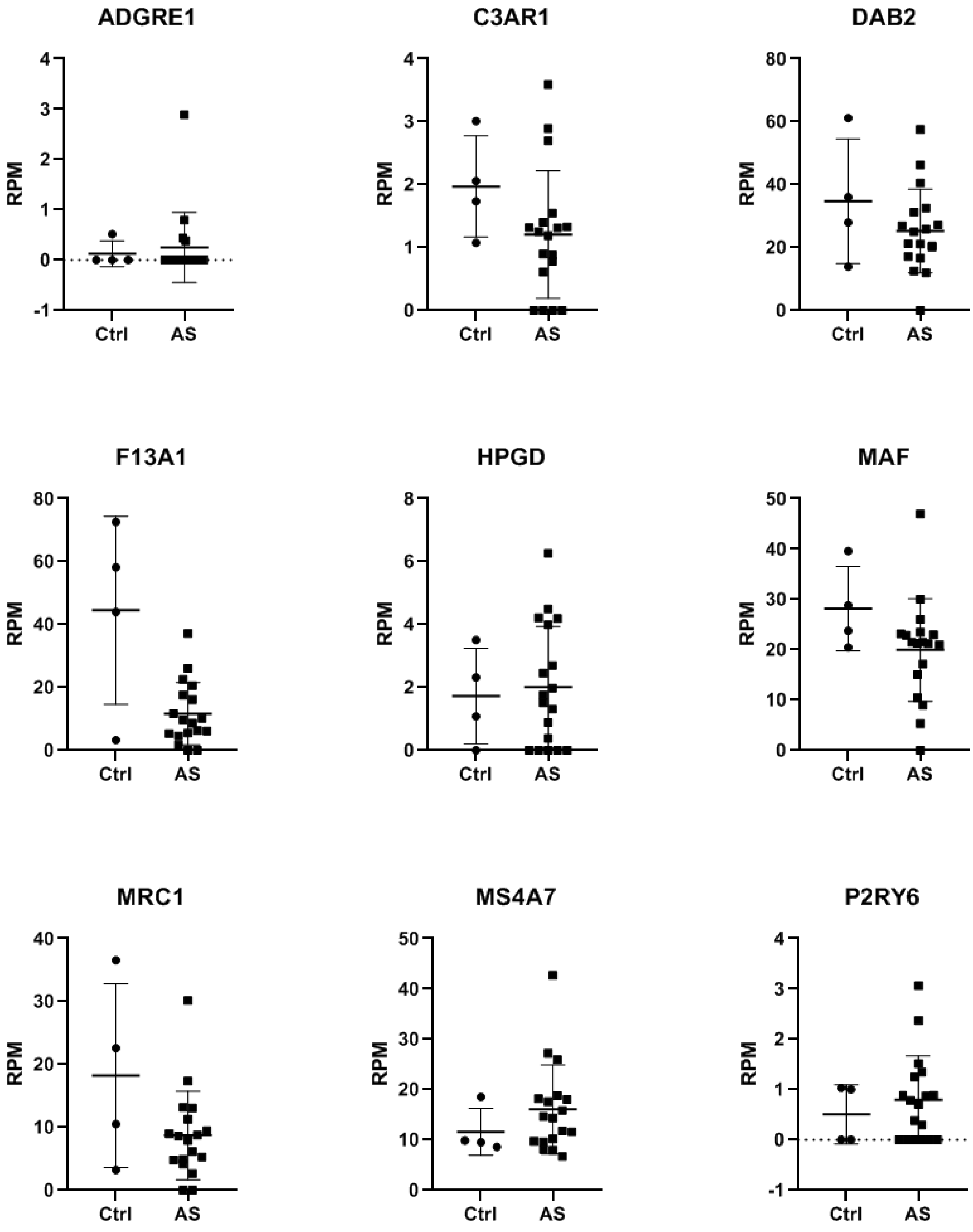

T cells

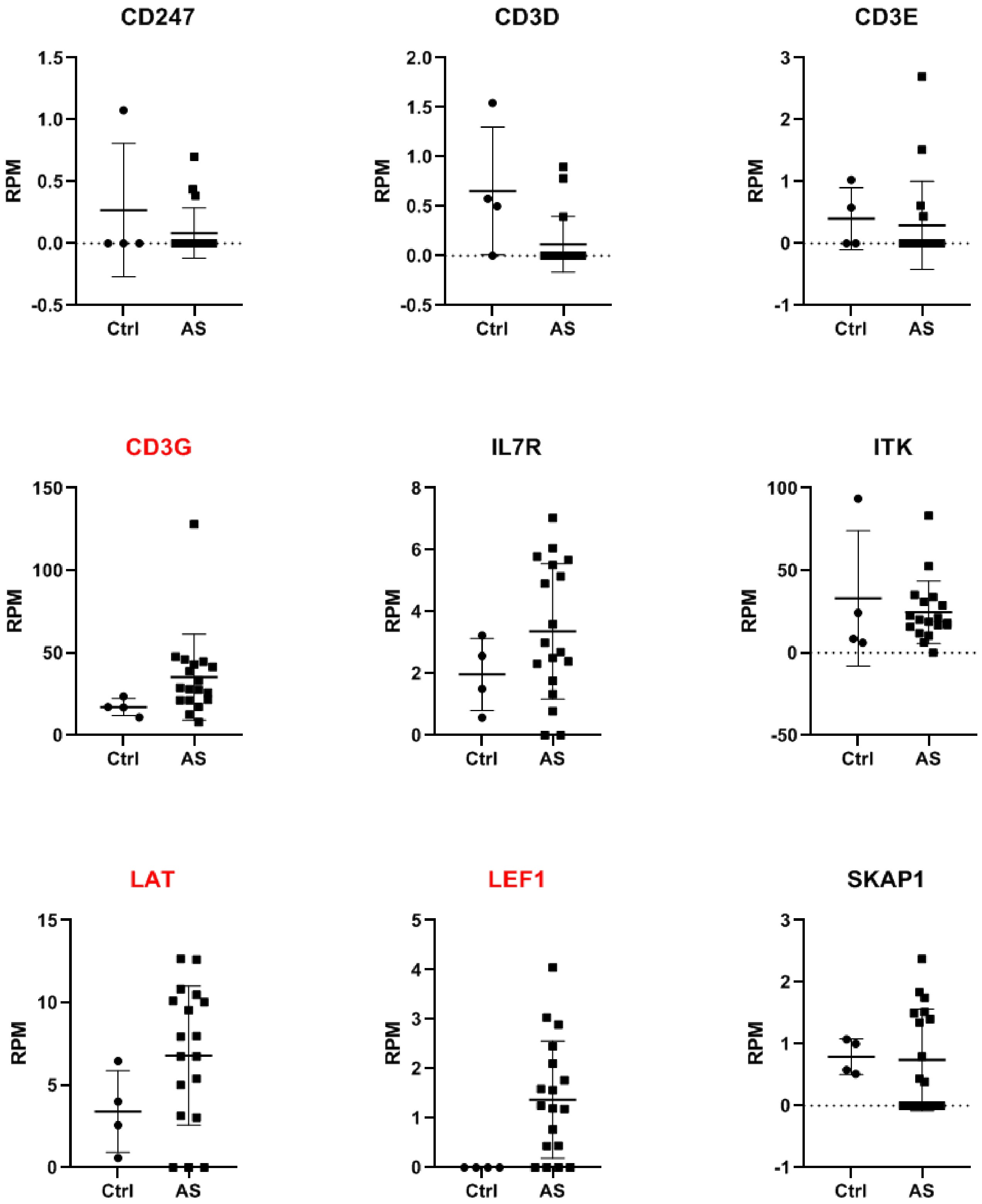
CD247
1.5
1.0
0.5
0.0
-0.5
RPM
Ctrl
AS
CD3D
2.0
1.5
1.0
0.5
0.0
-0.5
RPM
Ctrl
AS
CD3E
3
2
1
0
-1
RPM
Ctrl
AS
CD3G
150
100
50
0
RPM
Ctrl
AS
IL7R
8
6
4
2
0
RPM
Ctrl
AS
ITK
100
50
0
-50
RPM
Ctrl
AS
LAT
15
10
5
0
RPM
Ctrl
AS
LEF1
5
4
3
2
1
0
RPM
Ctrl
AS
SKAP1
3
2
1
0
-1
RPM
Ctrl
AS

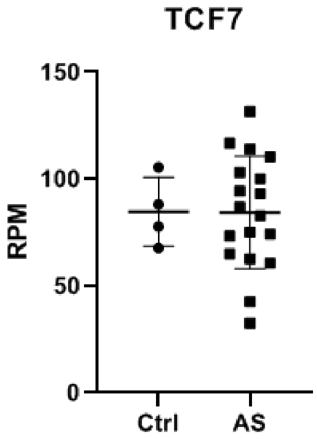
TCF7
RPM
150
100
50
0
Ctrl
AS

B cells

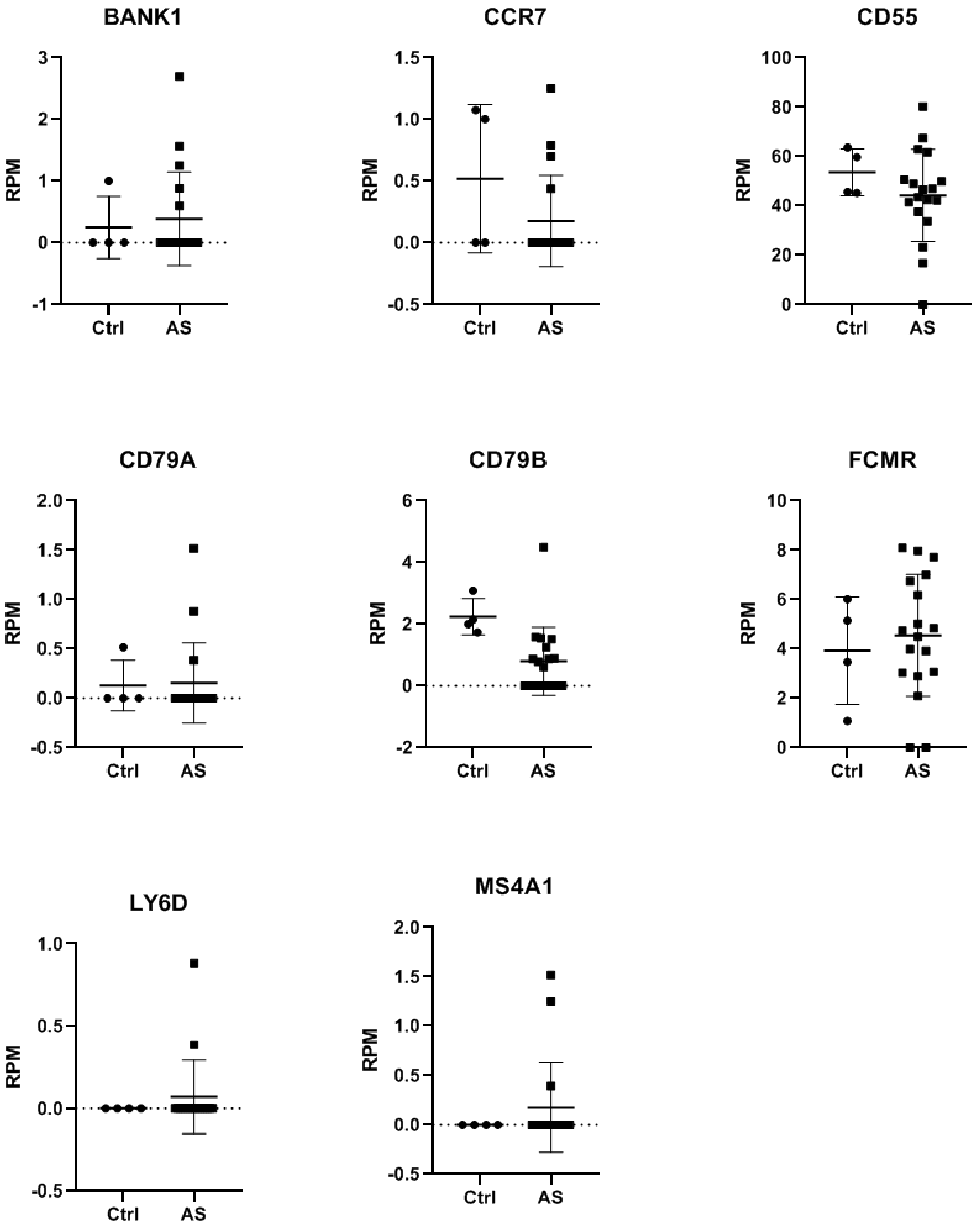
BANK1
3
2
1
0
-1
RPM
Ctrl
AS
CCR7
1.5
1.0
0.5
0.0
-0.5
RPM
Ctrl
AS
CD55
100
80
60
40
20
0
RPM
Ctrl
AS
CD79A
2.0
1.5
1.0
0.5
0.0
-0.5
RPM
Ctrl
AS
CD79B
6
4
2
0
-2
RPM
Ctrl
AS
FCMR
10
8
6
4
2
0
RPM
Ctrl
AS
LY6D
1.0
0.5
0.0
-0.5
RPM
Ctrl
AS
MS4A1
2.0
1.5
1.0
0.5
0.0
-0.5
RPM
Ctrl
AS

NK cells

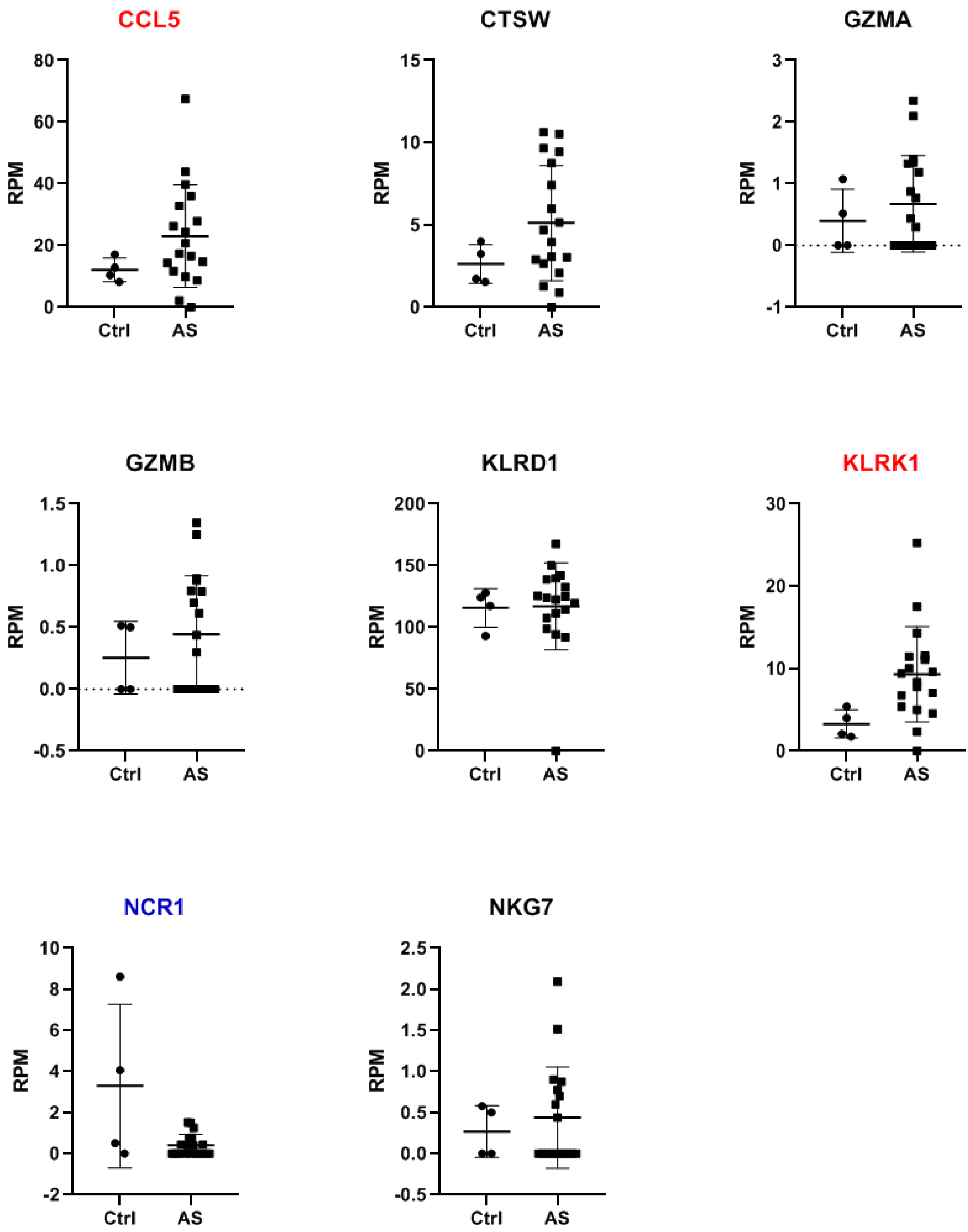

DC-like cells

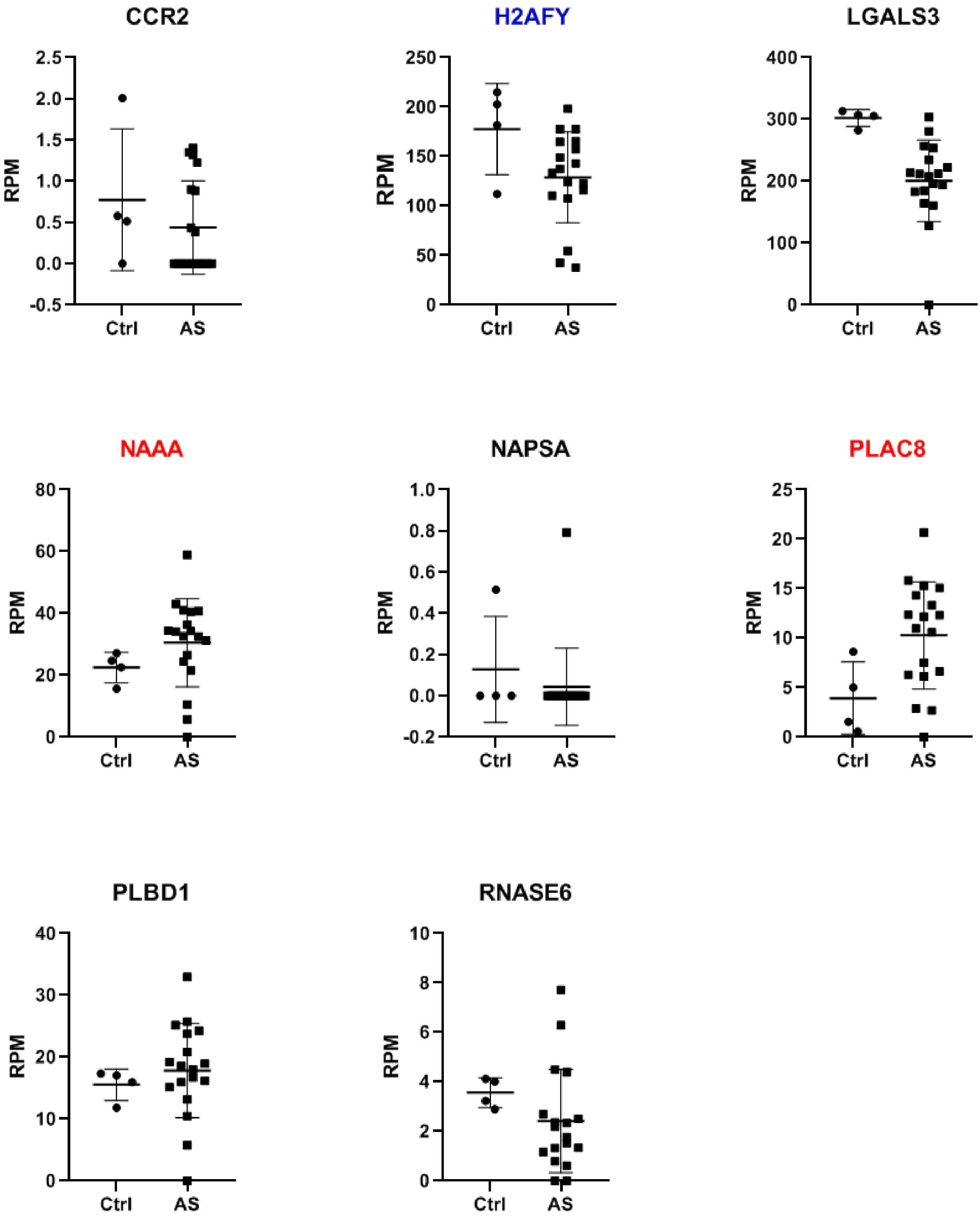

Granulocytes

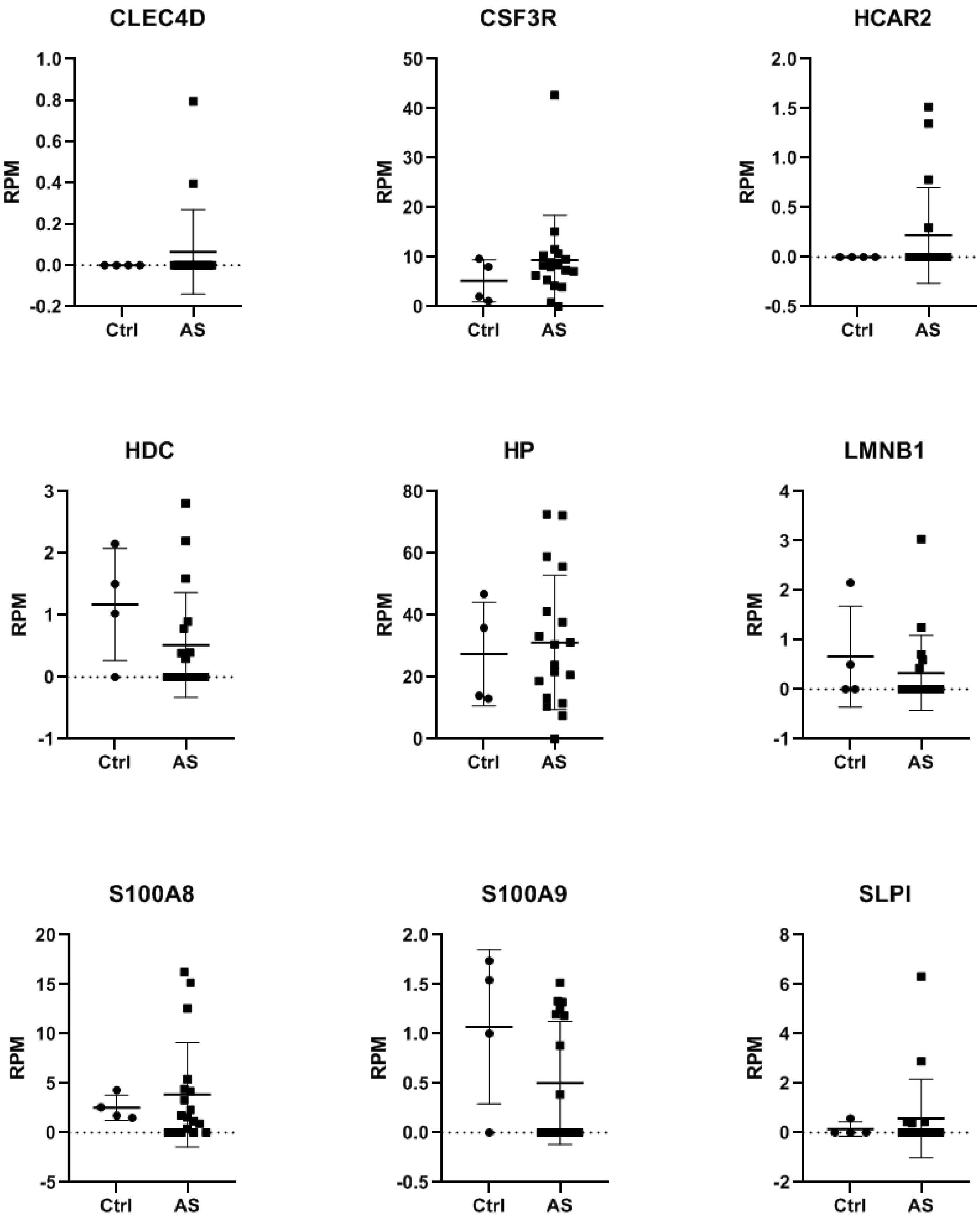

Schwann cells

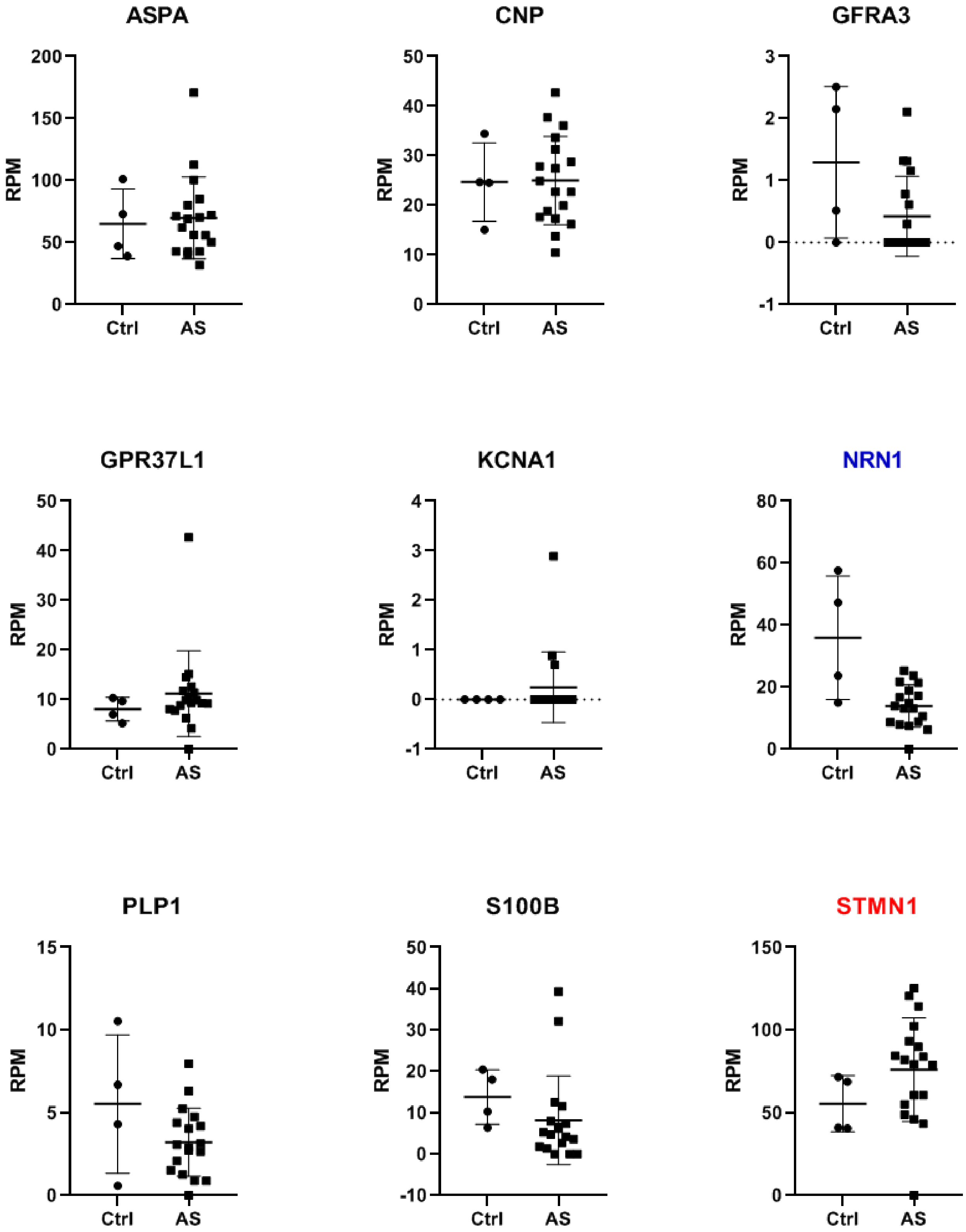

Supplement: Supplementary file 4 — Additional file 4 Supplementary Figure 4. The mRNA expression level of cell-type-specific markers. Genes labeled red and blue were significantly up- and downregulated in patients versus controls (p < 0.05). [file 13148_2020_895_MOESM4_ESM.pdf]
