# Supplementary material for: H3K27ac acetylome signatures reveal the epigenomic reorganization in remodeled non-failing human hearts
Source: Clin Epigenetics. 2020 Jul 14;12:106. doi: 10.1186/s13148-020-00895-5 (PMC7362435; doi:10.1186/s13148-020-00895-5)
Supplement: Supplementary file 5 — Additional file 5. Supplementary Figure 5. Gene Ontology (GO) analysis of genes annotated to DARs using ToppFun. a Molecular function enrichment for hyperacetylated regions, b Biological process for hyperacetylated regions, c Cellular component for hyperacetylated regions, d Molecular function for hypoacetylated regions, e Biological process for hypoacetylated regions, f Cellular component for hypoacetylated regions. [file 13148_2020_895_MOESM5_ESM.pdf]

Supplementary Figure 5A
Hyperacetylated genes

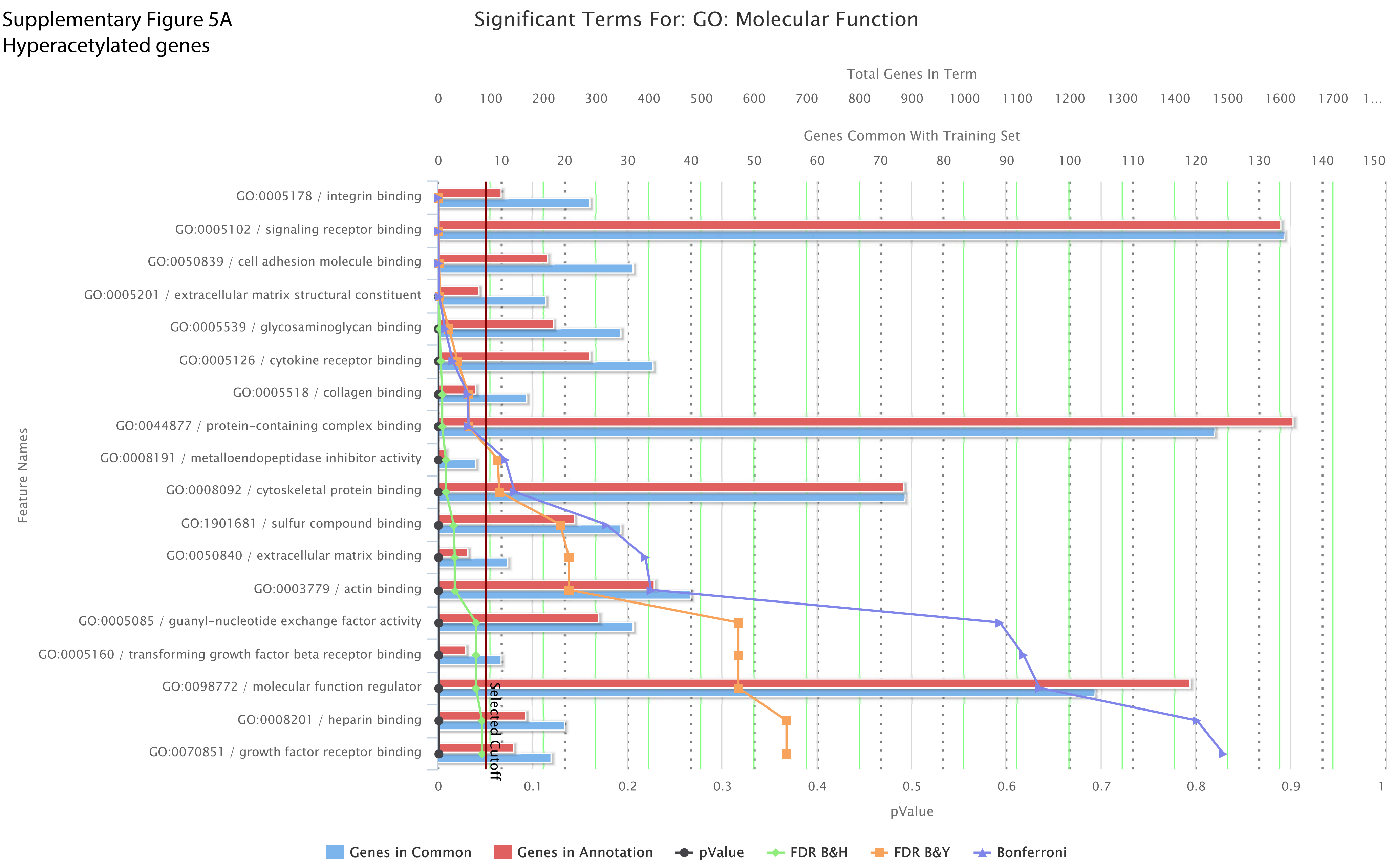

Supplementary Figure 5B
Hyperacetylated genes

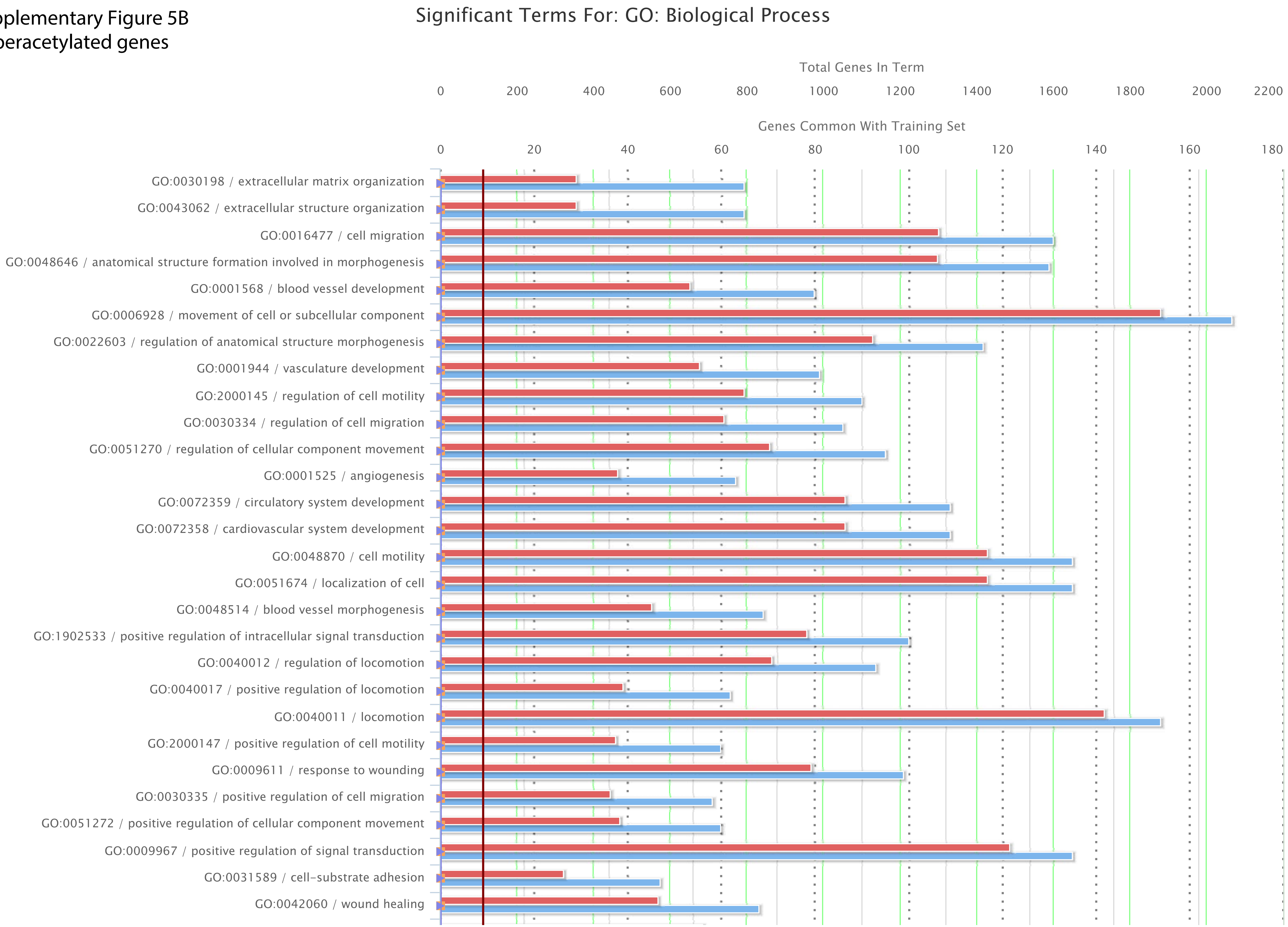

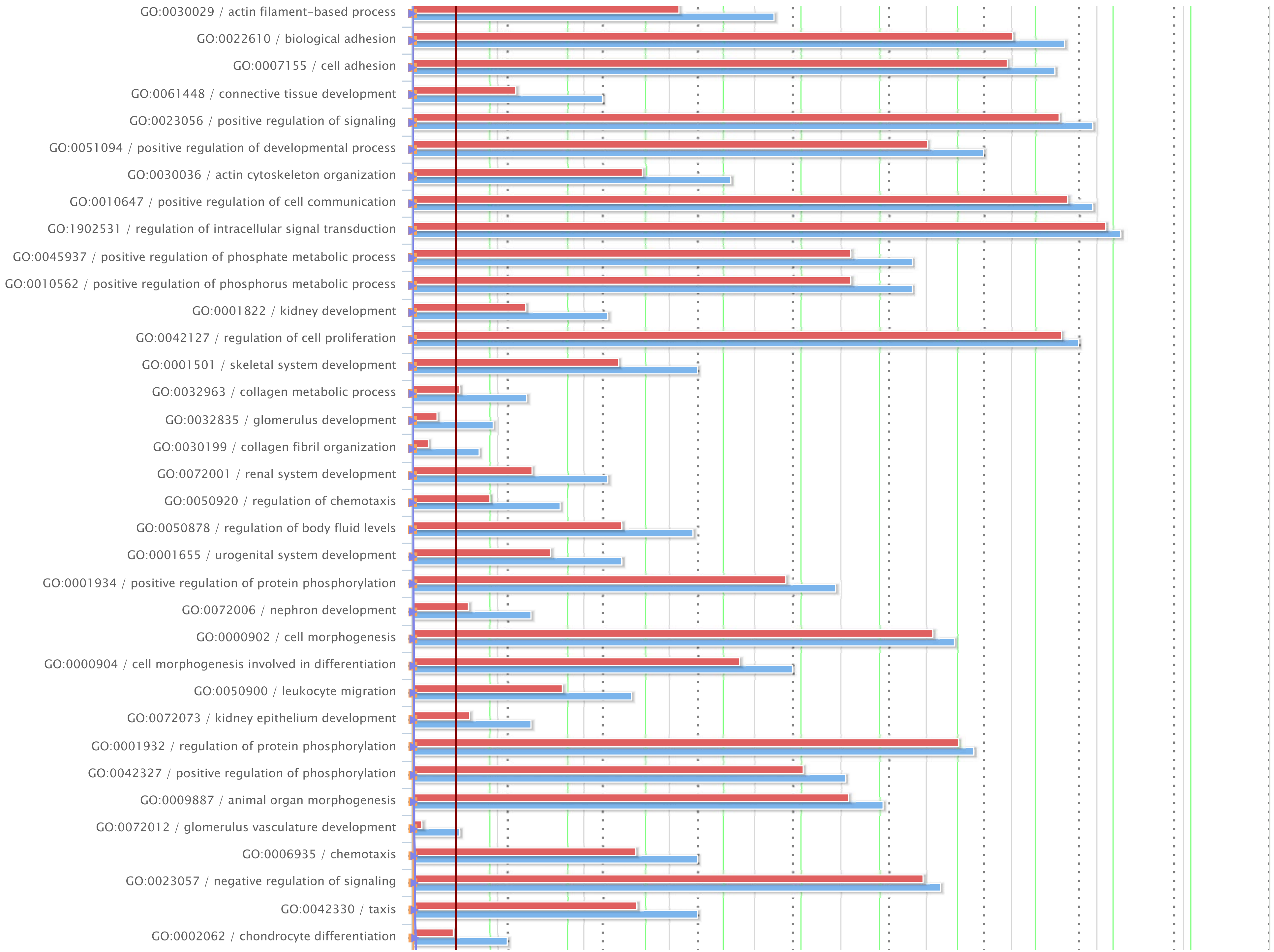
GO:0030029 / actin filament-based process
GO:0022610 / biological adhesion
GO:0007155 / cell adhesion
GO:0061448 / connective tissue development
GO:0023056 / positive regulation of signaling
GO:0051094 / positive regulation of developmental process
GO:0030036 / actin cytoskeleton organization
GO:0010647 / positive regulation of cell communication
GO:1902531 / regulation of intracellular signal transduction
GO:0045937 / positive regulation of phosphate metabolic process
GO:0010562 / positive regulation of phosphorus metabolic process
GO:0001822 / kidney development
GO:0042127 / regulation of cell proliferation
GO:0001501 / skeletal system development
GO:0032963 / collagen metabolic process
GO:0032835 / glomerulus development
GO:0030199 / collagen fibril organization
GO:0072001 / renal system development
GO:0050920 / regulation of chemotaxis
GO:0050878 / regulation of body fluid levels
GO:0001655 / urogenital system development
GO:0001934 / positive regulation of protein phosphorylation
GO:0072006 / nephron development
GO:0000902 / cell morphogenesis
GO:0000904 / cell morphogenesis involved in differentiation
GO:0050900 / leukocyte migration
GO:0072073 / kidney epithelium development
GO:0001932 / regulation of protein phosphorylation
GO:0042327 / positive regulation of phosphorylation
GO:0009887 / animal organ morphogenesis
GO:0072012 / glomerulus vasculature development
GO:0006935 / chemotaxis
GO:0023057 / negative regulation of signaling
GO:0042330 / taxis
GO:0002062 / chondrocyte differentiation

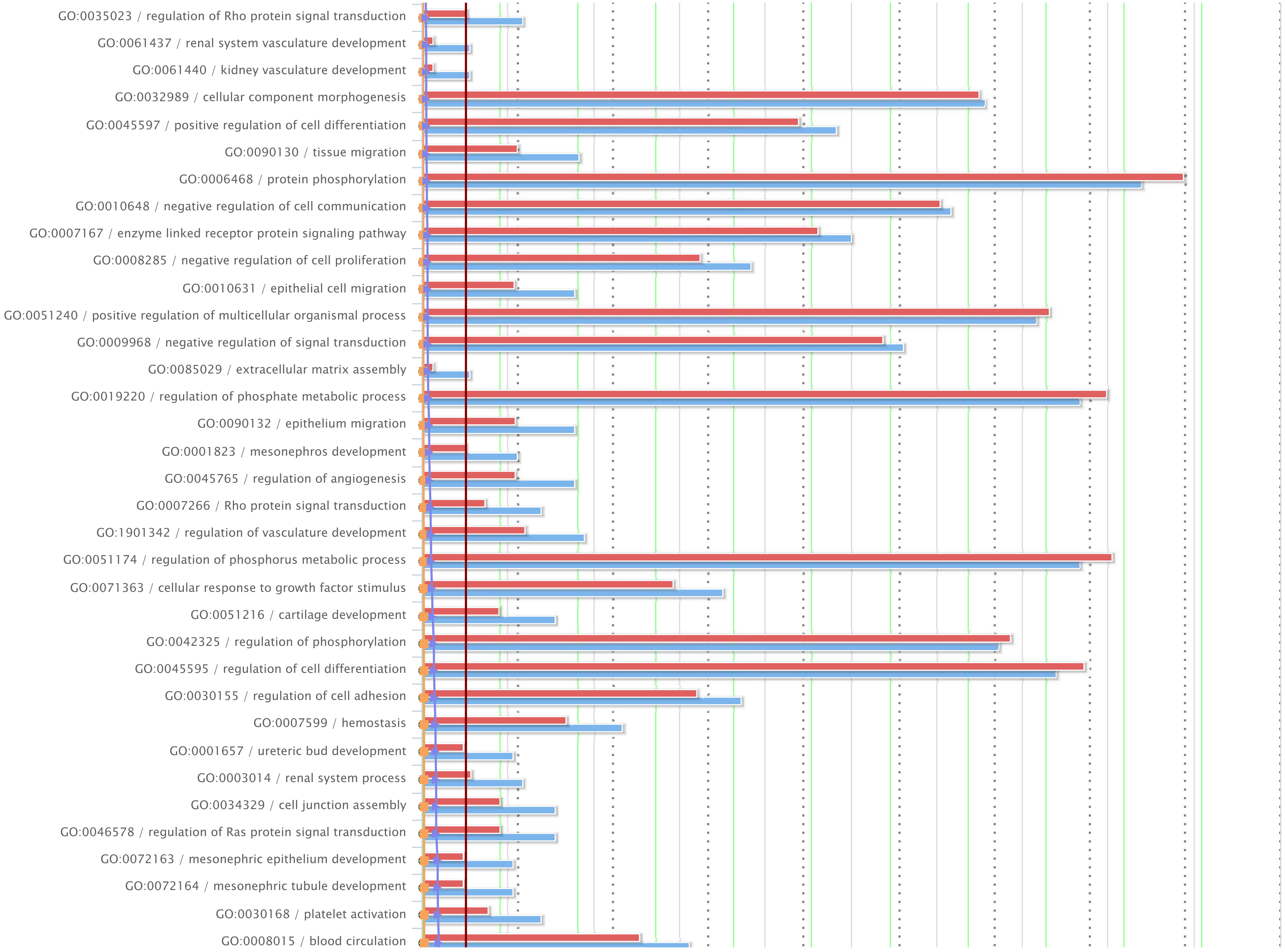
GO:0035023 / regulation of Rho protein signal transduction
GO:0061437 / renal system vasculature development
GO:0061440 / kidney vasculature development
GO:0032989 / cellular component morphogenesis
GO:0045597 / positive regulation of cell differentiation
GO:0090130 / tissue migration
GO:0006468 / protein phosphorylation
GO:0010648 / negative regulation of cell communication
GO:0007167 / enzyme linked receptor protein signaling pathway
GO:0008285 / negative regulation of cell proliferation
GO:0010631 / epithelial cell migration
GO:0051240 / positive regulation of multicellular organismal process
GO:0009968 / negative regulation of signal transduction
GO:0085029 / extracellular matrix assembly
GO:0019220 / regulation of phosphate metabolic process
GO:0090132 / epithelium migration
GO:0001823 / mesonephros development
GO:0045765 / regulation of angiogenesis
GO:0007266 / Rho protein signal transduction
GO:1901342 / regulation of vasculature development
GO:0051174 / regulation of phosphorus metabolic process
GO:0071363 / cellular response to growth factor stimulus
GO:0051216 / cartilage development
GO:0042325 / regulation of phosphorylation
GO:0045595 / regulation of cell differentiation
GO:0030155 / regulation of cell adhesion
GO:0007599 / hemostasis
GO:0001657 / ureteric bud development
GO:0003014 / renal system process
GO:0034329 / cell junction assembly
GO:0046578 / regulation of Ras protein signal transduction
GO:0072163 / mesonephric epithelium development
GO:0072164 / mesonephric tubule development
GO:0030168 / platelet activation
GO:0008015 / blood circulation

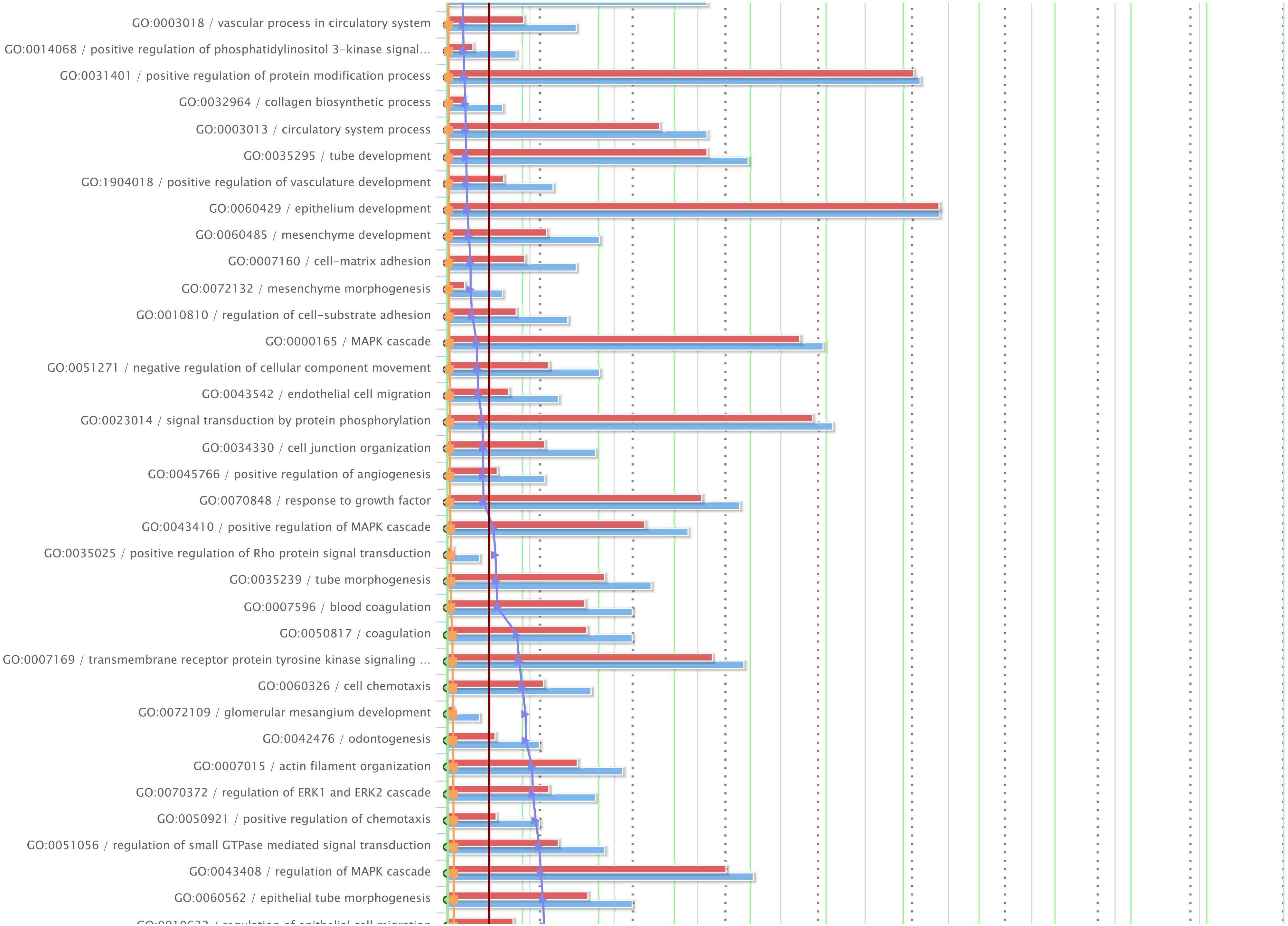
GO:0003018 / vascular process in circulatory system
GO:0014068 / positive regulation of phosphatidylinositol 3-kinase signal...
GO:0031401 / positive regulation of protein modification process
GO:0032964 / collagen biosynthetic process
GO:0003013 / circulatory system process
GO:0035295 / tube development
GO:1904018 / positive regulation of vasculature development
GO:0060429 / epithelium development
GO:0060485 / mesenchyme development
GO:0007160 / cell−matrix adhesion
GO:0072132 / mesenchyme morphogenesis
GO:0010810 / regulation of cell−substrate adhesion
GO:0000165 / MAPK cascade
GO:0051271 / negative regulation of cellular component movement
GO:0043542 / endothelial cell migration
GO:0023014 / signal transduction by protein phosphorylation
GO:0034330 / cell junction organization
GO:0045766 / positive regulation of angiogenesis
GO:0070848 / response to growth factor
GO:0043410 / positive regulation of MAPK cascade
GO:0035025 / positive regulation of Rho protein signal transduction
GO:0035239 / tube morphogenesis
GO:0007596 / blood coagulation
GO:0050817 / coagulation
GO:0007169 / transmembrane receptor protein tyrosine kinase signaling ...
GO:0060326 / cell chemotaxis
GO:0072109 / glomerular mesangium development
GO:0042476 / odontogenesis
GO:0007015 / actin filament organization
GO:0070372 / regulation of ERK1 and ERK2 cascade
GO:0050921 / positive regulation of chemotaxis
GO:0051056 / regulation of small GTPase mediated signal transduction
GO:0043408 / regulation of MAPK cascade
GO:0060562 / epithelial tube morphogenesis

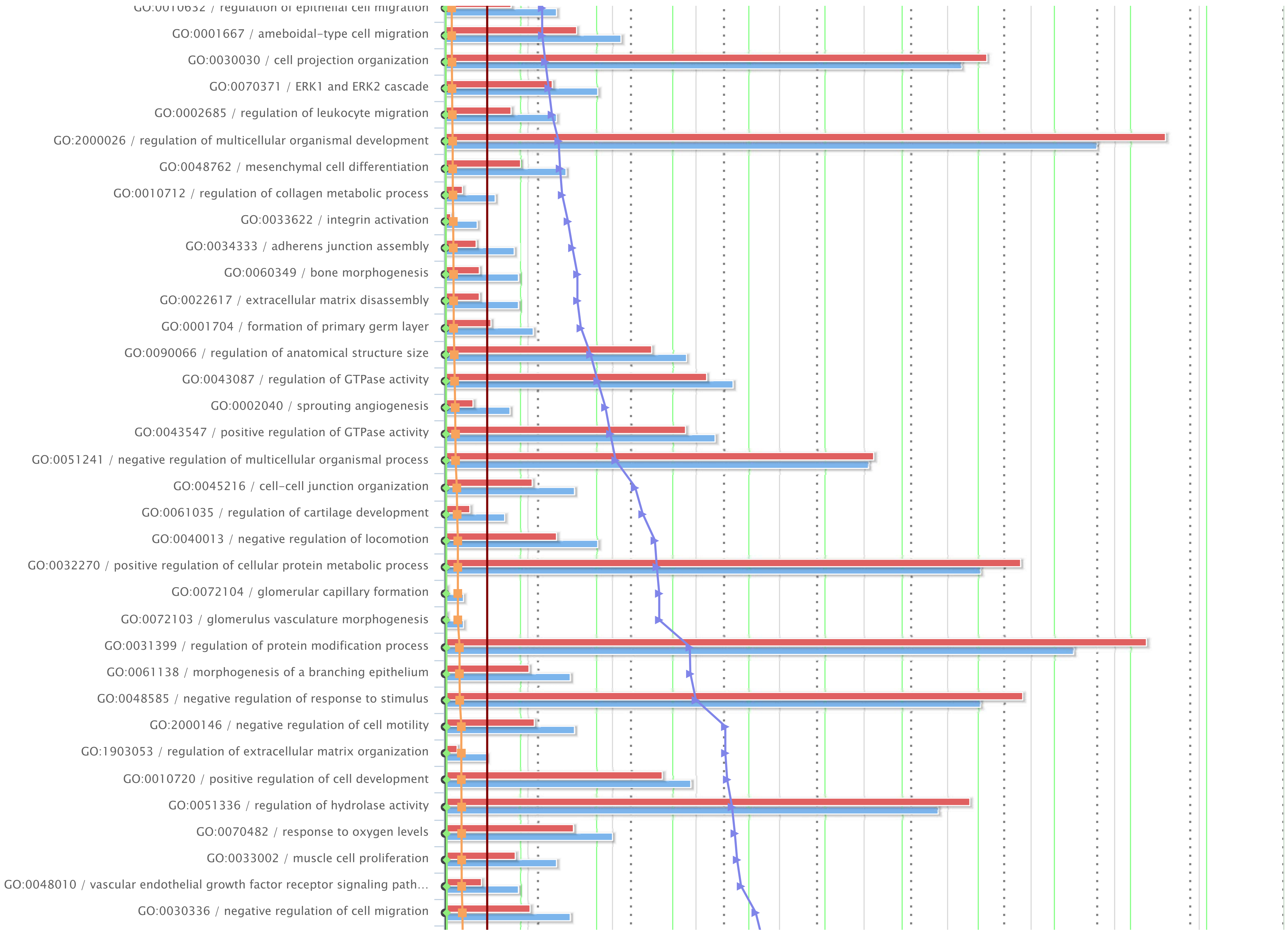
GO:0010632 / regulation of epithelial cell migration
GO:0001667 / ameboidal-type cell migration
GO:0030030 / cell projection organization
GO:0070371 / ERK1 and ERK2 cascade
GO:0002685 / regulation of leukocyte migration
GO:2000026 / regulation of multicellular organismal development
GO:0048762 / mesenchymal cell differentiation
GO:0010712 / regulation of collagen metabolic process
GO:0033622 / integrin activation
GO:0034333 / adherens junction assembly
GO:0060349 / bone morphogenesis
GO:0022617 / extracellular matrix disassembly
GO:0001704 / formation of primary germ layer
GO:0090066 / regulation of anatomical structure size
GO:0043087 / regulation of GTPase activity
GO:0002040 / sprouting angiogenesis
GO:0043547 / positive regulation of GTPase activity
GO:0051241 / negative regulation of multicellular organismal process
GO:0045216 / cell-cell junction organization
GO:0061035 / regulation of cartilage development
GO:0040013 / negative regulation of locomotion
GO:0032270 / positive regulation of cellular protein metabolic process
GO:0072104 / glomerular capillary formation
GO:0072103 / glomerulus vasculature morphogenesis
GO:0031399 / regulation of protein modification process
GO:0061138 / morphogenesis of a branching epithelium
GO:0048585 / negative regulation of response to stimulus
GO:2000146 / negative regulation of cell motility
GO:1903053 / regulation of extracellular matrix organization
GO:0010720 / positive regulation of cell development
GO:0051336 / regulation of hydrolase activity
GO:0070482 / response to oxygen levels
GO:0033002 / muscle cell proliferation
GO:0048010 / vascular endothelial growth factor receptor signaling path...
GO:0030336 / negative regulation of cell migration

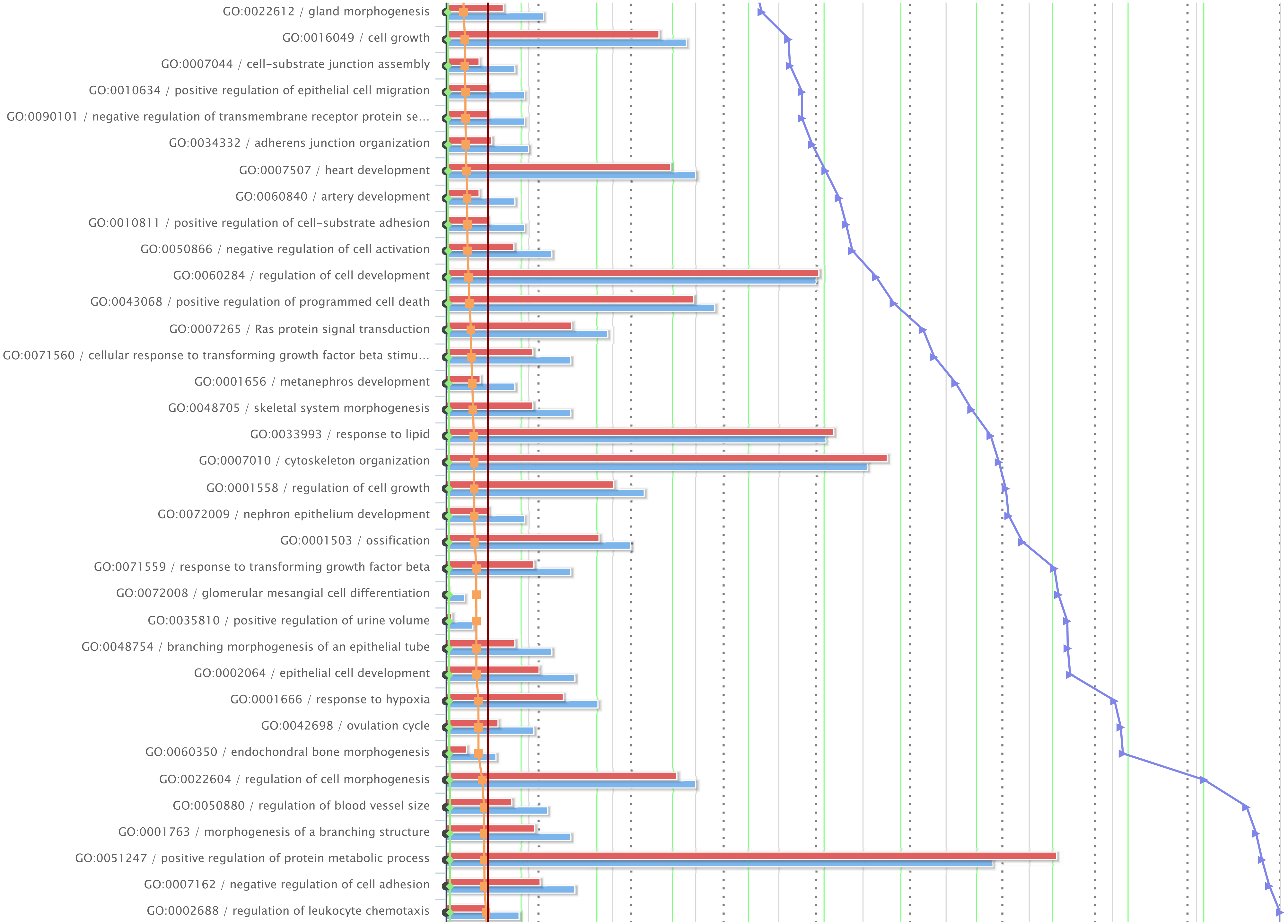

GO:0022612 / gland morphogenesis
GO:0016049 / cell growth
GO:0007044 / cell−substrate junction assembly
GO:0010634 / positive regulation of epithelial cell migration
GO:0090101 / negative regulation of transmembrane receptor protein se...
GO:0034332 / adherens junction organization
GO:0007507 / heart development
GO:0060840 / artery development
GO:0010811 / positive regulation of cell−substrate adhesion
GO:0050866 / negative regulation of cell activation
GO:0060284 / regulation of cell development
GO:0043068 / positive regulation of programmed cell death
GO:0007265 / Ras protein signal transduction
GO:0071560 / cellular response to transforming growth factor beta stimu...
GO:0001656 / metanephros development
GO:0048705 / skeletal system morphogenesis
GO:0033993 / response to lipid
GO:0007010 / cytoskeleton organization
GO:0001558 / regulation of cell growth
GO:0072009 / nephron epithelium development
GO:0001503 / ossification
GO:0071559 / response to transforming growth factor beta
GO:0072008 / glomerular mesangial cell differentiation
GO:0035810 / positive regulation of urine volume
GO:0048754 / branching morphogenesis of an epithelial tube
GO:0002064 / epithelial cell development
GO:0001666 / response to hypoxia
GO:0042698 / ovulation cycle
GO:0060350 / endochondral bone morphogenesis
GO:0022604 / regulation of cell morphogenesis
GO:0050880 / regulation of blood vessel size
GO:0001763 / morphogenesis of a branching structure
GO:0051247 / positive regulation of protein metabolic process
GO:0007162 / negative regulation of cell adhesion
GO:0002688 / regulation of leukocyte chemotaxis

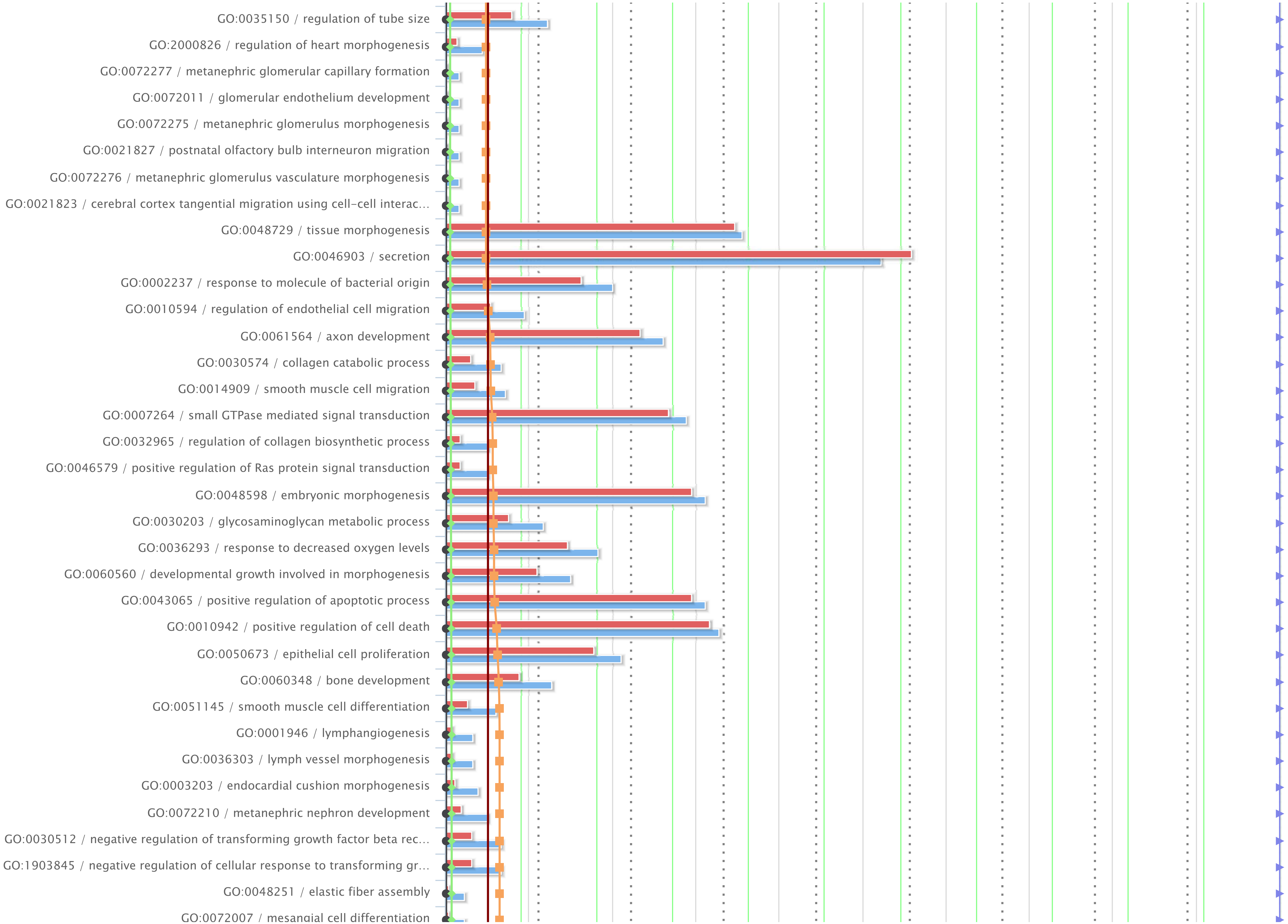
GO:0035150 / regulation of tube size
GO:2000826 / regulation of heart morphogenesis
GO:0072277 / metanephric glomerular capillary formation
GO:0072011 / glomerular endothelium development
GO:0072275 / metanephric glomerulus morphogenesis
GO:0021827 / postnatal olfactory bulb interneuron migration
GO:0072276 / metanephric glomerulus vasculature morphogenesis
GO:0021823 / cerebral cortex tangential migration using cell-cell interac...
GO:0048729 / tissue morphogenesis
GO:0046903 / secretion
GO:0002237 / response to molecule of bacterial origin
GO:0010594 / regulation of endothelial cell migration
GO:0061564 / axon development
GO:0030574 / collagen catabolic process
GO:0014909 / smooth muscle cell migration
GO:0007264 / small GTPase mediated signal transduction
GO:0032965 / regulation of collagen biosynthetic process
GO:0046579 / positive regulation of Ras protein signal transduction
GO:0048598 / embryonic morphogenesis
GO:0030203 / glycosaminoglycan metabolic process
GO:0036293 / response to decreased oxygen levels
GO:0060560 / developmental growth involved in morphogenesis
GO:0043065 / positive regulation of apoptotic process
GO:0010942 / positive regulation of cell death
GO:0050673 / epithelial cell proliferation
GO:0060348 / bone development
GO:0051145 / smooth muscle cell differentiation
GO:0001946 / lymphangiogenesis
GO:0036303 / lymph vessel morphogenesis
GO:0003203 / endocardial cushion morphogenesis
GO:0072210 / metanephric nephron development
GO:0030512 / negative regulation of transforming growth factor beta rec...
GO:1903845 / negative regulation of cellular response to transforming gr...
GO:0048251 / elastic fiber assembly
GO:0072007 / mesangial cell differentiation

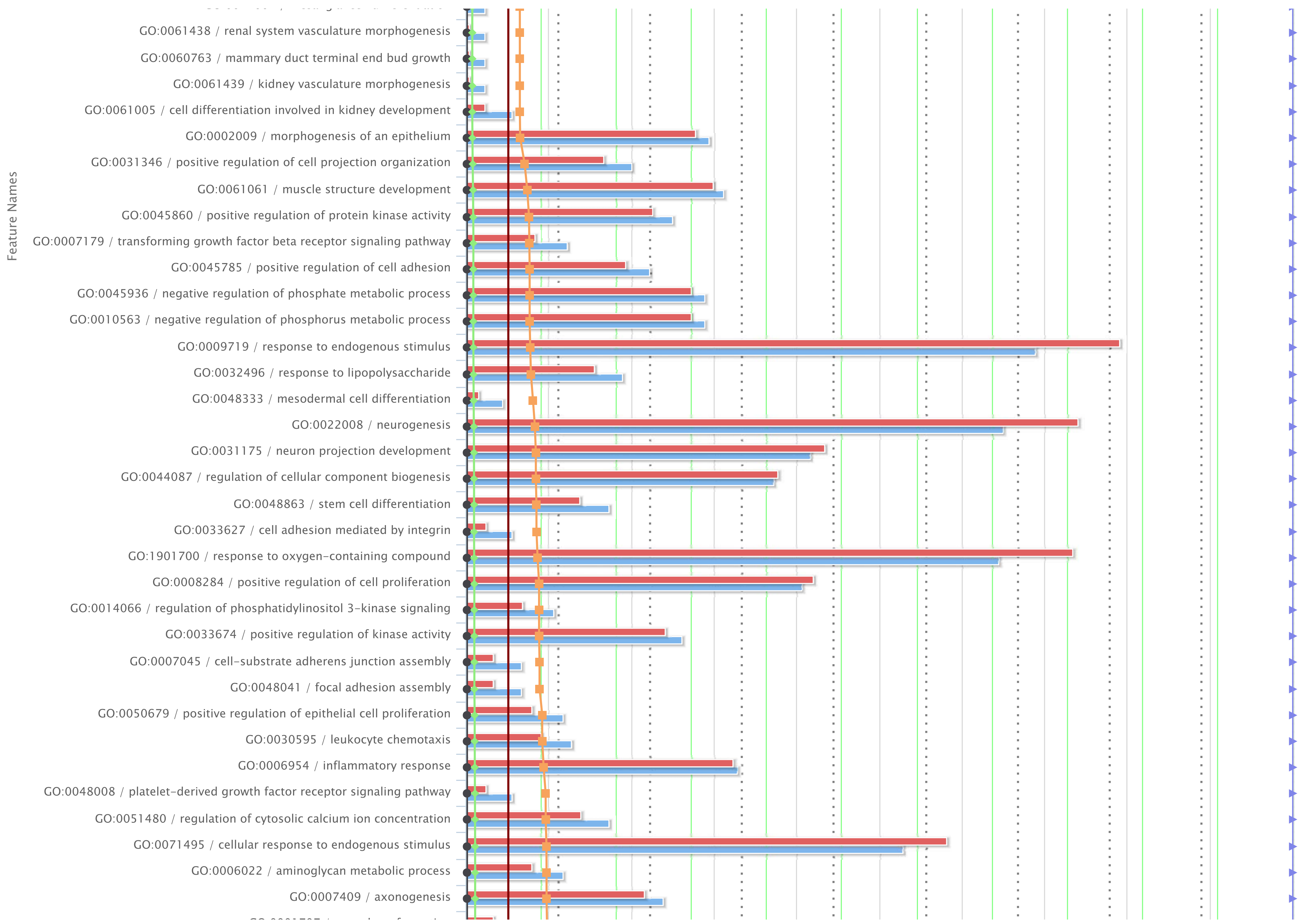

Feature Names
GO:0061438 / renal system vasculature morphogenesis
GO:0060763 / mammary duct terminal end bud growth
GO:0061439 / kidney vasculature morphogenesis
GO:0061005 / cell differentiation involved in kidney development
GO:0002009 / morphogenesis of an epithelium
GO:0031346 / positive regulation of cell projection organization
GO:0061061 / muscle structure development
GO:0045860 / positive regulation of protein kinase activity
GO:0007179 / transforming growth factor beta receptor signaling pathway
GO:0045785 / positive regulation of cell adhesion
GO:0045936 / negative regulation of phosphate metabolic process
GO:0010563 / negative regulation of phosphorus metabolic process
GO:0009719 / response to endogenous stimulus
GO:0032496 / response to lipopolysaccharide
GO:0048333 / mesodermal cell differentiation
GO:0022008 / neurogenesis
GO:0031175 / neuron projection development
GO:0044087 / regulation of cellular component biogenesis
GO:0048863 / stem cell differentiation
GO:0033627 / cell adhesion mediated by integrin
GO:1901700 / response to oxygen-containing compound
GO:0008284 / positive regulation of cell proliferation
GO:0014066 / regulation of phosphatidylinositol 3-kinase signaling
GO:0033674 / positive regulation of kinase activity
GO:0007045 / cell-substrate adherens junction assembly
GO:0048041 / focal adhesion assembly
GO:0050679 / positive regulation of epithelial cell proliferation
GO:0030595 / leukocyte chemotaxis
GO:0006954 / inflammatory response
GO:0048008 / platelet-derived growth factor receptor signaling pathway
GO:0051480 / regulation of cytosolic calcium ion concentration
GO:0071495 / cellular response to endogenous stimulus
GO:0006022 / aminoglycan metabolic process
GO:0007409 / axonogenesis

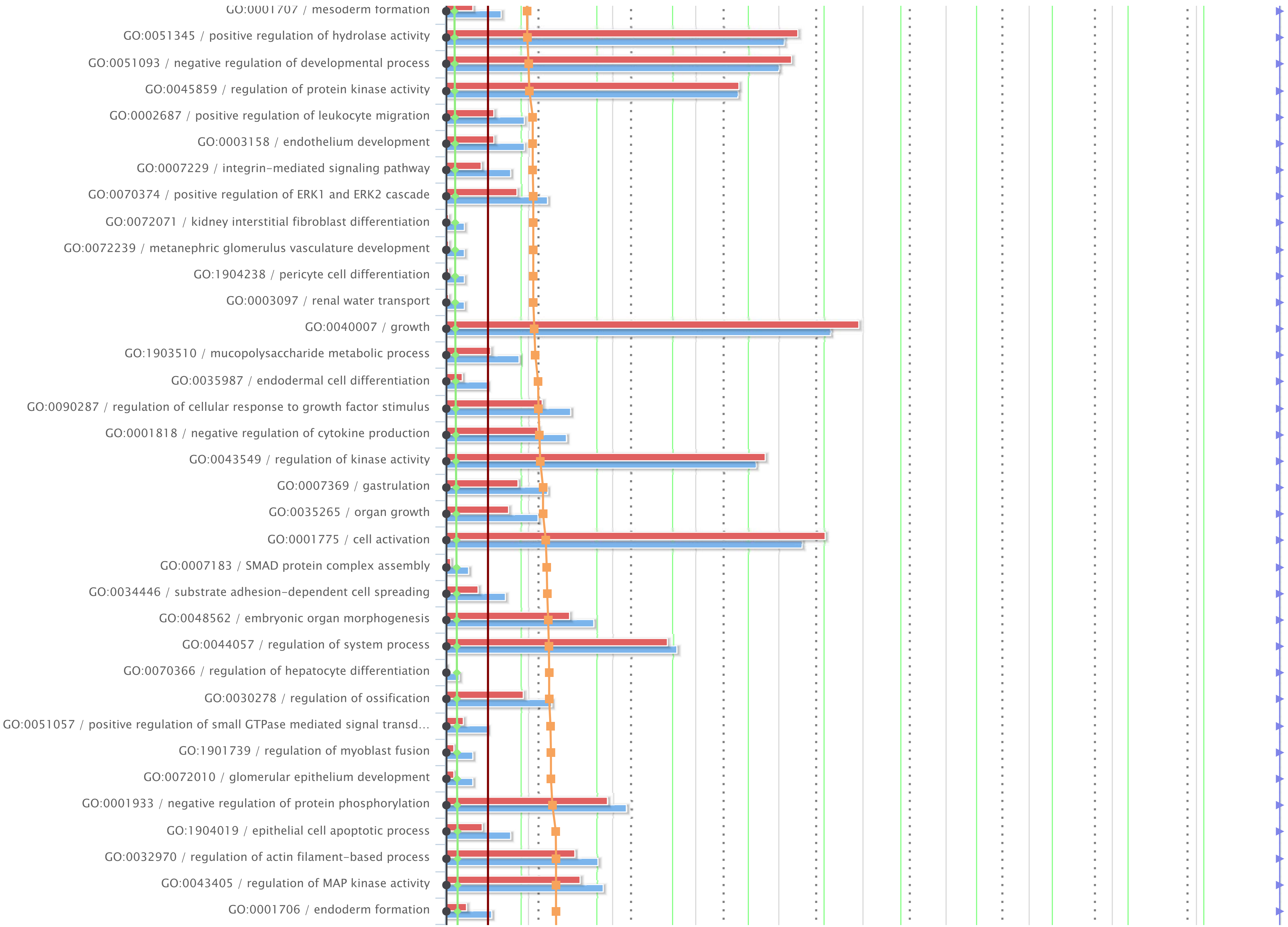
GO:0001707 / mesoderm formation
GO:0051345 / positive regulation of hydrolase activity
GO:0051093 / negative regulation of developmental process
GO:0045859 / regulation of protein kinase activity
GO:0002687 / positive regulation of leukocyte migration
GO:0003158 / endothelium development
GO:0007229 / integrin-mediated signaling pathway
GO:0070374 / positive regulation of ERK1 and ERK2 cascade
GO:0072071 / kidney interstitial fibroblast differentiation
GO:0072239 / metanephric glomerulus vasculature development
GO:1904238 / pericyte cell differentiation
GO:0003097 / renal water transport
GO:0040007 / growth
GO:1903510 / mucopolysaccharide metabolic process
GO:0035987 / endodermal cell differentiation
GO:0090287 / regulation of cellular response to growth factor stimulus
GO:0001818 / negative regulation of cytokine production
GO:0043549 / regulation of kinase activity
GO:0007369 / gastrulation
GO:0035265 / organ growth
GO:0001775 / cell activation
GO:0007183 / SMAD protein complex assembly
GO:0034446 / substrate adhesion-dependent cell spreading
GO:0048562 / embryonic organ morphogenesis
GO:0044057 / regulation of system process
GO:0070366 / regulation of hepatocyte differentiation
GO:0030278 / regulation of ossification
GO:0051057 / positive regulation of small GTPase mediated signal transd...
GO:1901739 / regulation of myoblast fusion
GO:0072010 / glomerular epithelium development
GO:0001933 / negative regulation of protein phosphorylation
GO:1904019 / epithelial cell apoptotic process
GO:0032970 / regulation of actin filament-based process
GO:0043405 / regulation of MAP kinase activity
GO:0001706 / endoderm formation

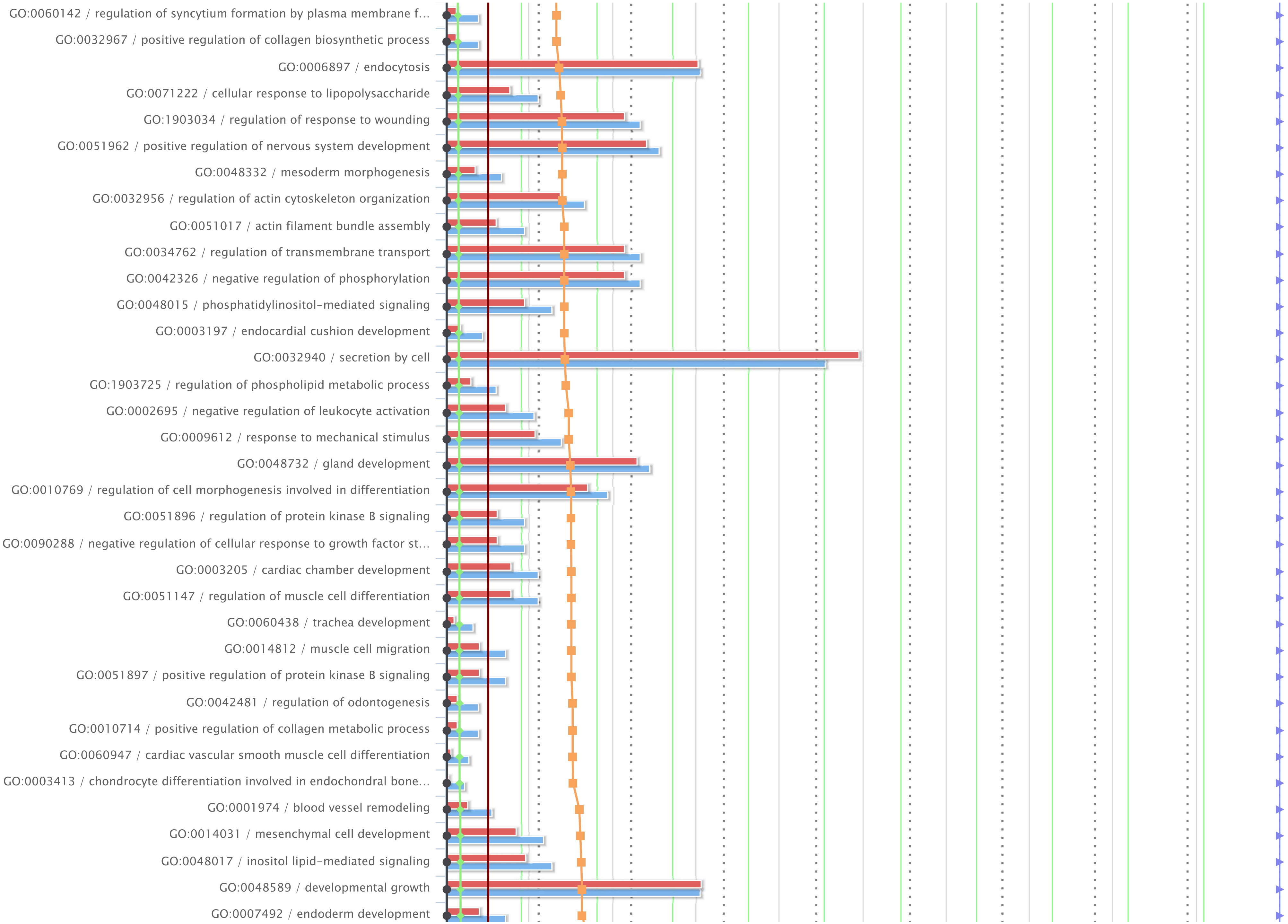
GO:0060142 / regulation of syncytium formation by plasma membrane f...
GO:0032967 / positive regulation of collagen biosynthetic process
GO:0006897 / endocytosis
GO:0071222 / cellular response to lipopolysaccharide
GO:1903034 / regulation of response to wounding
GO:0051962 / positive regulation of nervous system development
GO:0048332 / mesoderm morphogenesis
GO:0032956 / regulation of actin cytoskeleton organization
GO:0051017 / actin filament bundle assembly
GO:0034762 / regulation of transmembrane transport
GO:0042326 / negative regulation of phosphorylation
GO:0048015 / phosphatidylinositol−mediated signaling
GO:0003197 / endocardial cushion development
GO:0032940 / secretion by cell
GO:1903725 / regulation of phospholipid metabolic process
GO:0002695 / negative regulation of leukocyte activation
GO:0009612 / response to mechanical stimulus
GO:0048732 / gland development
GO:0010769 / regulation of cell morphogenesis involved in differentiation
GO:0051896 / regulation of protein kinase B signaling
GO:0090288 / negative regulation of cellular response to growth factor st...
GO:0003205 / cardiac chamber development
GO:0051147 / regulation of muscle cell differentiation
GO:0060438 / trachea development
GO:0014812 / muscle cell migration
GO:0051897 / positive regulation of protein kinase B signaling
GO:0042481 / regulation of odontogenesis
GO:0010714 / positive regulation of collagen metabolic process
GO:0060947 / cardiac vascular smooth muscle cell differentiation
GO:0003413 / chondrocyte differentiation involved in endochondral bone...
GO:0001974 / blood vessel remodeling
GO:0014031 / mesenchymal cell development
GO:0048017 / inositol lipid−mediated signaling
GO:0048589 / developmental growth
GO:0007492 / endoderm development

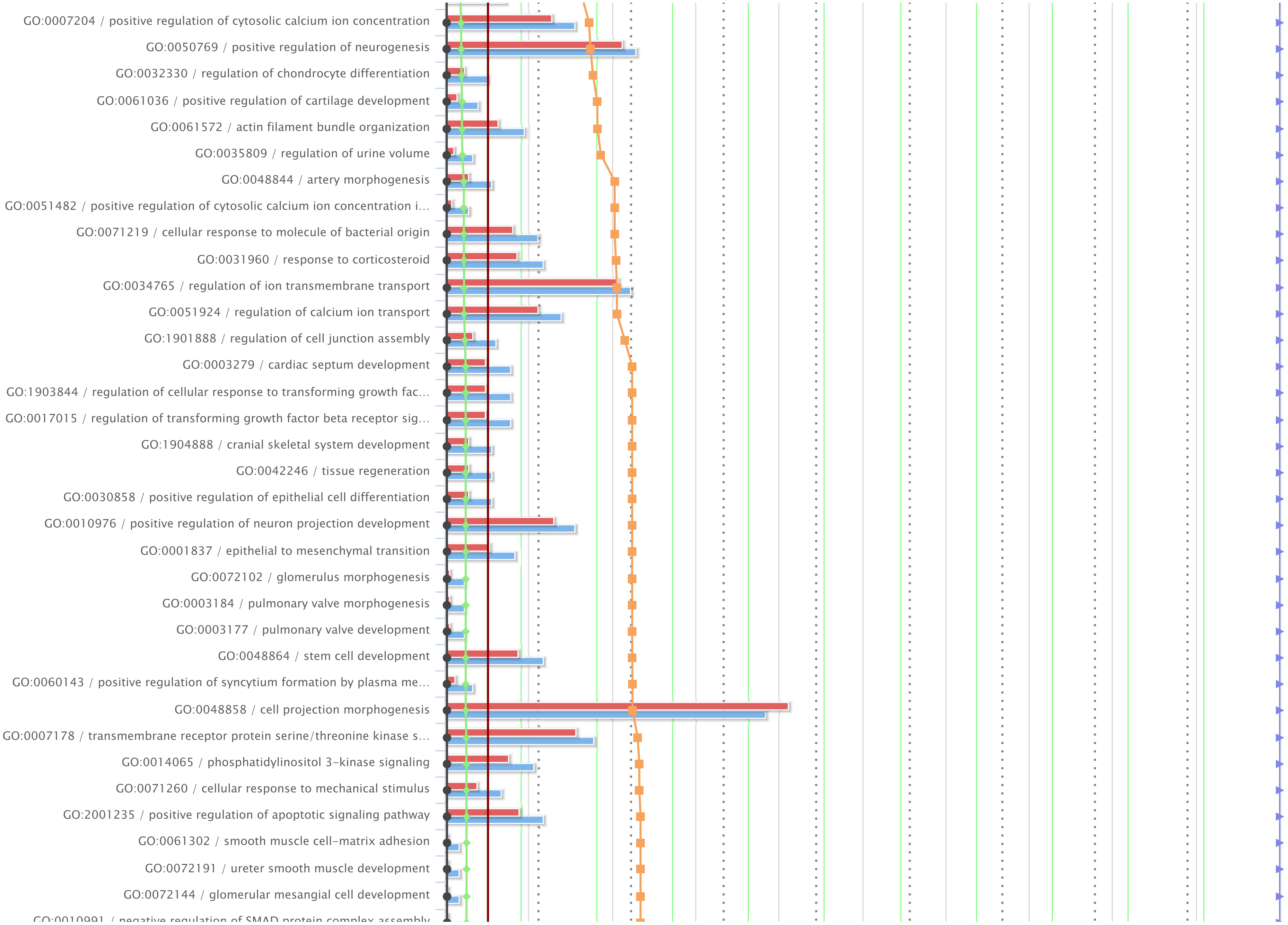
GO:0007204 / positive regulation of cytosolic calcium ion concentration
GO:0050769 / positive regulation of neurogenesis
GO:0032330 / regulation of chondrocyte differentiation
GO:0061036 / positive regulation of cartilage development
GO:0061572 / actin filament bundle organization
GO:0035809 / regulation of urine volume
GO:0048844 / artery morphogenesis
GO:0051482 / positive regulation of cytosolic calcium ion concentration i...
GO:0071219 / cellular response to molecule of bacterial origin
GO:0031960 / response to corticosteroid
GO:0034765 / regulation of ion transmembrane transport
GO:0051924 / regulation of calcium ion transport
GO:1901888 / regulation of cell junction assembly
GO:0003279 / cardiac septum development
GO:1903844 / regulation of cellular response to transforming growth fac...
GO:0017015 / regulation of transforming growth factor beta receptor sig...
GO:1904888 / cranial skeletal system development
GO:0042246 / tissue regeneration
GO:0030858 / positive regulation of epithelial cell differentiation
GO:0010976 / positive regulation of neuron projection development
GO:0001837 / epithelial to mesenchymal transition
GO:0072102 / glomerulus morphogenesis
GO:0003184 / pulmonary valve morphogenesis
GO:0003177 / pulmonary valve development
GO:0048864 / stem cell development
GO:0060143 / positive regulation of syncytium formation by plasma me...
GO:0048858 / cell projection morphogenesis
GO:0007178 / transmembrane receptor protein serine/threonine kinase s...
GO:0014065 / phosphatidylinositol 3-kinase signaling
GO:0071260 / cellular response to mechanical stimulus
GO:2001235 / positive regulation of apoptotic signaling pathway
GO:0061302 / smooth muscle cell-matrix adhesion
GO:0072191 / ureter smooth muscle development
GO:0072144 / glomerular mesangial cell development
GO:0010991 / negative regulation of SMAD protein complex assembly

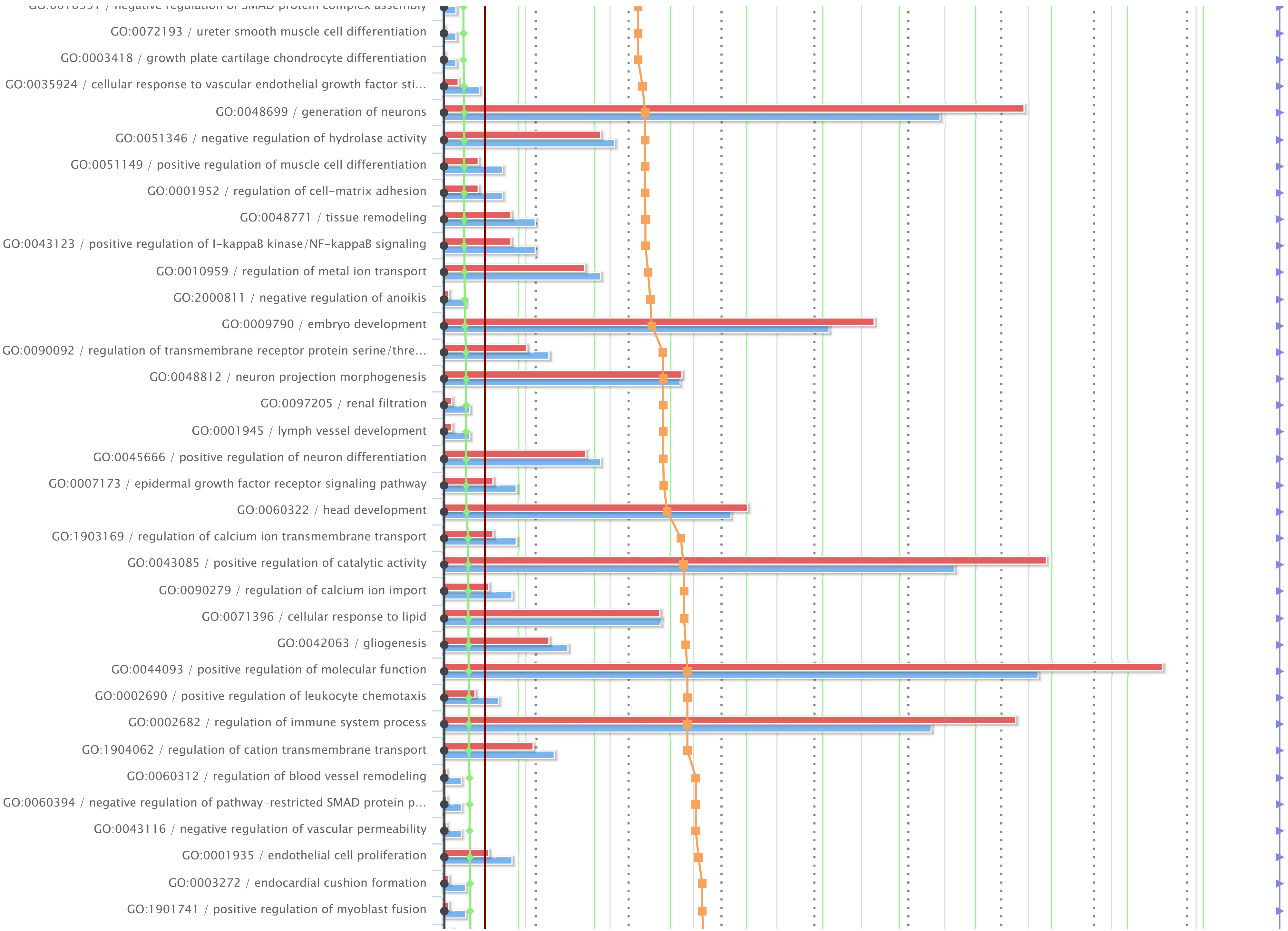
GO:0072193 / ureter smooth muscle cell differentiation
GO:0003418 / growth plate cartilage chondrocyte differentiation
GO:0035924 / cellular response to vascular endothelial growth factor sti...
GO:0048699 / generation of neurons
GO:0051346 / negative regulation of hydrolase activity
GO:0051149 / positive regulation of muscle cell differentiation
GO:0001952 / regulation of cell−matrix adhesion
GO:0048771 / tissue remodeling
GO:0043123 / positive regulation of I−kappaB kinase/NF−kappaB signaling
GO:0010959 / regulation of metal ion transport
GO:2000811 / negative regulation of anoikis
GO:0009790 / embryo development
GO:0090092 / regulation of transmembrane receptor protein serine/thre...
GO:0048812 / neuron projection morphogenesis
GO:0097205 / renal filtration
GO:0001945 / lymph vessel development
GO:0045666 / positive regulation of neuron differentiation
GO:0007173 / epidermal growth factor receptor signaling pathway
GO:0060322 / head development
GO:1903169 / regulation of calcium ion transmembrane transport
GO:0043085 / positive regulation of catalytic activity
GO:0090279 / regulation of calcium ion import
GO:0071396 / cellular response to lipid
GO:0042063 / gliogenesis
GO:0044093 / positive regulation of molecular function
GO:0002690 / positive regulation of leukocyte chemotaxis
GO:0002682 / regulation of immune system process
GO:1904062 / regulation of cation transmembrane transport
GO:0060312 / regulation of blood vessel remodeling
GO:0060394 / negative regulation of pathway−restricted SMAD protein p...
GO:0043116 / negative regulation of vascular permeability
GO:0001935 / endothelial cell proliferation
GO:0003272 / endocardial cushion formation
GO:1901741 / positive regulation of myoblast fusion

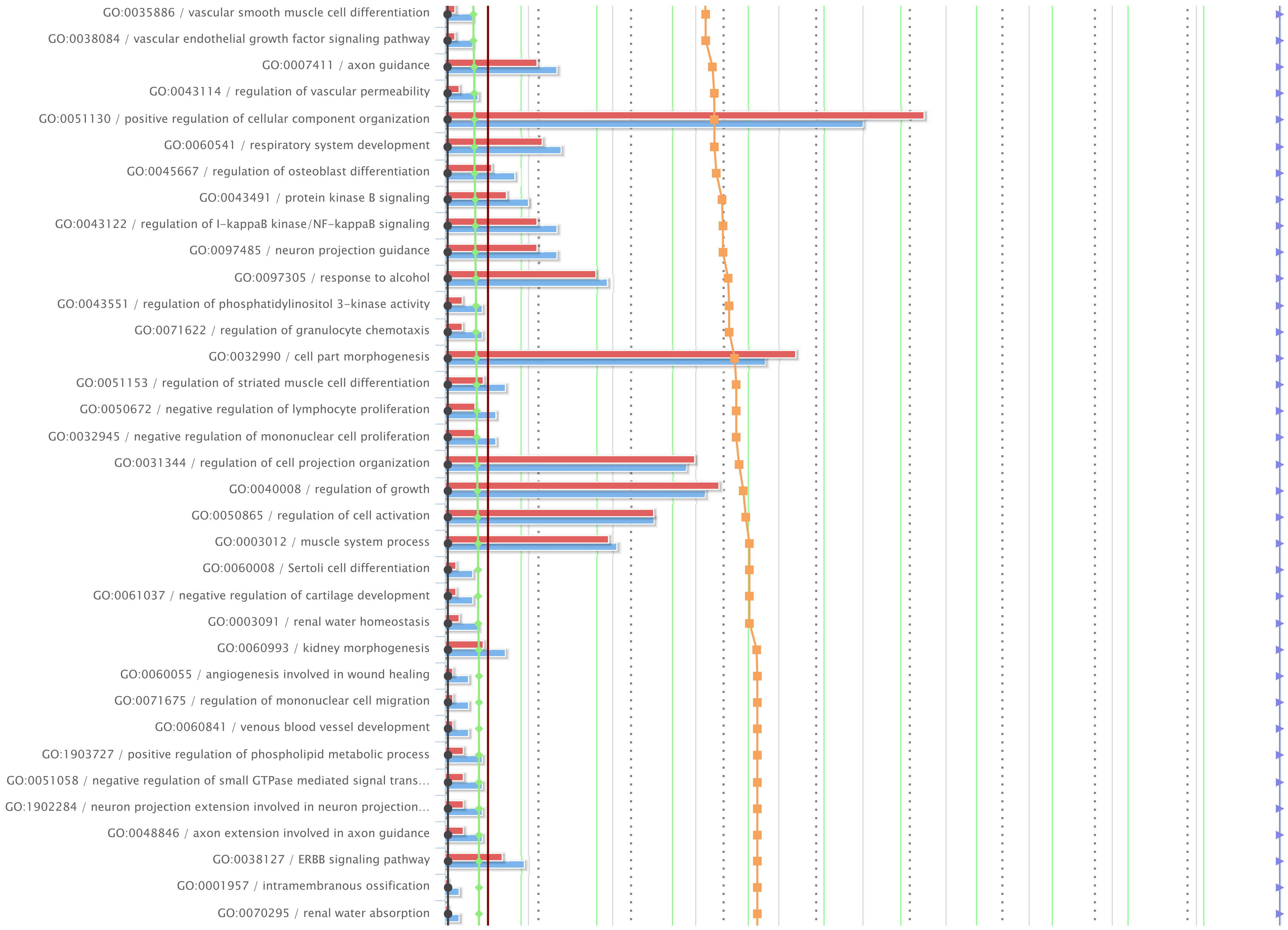
GO:0035886 / vascular smooth muscle cell differentiation
GO:0038084 / vascular endothelial growth factor signaling pathway
GO:0007411 / axon guidance
GO:0043114 / regulation of vascular permeability
GO:0051130 / positive regulation of cellular component organization
GO:0060541 / respiratory system development
GO:0045667 / regulation of osteoblast differentiation
GO:0043491 / protein kinase B signaling
GO:0043122 / regulation of I-kappaB kinase/NF-kappaB signaling
GO:0097485 / neuron projection guidance
GO:0097305 / response to alcohol
GO:0043551 / regulation of phosphatidylinositol 3-kinase activity
GO:0071622 / regulation of granulocyte chemotaxis
GO:0032990 / cell part morphogenesis
GO:0051153 / regulation of striated muscle cell differentiation
GO:0050672 / negative regulation of lymphocyte proliferation
GO:0032945 / negative regulation of mononuclear cell proliferation
GO:0031344 / regulation of cell projection organization
GO:0040008 / regulation of growth
GO:0050865 / regulation of cell activation
GO:0003012 / muscle system process
GO:0060008 / Sertoli cell differentiation
GO:0061037 / negative regulation of cartilage development
GO:0003091 / renal water homeostasis
GO:0060993 / kidney morphogenesis
GO:0060055 / angiogenesis involved in wound healing
GO:0071675 / regulation of mononuclear cell migration
GO:0060841 / venous blood vessel development
GO:1903727 / positive regulation of phospholipid metabolic process
GO:0051058 / negative regulation of small GTPase mediated signal trans...
GO:1902284 / neuron projection extension involved in neuron projection...
GO:0048846 / axon extension involved in axon guidance
GO:0038127 / ERBB signaling pathway
GO:0001957 / intramembranous ossification
GO:0070295 / renal water absorption

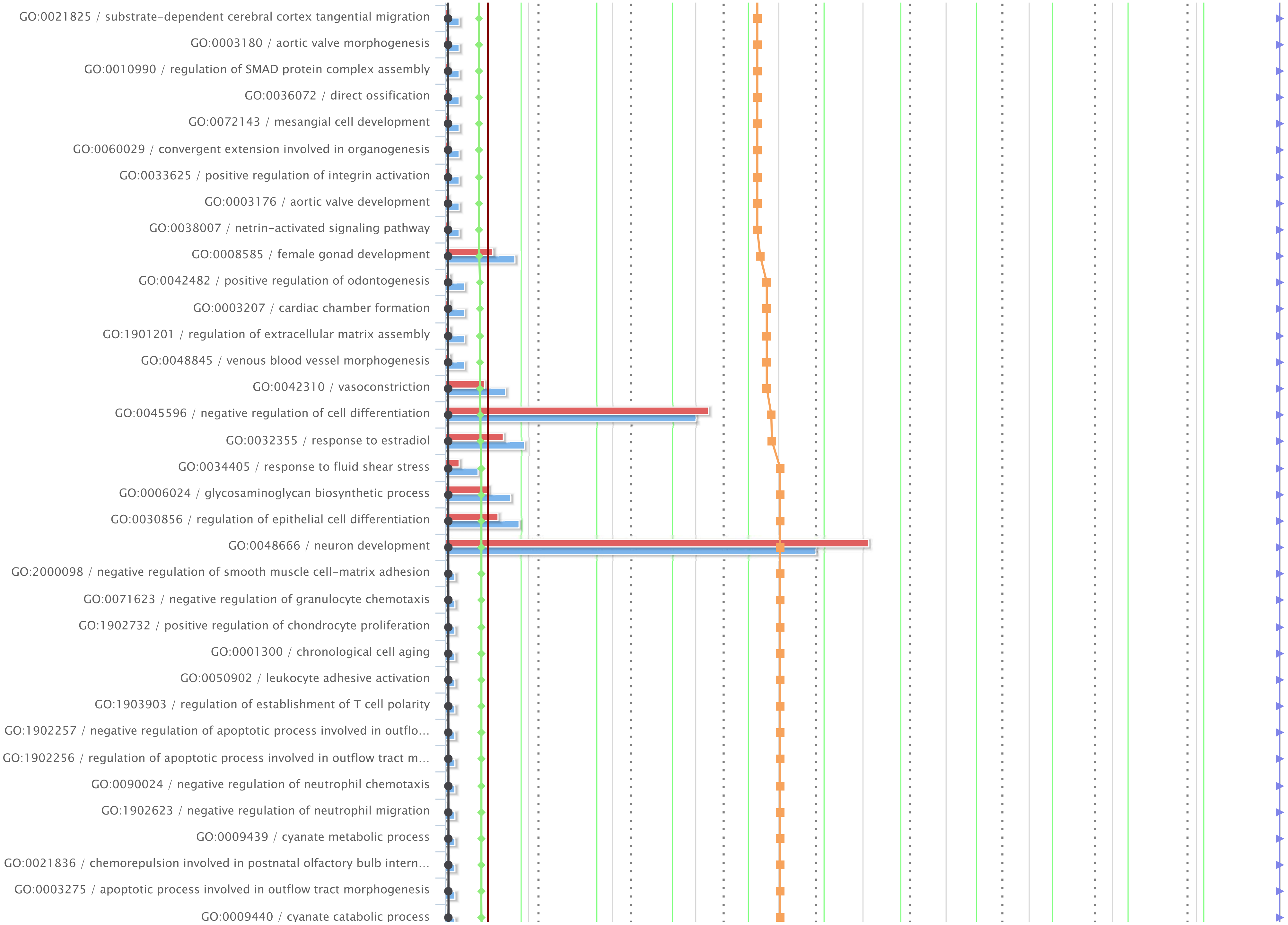
GO:0021825 / substrate-dependent cerebral cortex tangential migration
GO:0003180 / aortic valve morphogenesis
GO:0010990 / regulation of SMAD protein complex assembly
GO:0036072 / direct ossification
GO:0072143 / mesangial cell development
GO:0060029 / convergent extension involved in organogenesis
GO:0033625 / positive regulation of integrin activation
GO:0003176 / aortic valve development
GO:0038007 / netrin-activated signaling pathway
GO:0008585 / female gonad development
GO:0042482 / positive regulation of odontogenesis
GO:0003207 / cardiac chamber formation
GO:1901201 / regulation of extracellular matrix assembly
GO:0048845 / venous blood vessel morphogenesis
GO:0042310 / vasoconstriction
GO:0045596 / negative regulation of cell differentiation
GO:0032355 / response to estradiol
GO:0034405 / response to fluid shear stress
GO:0006024 / glycosaminoglycan biosynthetic process
GO:0030856 / regulation of epithelial cell differentiation
GO:0048666 / neuron development
GO:2000098 / negative regulation of smooth muscle cell-matrix adhesion
GO:0071623 / negative regulation of granulocyte chemotaxis
GO:1902732 / positive regulation of chondrocyte proliferation
GO:0001300 / chronological cell aging
GO:0050902 / leukocyte adhesive activation
GO:1903903 / regulation of establishment of T cell polarity
GO:1902257 / negative regulation of apoptotic process involved in outflo...
GO:1902256 / regulation of apoptotic process involved in outflow tract m...
GO:0090024 / negative regulation of neutrophil chemotaxis
GO:1902623 / negative regulation of neutrophil migration
GO:0009439 / cyanate metabolic process
GO:0021836 / chemorepulsion involved in postnatal olfactory bulb intern...
GO:0003275 / apoptotic process involved in outflow tract morphogenesis
GO:0009440 / cyanate catabolic process

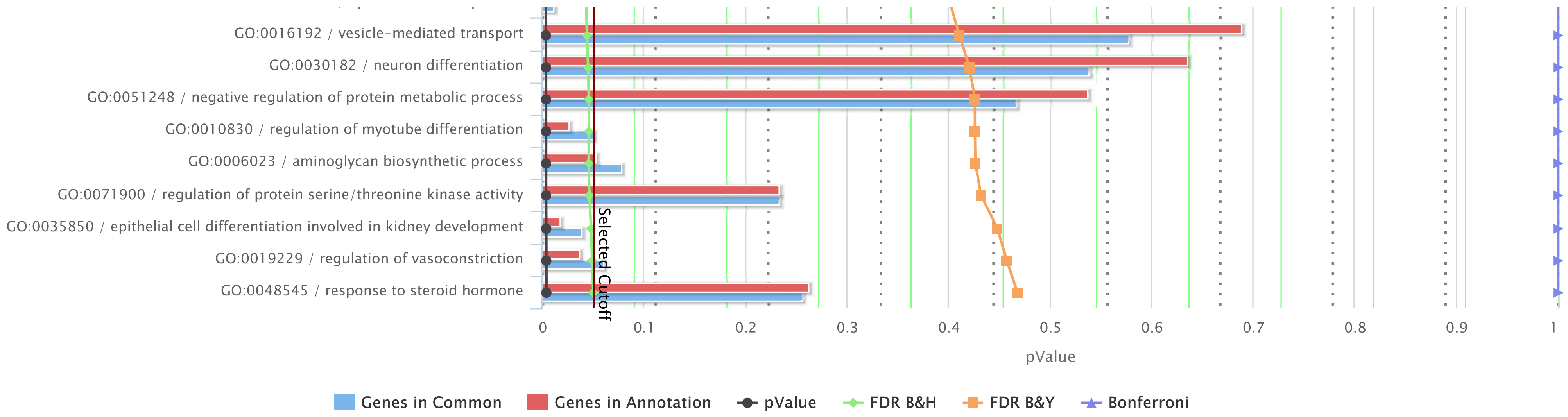
GO:0016192 / vesicle-mediated transport
GO:0030182 / neuron differentiation
GO:0051248 / negative regulation of protein metabolic process
GO:0010830 / regulation of myotube differentiation
GO:0006023 / aminoglycan biosynthetic process
GO:0071900 / regulation of protein serine/threonine kinase activity
GO:0035850 / epithelial cell differentiation involved in kidney development
GO:0019229 / regulation of vasoconstriction
GO:0048545 / response to steroid hormone
Selected Cutoff
0
0.1
0.2
0.3
0.4
0.5
0.6
0.7
0.8
0.9
1
pValue
Genes in Common
Genes in Annotation
pValue
FDR B&H
FDR B&Y
Bonferroni

Supplementary Figure 5C
Hyperacetylated genes

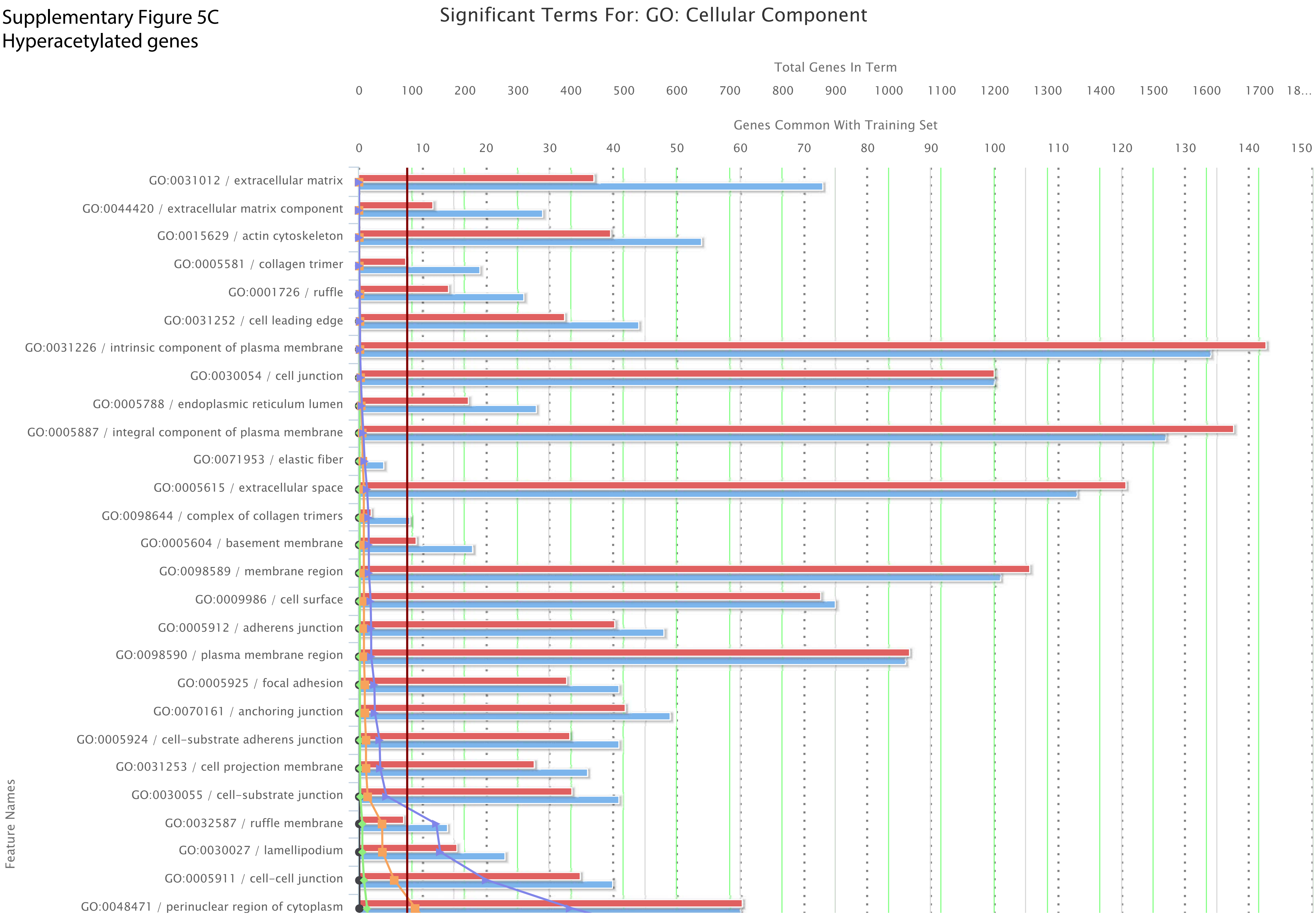

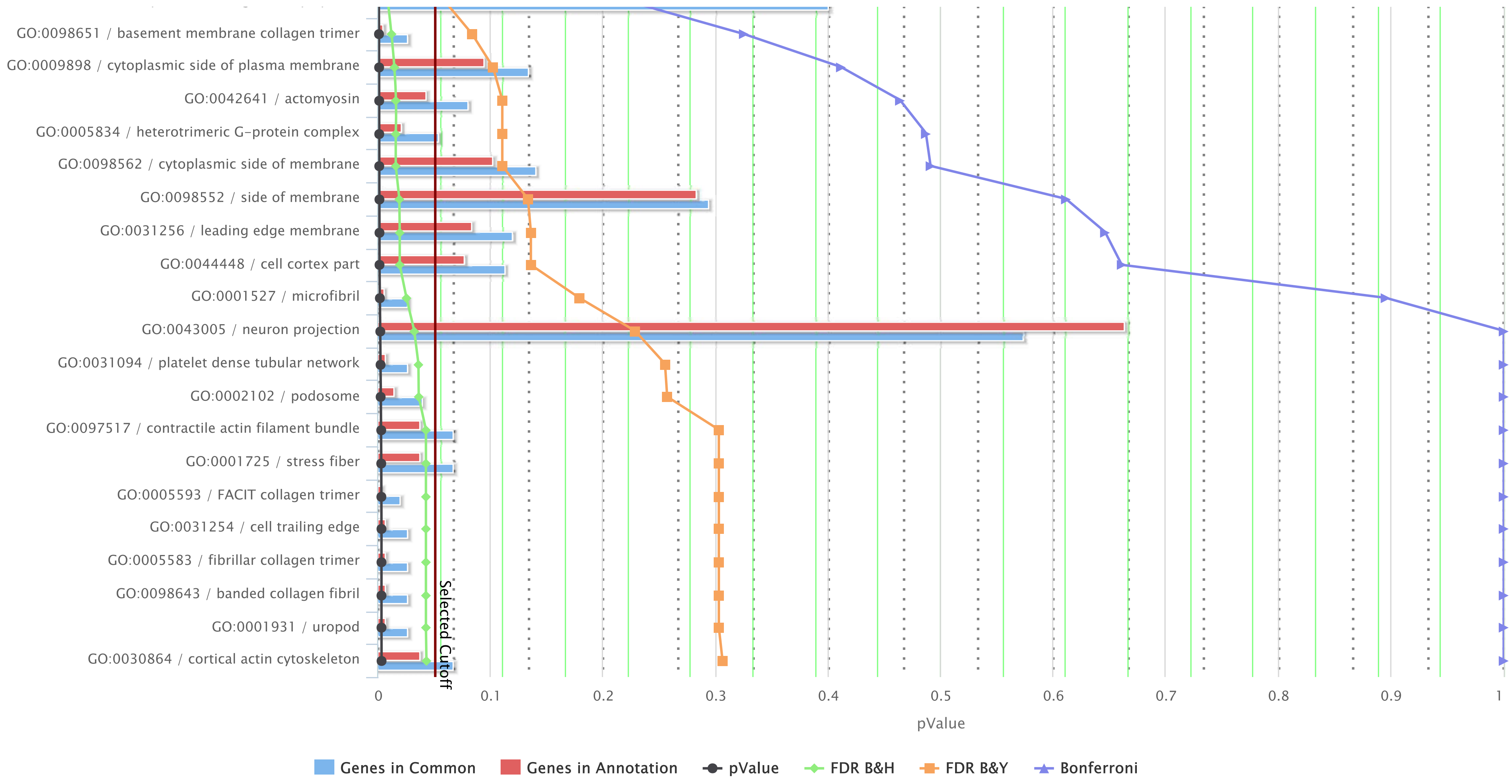
GO:0098651 / basement membrane collagen trimer
GO:0009898 / cytoplasmic side of plasma membrane
GO:0042641 / actomyosin
GO:0005834 / heterotrimeric G-protein complex
GO:0098562 / cytoplasmic side of membrane
GO:0098552 / side of membrane
GO:0031256 / leading edge membrane
GO:0044448 / cell cortex part
GO:0001527 / microfibril
GO:0043005 / neuron projection
GO:0031094 / platelet dense tubular network
GO:0002102 / podosome
GO:0097517 / contractile actin filament bundle
GO:0001725 / stress fiber
GO:0005593 / FACIT collagen trimer
GO:0031254 / cell trailing edge
GO:0005583 / fibrillar collagen trimer
GO:0098643 / banded collagen fibril
GO:0001931 / uropod
GO:0030864 / cortical actin cytoskeleton
Selected Cutoff
0
0.1
0.2
0.3
0.4
0.5
0.6
0.7
0.8
0.9
1
pValue
Genes in Common
Genes in Annotation
pValue
FDR B&H
FDR B&Y
Bonferroni

Supplementary Figure 5D
Hypoacetylated genes

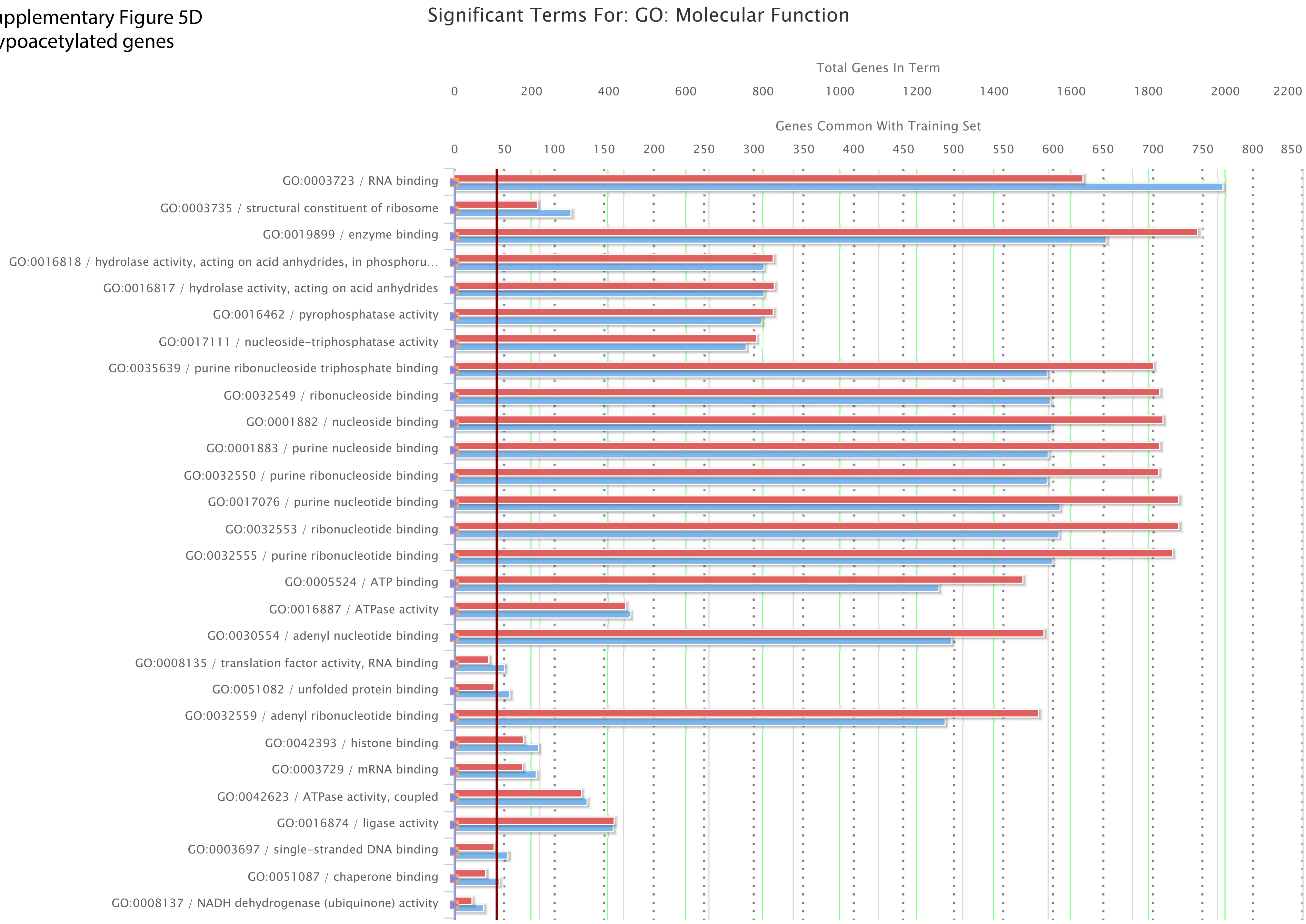

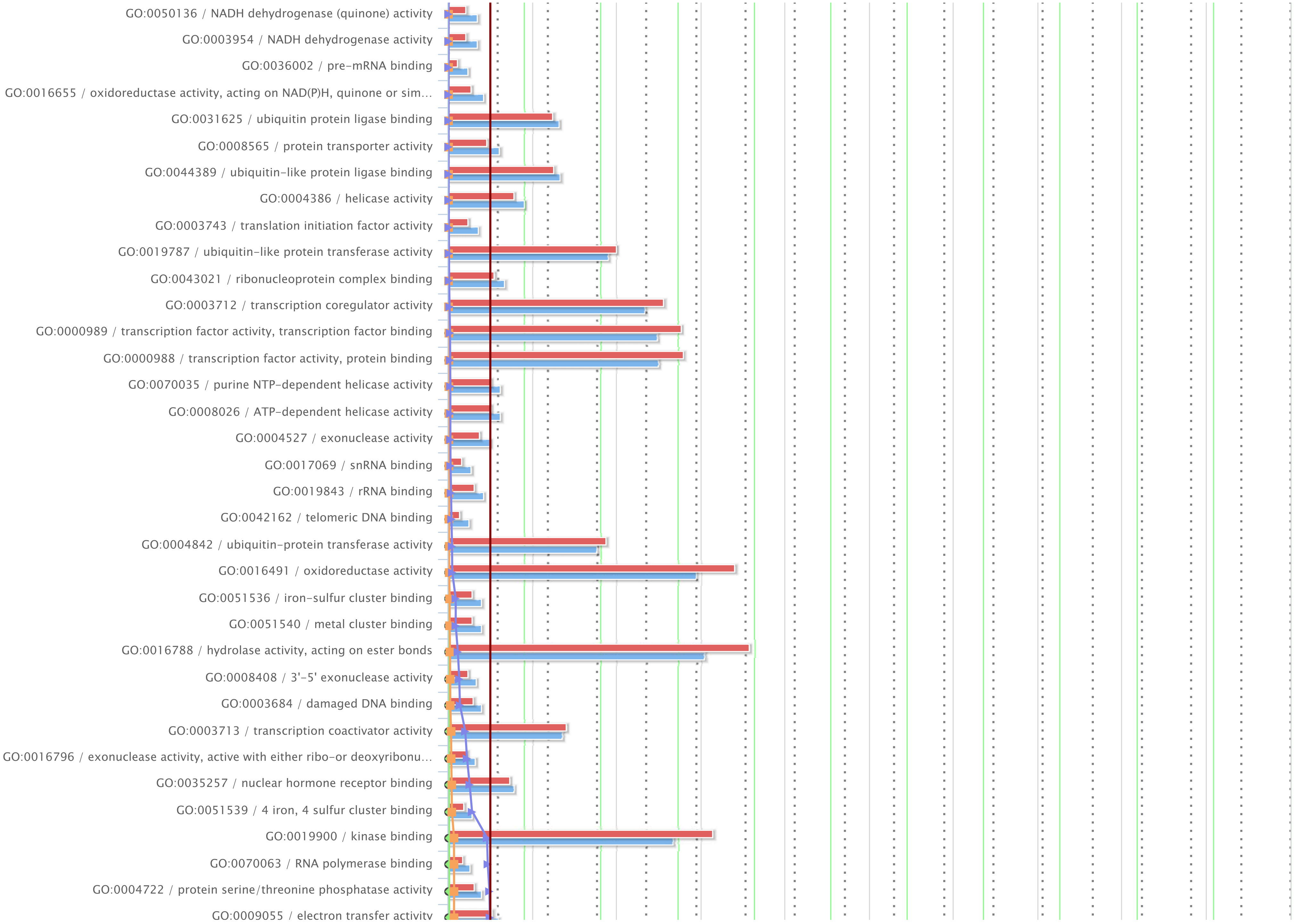

GO:0050136 / NADH dehydrogenase (quinone) activity
GO:0003954 / NADH dehydrogenase activity
GO:0036002 / pre−mRNA binding
GO:0016655 / oxidoreductase activity, acting on NAD(P)H, quinone or sim…
GO:0031625 / ubiquitin protein ligase binding
GO:0008565 / protein transporter activity
GO:0044389 / ubiquitin−like protein ligase binding
GO:0004386 / helicase activity
GO:0003743 / translation initiation factor activity
GO:0019787 / ubiquitin−like protein transferase activity
GO:0043021 / ribonucleoprotein complex binding
GO:0003712 / transcription coregulator activity
GO:0000989 / transcription factor activity, transcription factor binding
GO:0000988 / transcription factor activity, protein binding
GO:0070035 / purine NTP−dependent helicase activity
GO:0008026 / ATP−dependent helicase activity
GO:0004527 / exonuclease activity
GO:0017069 / snRNA binding
GO:0019843 / rRNA binding
GO:0042162 / telomeric DNA binding
GO:0004842 / ubiquitin−protein transferase activity
GO:0016491 / oxidoreductase activity
GO:0051536 / iron−sulfur cluster binding
GO:0051540 / metal cluster binding
GO:0016788 / hydrolase activity, acting on ester bonds
GO:0008408 / 3'−5' exonuclease activity
GO:0003684 / damaged DNA binding
GO:0003713 / transcription coactivator activity
GO:0016796 / exonuclease activity, active with either ribo−or deoxyribonu…
GO:0035257 / nuclear hormone receptor binding
GO:0051539 / 4 iron, 4 sulfur cluster binding
GO:0019900 / kinase binding
GO:0070063 / RNA polymerase binding
GO:0004722 / protein serine/threonine phosphatase activity
GO:0009055 / electron transfer activity

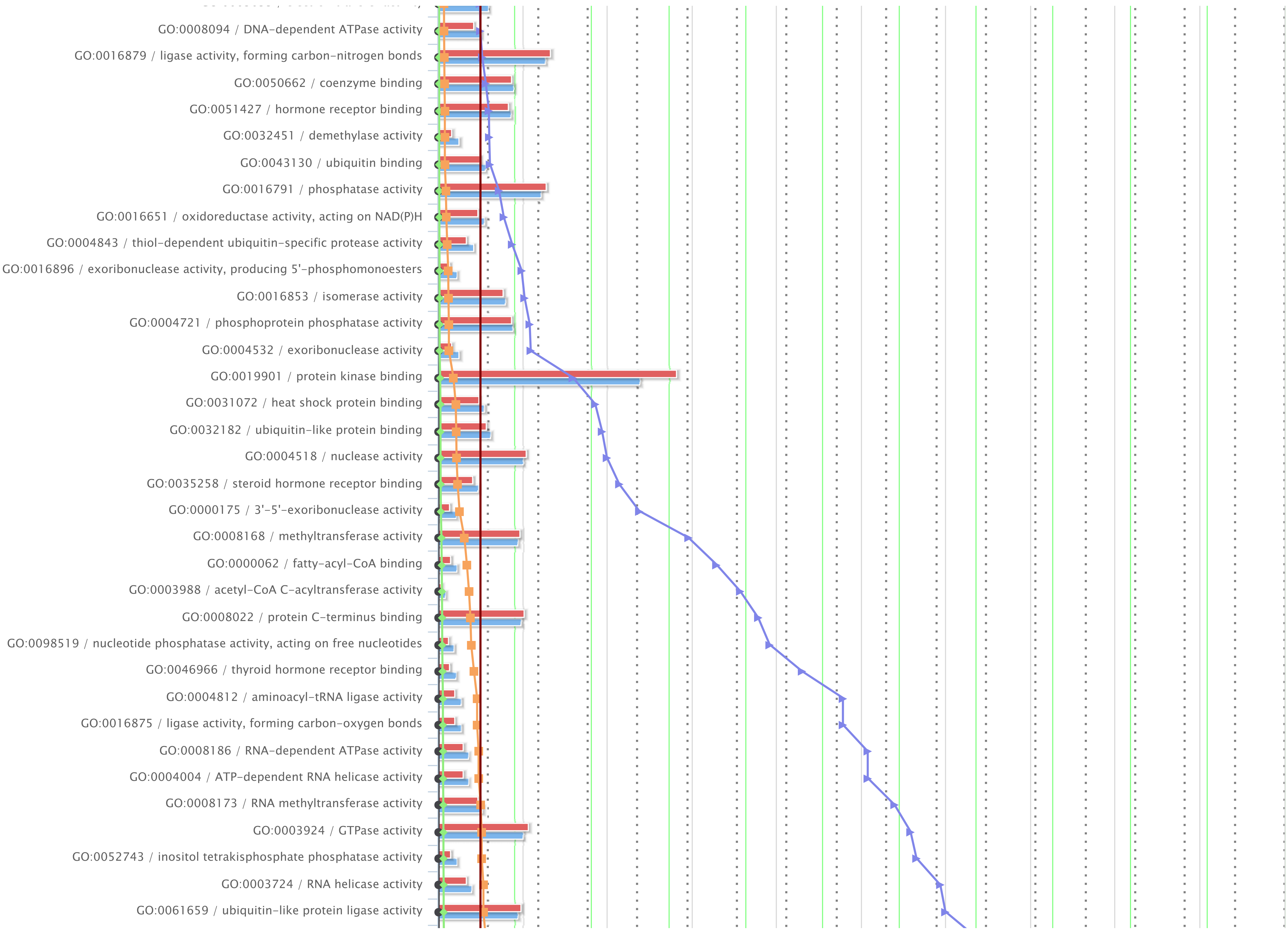
GO:0008094 / DNA-dependent ATPase activity
GO:0016879 / ligase activity, forming carbon-nitrogen bonds
GO:0050662 / coenzyme binding
GO:0051427 / hormone receptor binding
GO:0032451 / demethylase activity
GO:0043130 / ubiquitin binding
GO:0016791 / phosphatase activity
GO:0016651 / oxidoreductase activity, acting on NAD(P)H
GO:0004843 / thiol-dependent ubiquitin-specific protease activity
GO:0016896 / exoribonuclease activity, producing 5'-phosphomonoesters
GO:0016853 / isomerase activity
GO:0004721 / phosphoprotein phosphatase activity
GO:0004532 / exoribonuclease activity
GO:0019901 / protein kinase binding
GO:0031072 / heat shock protein binding
GO:0032182 / ubiquitin-like protein binding
GO:0004518 / nuclease activity
GO:0035258 / steroid hormone receptor binding
GO:0000175 / 3'-5'-exoribonuclease activity
GO:0008168 / methyltransferase activity
GO:0000062 / fatty-acyl-CoA binding
GO:0003988 / acetyl-CoA C-acyltransferase activity
GO:0008022 / protein C-terminus binding
GO:0098519 / nucleotide phosphatase activity, acting on free nucleotides
GO:0046966 / thyroid hormone receptor binding
GO:0004812 / aminoacyl-tRNA ligase activity
GO:0016875 / ligase activity, forming carbon-oxygen bonds
GO:0008186 / RNA-dependent ATPase activity
GO:0004004 / ATP-dependent RNA helicase activity
GO:0008173 / RNA methyltransferase activity
GO:0003924 / GTPase activity
GO:0052743 / inositol tetrakisphosphate phosphatase activity
GO:0003724 / RNA helicase activity
GO:0061659 / ubiquitin-like protein ligase activity

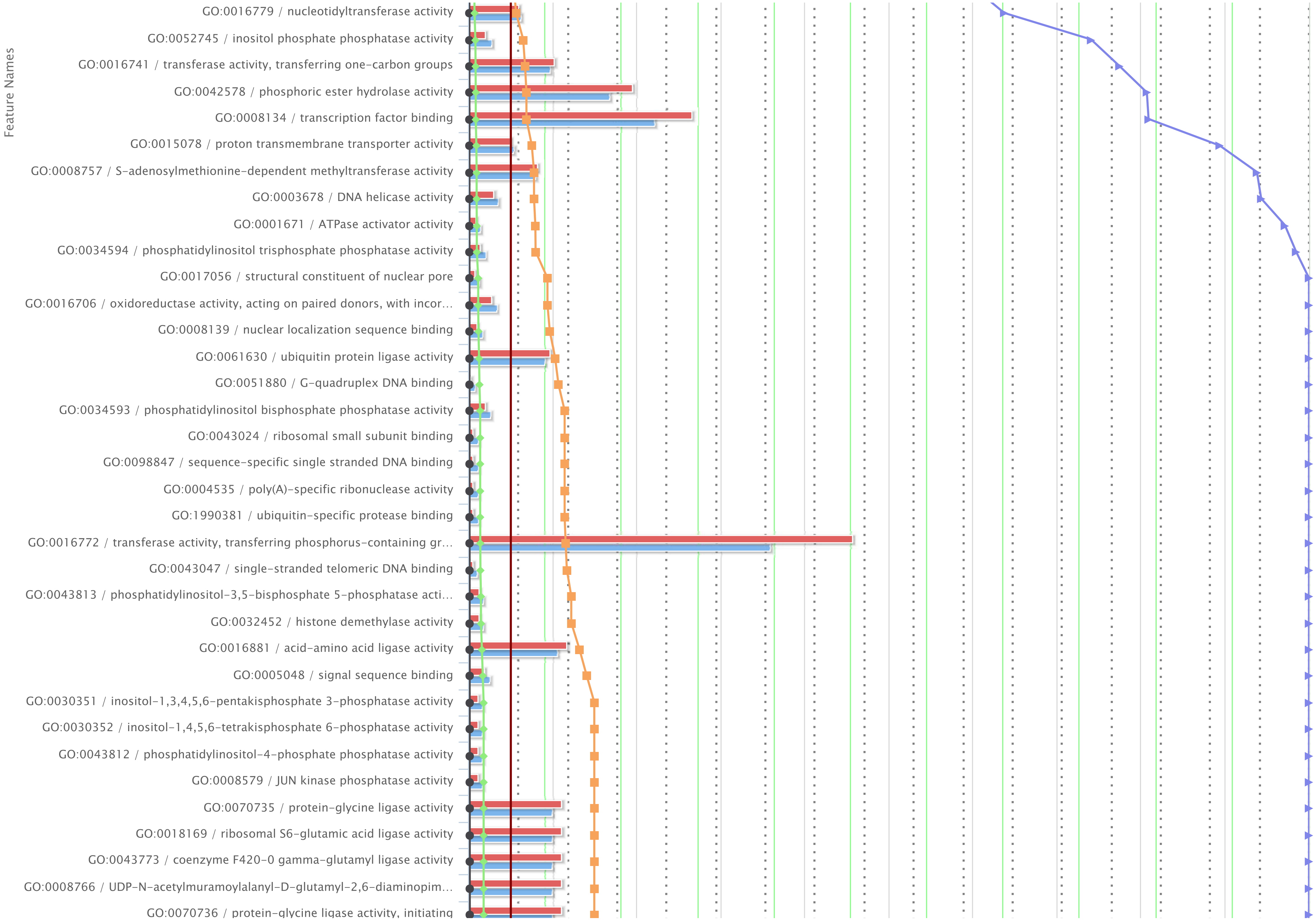

Feature Names
GO:0016779 / nucleotidyltransferase activity
GO:0052745 / inositol phosphate phosphatase activity
GO:0016741 / transferase activity, transferring one-carbon groups
GO:0042578 / phosphoric ester hydrolase activity
GO:0008134 / transcription factor binding
GO:0015078 / proton transmembrane transporter activity
GO:0008757 / S-adenosylmethionine-dependent methyltransferase activity
GO:0003678 / DNA helicase activity
GO:0001671 / ATPase activator activity
GO:0034594 / phosphatidylinositol trisphosphate phosphatase activity
GO:0017056 / structural constituent of nuclear pore
GO:0016706 / oxidoreductase activity, acting on paired donors, with incor...
GO:0008139 / nuclear localization sequence binding
GO:0061630 / ubiquitin protein ligase activity
GO:0051880 / G-quadruplex DNA binding
GO:0034593 / phosphatidylinositol bisphosphate phosphatase activity
GO:0043024 / ribosomal small subunit binding
GO:0098847 / sequence-specific single stranded DNA binding
GO:0004535 / poly(A)-specific ribonuclease activity
GO:1990381 / ubiquitin-specific protease binding
GO:0016772 / transferase activity, transferring phosphorus-containing gr...
GO:0043047 / single-stranded telomeric DNA binding
GO:0043813 / phosphatidylinositol-3,5-bisphosphate 5-phosphatase acti...
GO:0032452 / histone demethylase activity
GO:0016881 / acid-amino acid ligase activity
GO:0005048 / signal sequence binding
GO:0030351 / inositol-1,3,4,5,6-pentakisphosphate 3-phosphatase activity
GO:0030352 / inositol-1,4,5,6-tetrakisphosphate 6-phosphatase activity
GO:0043812 / phosphatidylinositol-4-phosphate phosphatase activity
GO:0008579 / JUN kinase phosphatase activity
GO:0070735 / protein-glycine ligase activity
GO:0018169 / ribosomal S6-glutamic acid ligase activity
GO:0043773 / coenzyme F420-0 gamma-glutamyl ligase activity
GO:0008766 / UDP-N-acetylmuramoylalanyl-D-glutamyl-2,6-diaminopim...
GO:0070736 / protein-glycine ligase activity, initiating

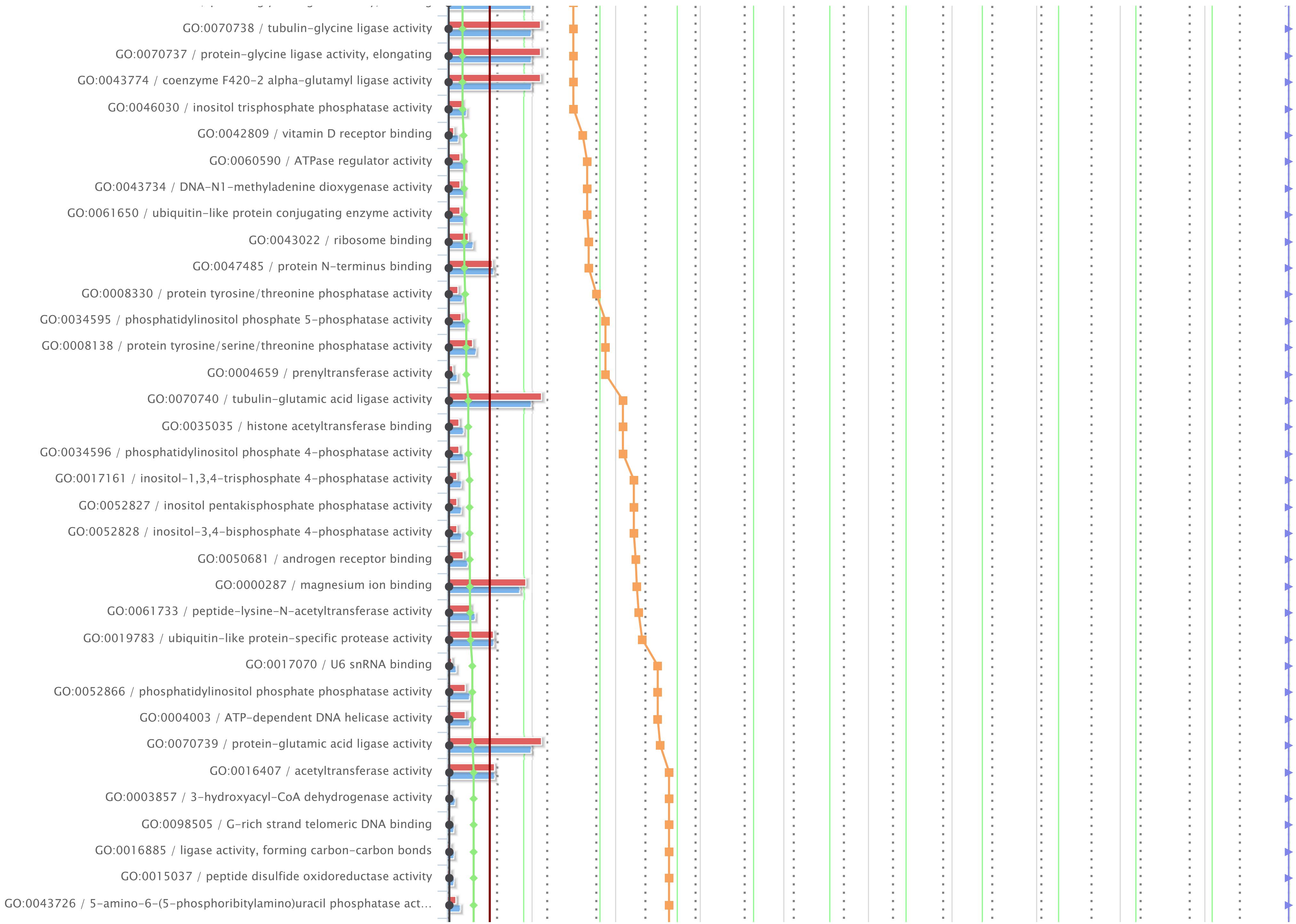
GO:0070738 / tubulin–glycine ligase activity
GO:0070737 / protein–glycine ligase activity, elongating
GO:0043774 / coenzyme F420–2 alpha–glutamyl ligase activity
GO:0046030 / inositol trisphosphate phosphatase activity
GO:0042809 / vitamin D receptor binding
GO:0060590 / ATPase regulator activity
GO:0043734 / DNA–N1–methyladenine dioxygenase activity
GO:0061650 / ubiquitin–like protein conjugating enzyme activity
GO:0043022 / ribosome binding
GO:0047485 / protein N–terminus binding
GO:0008330 / protein tyrosine/threonine phosphatase activity
GO:0034595 / phosphatidylinositol phosphate 5–phosphatase activity
GO:0008138 / protein tyrosine/serine/threonine phosphatase activity
GO:0004659 / prenyltransferase activity
GO:0070740 / tubulin–glutamic acid ligase activity
GO:0035035 / histone acetyltransferase binding
GO:0034596 / phosphatidylinositol phosphate 4–phosphatase activity
GO:0017161 / inositol–1,3,4–trisphosphate 4–phosphatase activity
GO:0052827 / inositol pentakisphosphate phosphatase activity
GO:0052828 / inositol–3,4–bisphosphate 4–phosphatase activity
GO:0050681 / androgen receptor binding
GO:0000287 / magnesium ion binding
GO:0061733 / peptide–lysine–N–acetyltransferase activity
GO:0019783 / ubiquitin–like protein–specific protease activity
GO:0017070 / U6 snRNA binding
GO:0052866 / phosphatidylinositol phosphate phosphatase activity
GO:0004003 / ATP–dependent DNA helicase activity
GO:0070739 / protein–glutamic acid ligase activity
GO:0016407 / acetyltransferase activity
GO:0003857 / 3–hydroxyacyl–CoA dehydrogenase activity
GO:0098505 / G–rich strand telomeric DNA binding
GO:0016885 / ligase activity, forming carbon–carbon bonds
GO:0015037 / peptide disulfide oxidoreductase activity
GO:0043726 / 5–amino–6–(5–phosphoribitylamino)uracil phosphatase act...

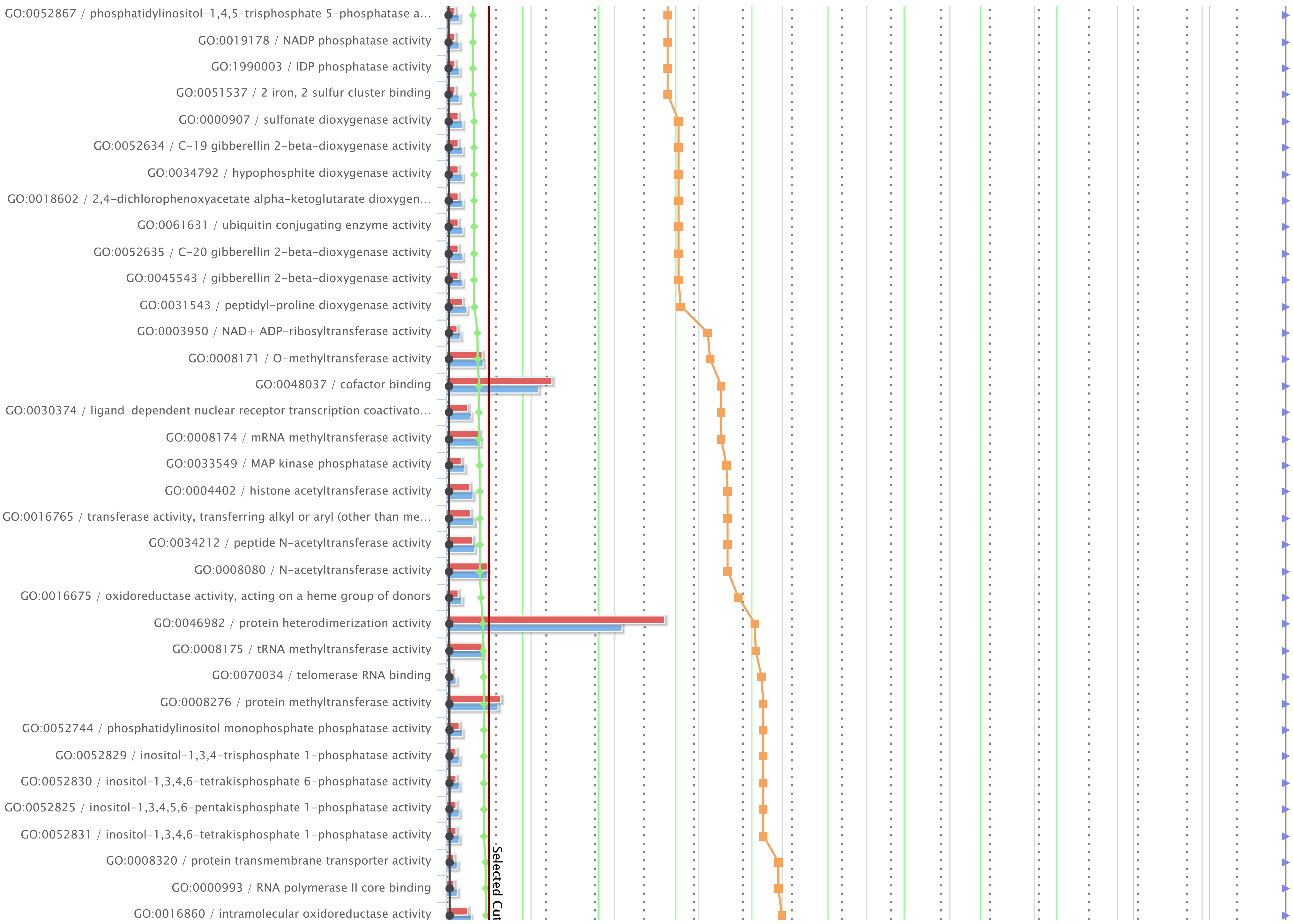
GO:0052867 / phosphatidylinositol-1,4,5-trisphosphate 5-phosphatase a...
GO:0019178 / NADP phosphatase activity
GO:1990003 / IDP phosphatase activity
GO:0051537 / 2 iron, 2 sulfur cluster binding
GO:0000907 / sulfonate dioxygenase activity
GO:0052634 / C-19 gibberellin 2-beta-dioxygenase activity
GO:0034792 / hypophosphite dioxygenase activity
GO:0018602 / 2,4-dichlorophenoxyacetate alpha-ketoglutarate dioxygen...
GO:0061631 / ubiquitin conjugating enzyme activity
GO:0052635 / C-20 gibberellin 2-beta-dioxygenase activity
GO:0045543 / gibberellin 2-beta-dioxygenase activity
GO:0031543 / peptidyl-proline dioxygenase activity
GO:0003950 / NAD+ ADP-ribosyltransferase activity
GO:0008171 / O-methyltransferase activity
GO:0048037 / cofactor binding
GO:0030374 / ligand-dependent nuclear receptor transcription coactivato...
GO:0008174 / mRNA methyltransferase activity
GO:0033549 / MAP kinase phosphatase activity
GO:0004402 / histone acetyltransferase activity
GO:0016765 / transferase activity, transferring alkyl or aryl (other than me...
GO:0034212 / peptide N-acetyltransferase activity
GO:0008080 / N-acetyltransferase activity
GO:0016675 / oxidoreductase activity, acting on a heme group of donors
GO:0046982 / protein heterodimerization activity
GO:0008175 / tRNA methyltransferase activity
GO:0070034 / telomerase RNA binding
GO:0008276 / protein methyltransferase activity
GO:0052744 / phosphatidylinositol monophosphate phosphatase activity
GO:0052829 / inositol-1,3,4-trisphosphate 1-phosphatase activity
GO:0052830 / inositol-1,3,4,6-tetrakisphosphate 6-phosphatase activity
GO:0052825 / inositol-1,3,4,5,6-pentakisphosphate 1-phosphatase activity
GO:0052831 / inositol-1,3,4,6-tetrakisphosphate 1-phosphatase activity
GO:0008320 / protein transmembrane transporter activity
GO:0000993 / RNA polymerase II core binding
GO:0016860 / intramolecular oxidoreductase activity
Selected Cut

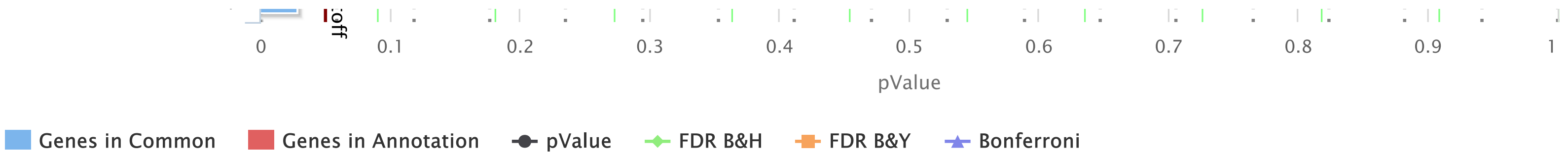
0
0.1
0.2
0.3
0.4
0.5
0.6
0.7
0.8
0.9
1
pValue
Genes in Common
Genes in Annotation
pValue
FDR B&H
FDR B&Y
Bonferroni

Supplementary Figure 5E
Hypoacetylated genes

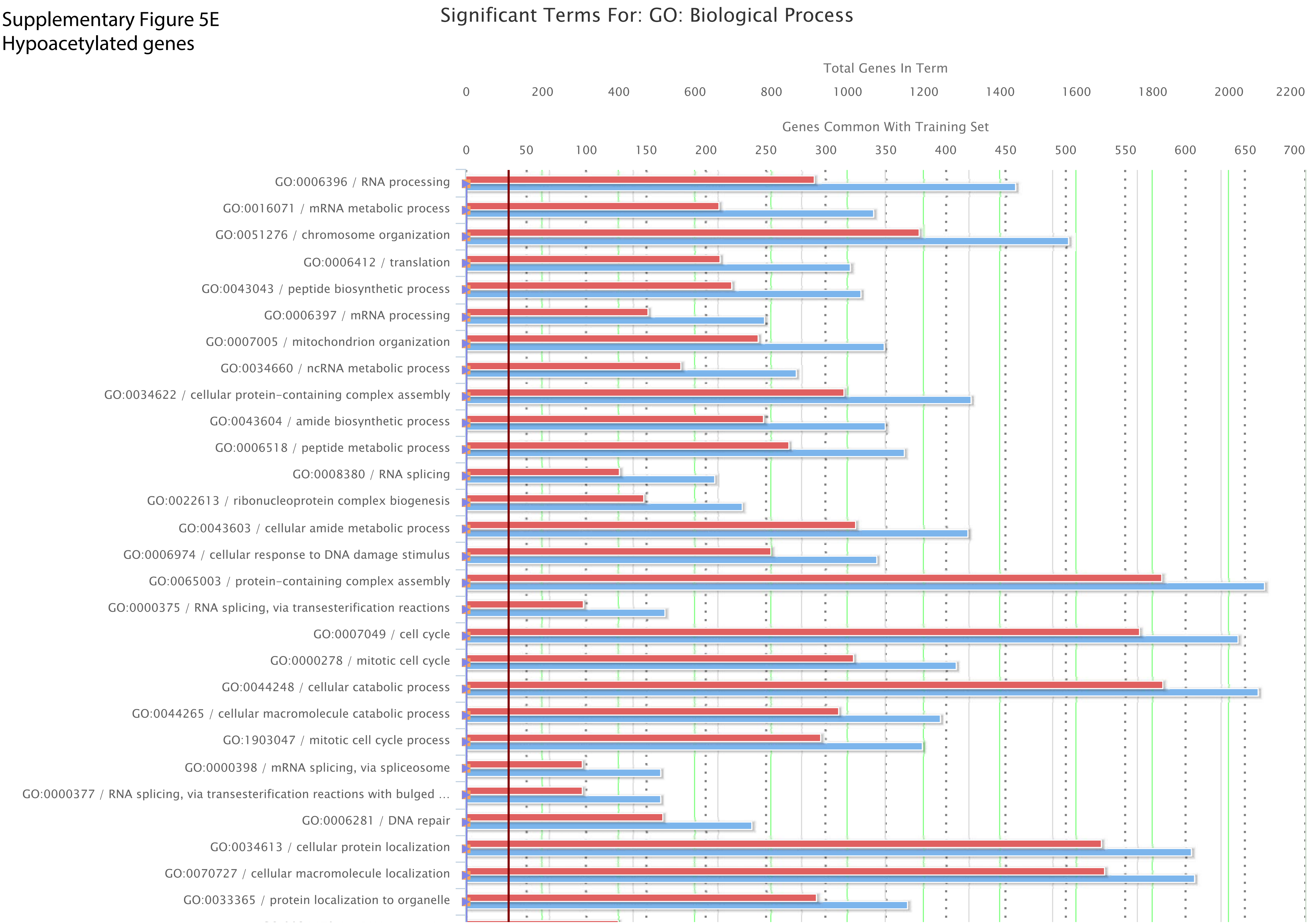

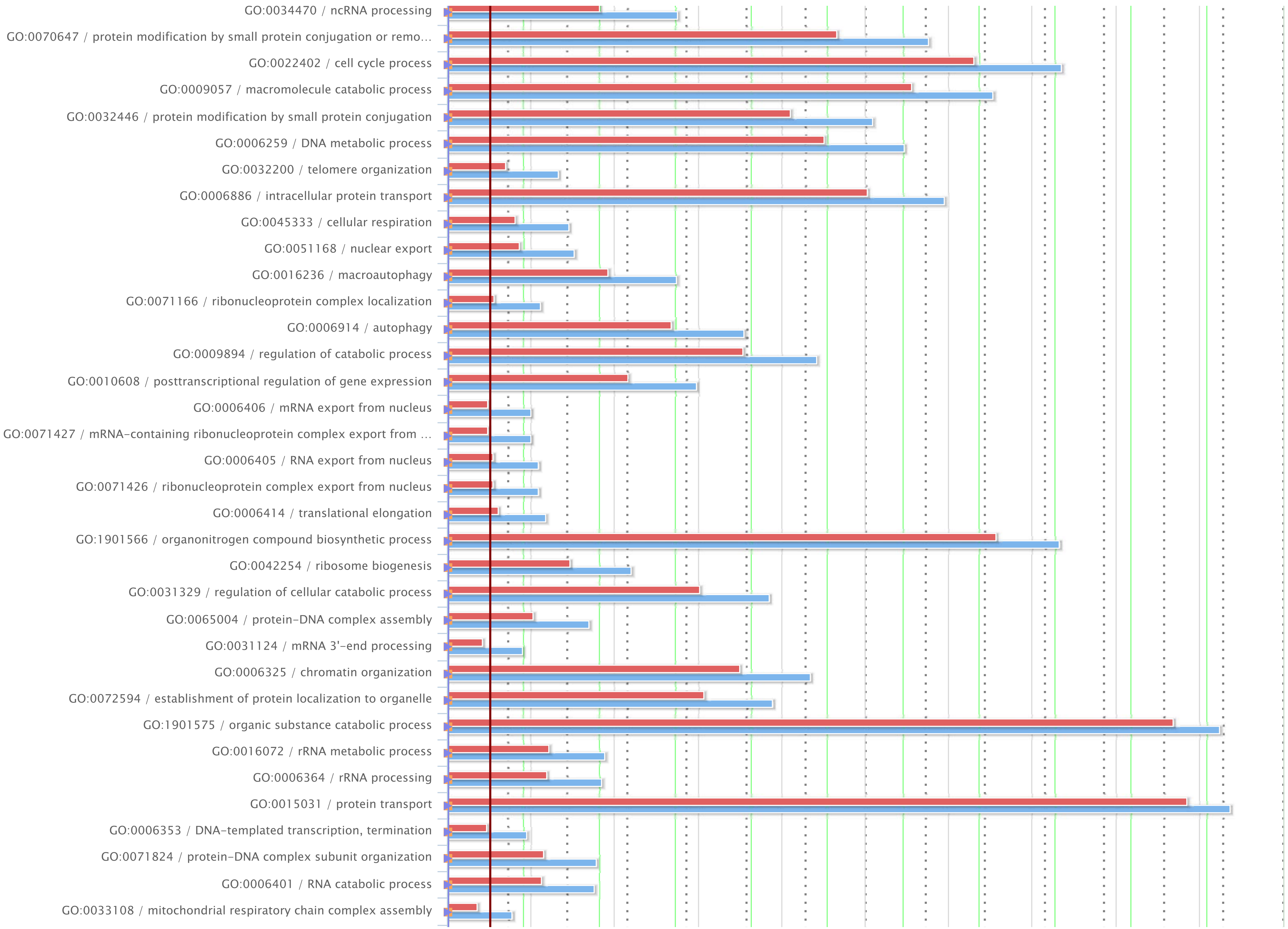
GO:0034470 / ncRNA processing
GO:0070647 / protein modification by small protein conjugation or remo...
GO:0022402 / cell cycle process
GO:0009057 / macromolecule catabolic process
GO:0032446 / protein modification by small protein conjugation
GO:0006259 / DNA metabolic process
GO:0032200 / telomere organization
GO:0006886 / intracellular protein transport
GO:0045333 / cellular respiration
GO:0051168 / nuclear export
GO:0016236 / macroautophagy
GO:0071166 / ribonucleoprotein complex localization
GO:0006914 / autophagy
GO:0009894 / regulation of catabolic process
GO:0010608 / posttranscriptional regulation of gene expression
GO:0006406 / mRNA export from nucleus
GO:0071427 / mRNA-containing ribonucleoprotein complex export from ...
GO:0006405 / RNA export from nucleus
GO:0071426 / ribonucleoprotein complex export from nucleus
GO:0006414 / translational elongation
GO:1901566 / organonitrogen compound biosynthetic process
GO:0042254 / ribosome biogenesis
GO:0031329 / regulation of cellular catabolic process
GO:0065004 / protein-DNA complex assembly
GO:0031124 / mRNA 3'-end processing
GO:0006325 / chromatin organization
GO:0072594 / establishment of protein localization to organelle
GO:1901575 / organic substance catabolic process
GO:0016072 / rRNA metabolic process
GO:0006364 / rRNA processing
GO:0015031 / protein transport
GO:0006353 / DNA-templated transcription, termination
GO:0071824 / protein-DNA complex subunit organization
GO:0006401 / RNA catabolic process
GO:0033108 / mitochondrial respiratory chain complex assembly

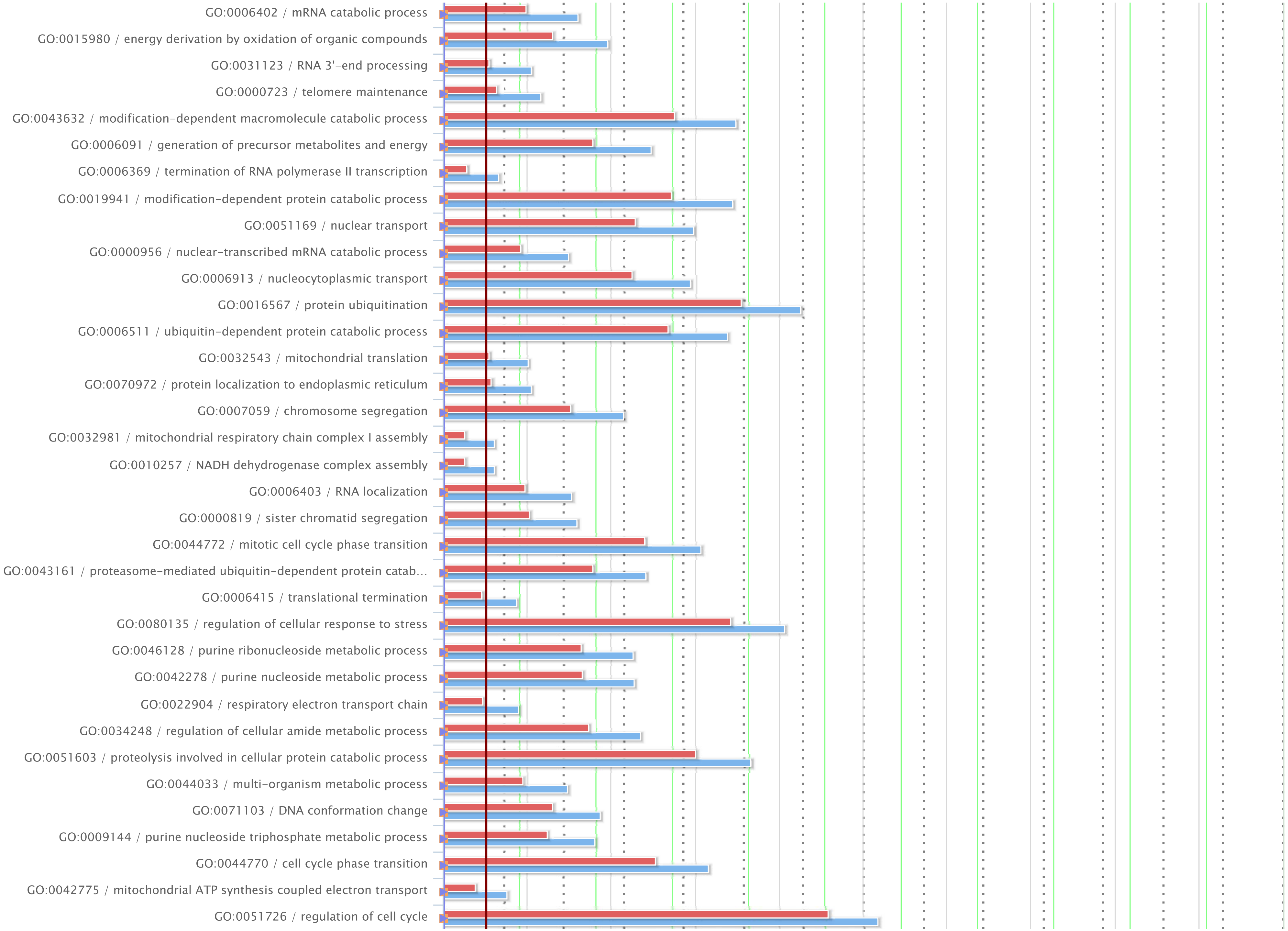
GO:0006402 / mRNA catabolic process
GO:0015980 / energy derivation by oxidation of organic compounds
GO:0031123 / RNA 3'-end processing
GO:0000723 / telomere maintenance
GO:0043632 / modification-dependent macromolecule catabolic process
GO:0006091 / generation of precursor metabolites and energy
GO:0006369 / termination of RNA polymerase II transcription
GO:0019941 / modification-dependent protein catabolic process
GO:0051169 / nuclear transport
GO:0000956 / nuclear-transcribed mRNA catabolic process
GO:0006913 / nucleocytoplasmic transport
GO:0016567 / protein ubiquitination
GO:0006511 / ubiquitin-dependent protein catabolic process
GO:0032543 / mitochondrial translation
GO:0070972 / protein localization to endoplasmic reticulum
GO:0007059 / chromosome segregation
GO:0032981 / mitochondrial respiratory chain complex I assembly
GO:0010257 / NADH dehydrogenase complex assembly
GO:0006403 / RNA localization
GO:0000819 / sister chromatid segregation
GO:0044772 / mitotic cell cycle phase transition
GO:0043161 / proteasome-mediated ubiquitin-dependent protein catab...
GO:0006415 / translational termination
GO:0080135 / regulation of cellular response to stress
GO:0046128 / purine ribonucleoside metabolic process
GO:0042278 / purine nucleoside metabolic process
GO:0022904 / respiratory electron transport chain
GO:0034248 / regulation of cellular amide metabolic process
GO:0051603 / proteolysis involved in cellular protein catabolic process
GO:0044033 / multi-organism metabolic process
GO:0071103 / DNA conformation change
GO:0009144 / purine nucleoside triphosphate metabolic process
GO:0044770 / cell cycle phase transition
GO:0042775 / mitochondrial ATP synthesis coupled electron transport
GO:0051726 / regulation of cell cycle

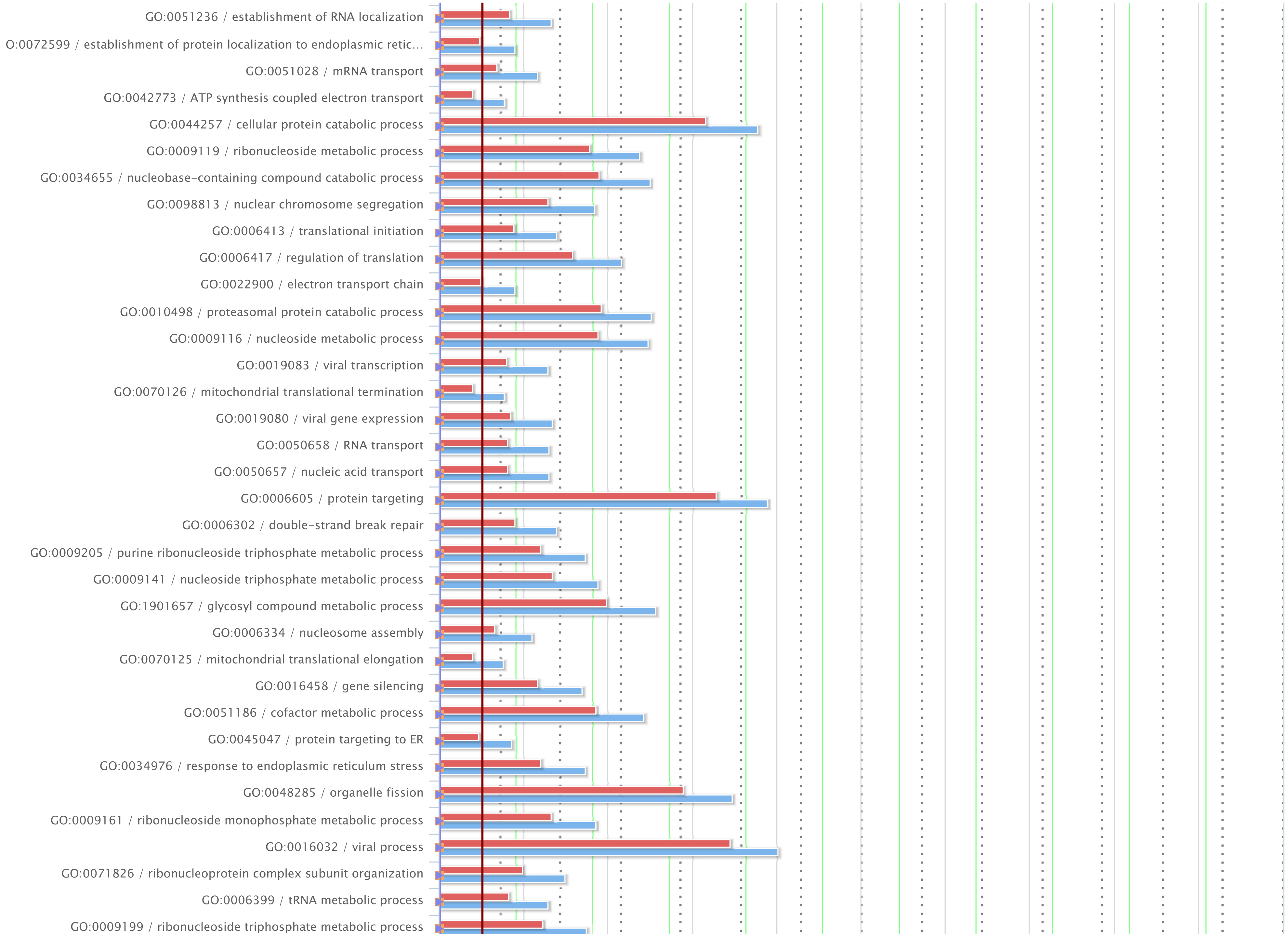
GO:0051236 / establishment of RNA localization
O:0072599 / establishment of protein localization to endoplasmic retic...
GO:0051028 / mRNA transport
GO:0042773 / ATP synthesis coupled electron transport
GO:0044257 / cellular protein catabolic process
GO:0009119 / ribonucleoside metabolic process
GO:0034655 / nucleobase-containing compound catabolic process
GO:0098813 / nuclear chromosome segregation
GO:0006413 / translational initiation
GO:0006417 / regulation of translation
GO:0022900 / electron transport chain
GO:0010498 / proteasomal protein catabolic process
GO:0009116 / nucleoside metabolic process
GO:0019083 / viral transcription
GO:0070126 / mitochondrial translational termination
GO:0019080 / viral gene expression
GO:0050658 / RNA transport
GO:0050657 / nucleic acid transport
GO:0006605 / protein targeting
GO:0006302 / double-strand break repair
GO:0009205 / purine ribonucleoside triphosphate metabolic process
GO:0009141 / nucleoside triphosphate metabolic process
GO:1901657 / glycosyl compound metabolic process
GO:0006334 / nucleosome assembly
GO:0070125 / mitochondrial translational elongation
GO:0016458 / gene silencing
GO:0051186 / cofactor metabolic process
GO:0045047 / protein targeting to ER
GO:0034976 / response to endoplasmic reticulum stress
GO:0048285 / organelle fission
GO:0009161 / ribonucleoside monophosphate metabolic process
GO:0016032 / viral process
GO:0071826 / ribonucleoprotein complex subunit organization
GO:0006399 / tRNA metabolic process
GO:0009199 / ribonucleoside triphosphate metabolic process

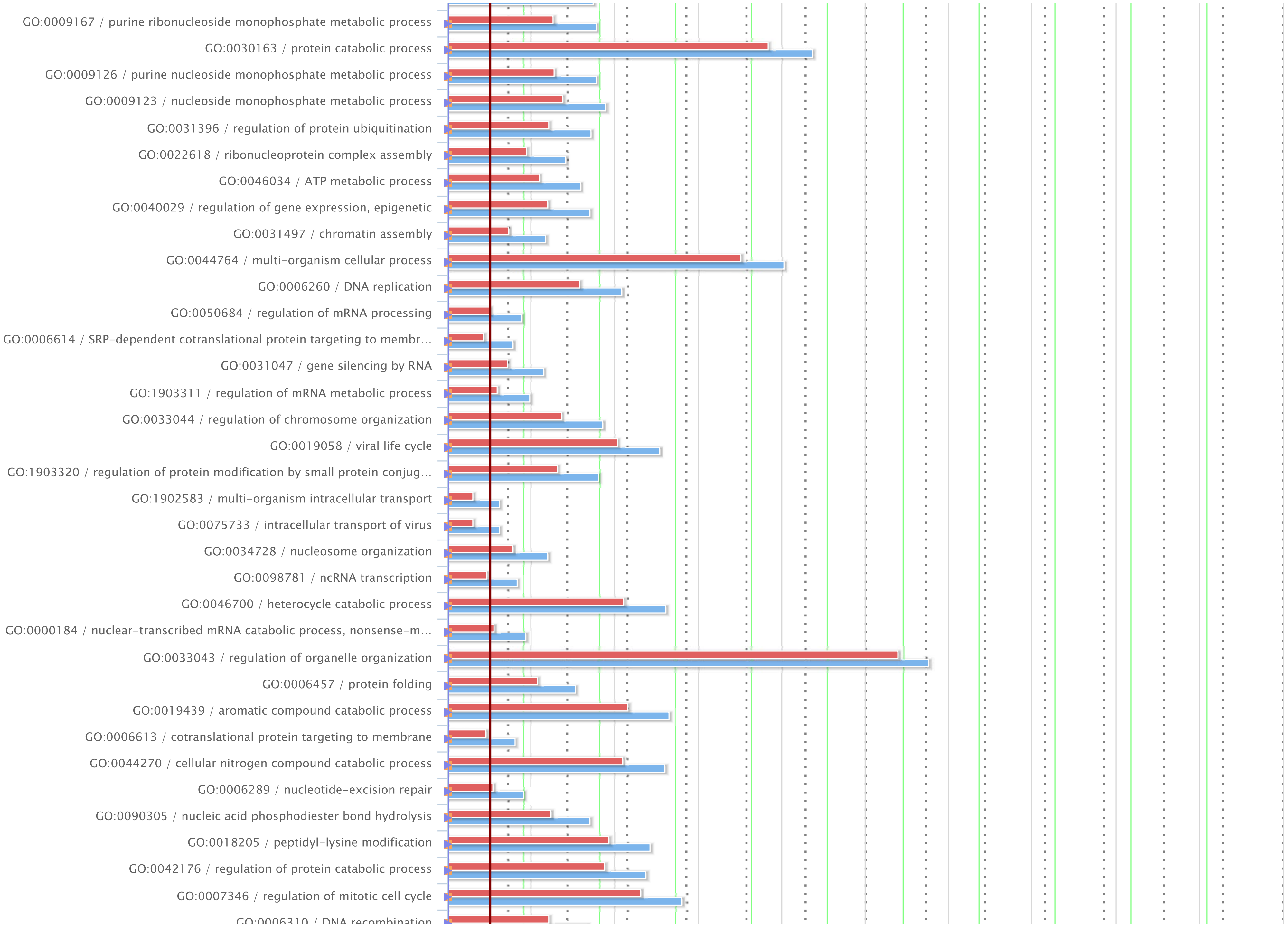
GO:0009167 / purine ribonucleoside monophosphate metabolic process
GO:0030163 / protein catabolic process
GO:0009126 / purine nucleoside monophosphate metabolic process
GO:0009123 / nucleoside monophosphate metabolic process
GO:0031396 / regulation of protein ubiquitination
GO:0022618 / ribonucleoprotein complex assembly
GO:0046034 / ATP metabolic process
GO:0040029 / regulation of gene expression, epigenetic
GO:0031497 / chromatin assembly
GO:0044764 / multi-organism cellular process
GO:0006260 / DNA replication
GO:0050684 / regulation of mRNA processing
GO:0006614 / SRP-dependent cotranslational protein targeting to membr...
GO:0031047 / gene silencing by RNA
GO:1903311 / regulation of mRNA metabolic process
GO:0033044 / regulation of chromosome organization
GO:0019058 / viral life cycle
GO:1903320 / regulation of protein modification by small protein conjug...
GO:1902583 / multi-organism intracellular transport
GO:0075733 / intracellular transport of virus
GO:0034728 / nucleosome organization
GO:0098781 / ncRNA transcription
GO:0046700 / heterocycle catabolic process
GO:0000184 / nuclear-transcribed mRNA catabolic process, nonsense-m...
GO:0033043 / regulation of organelle organization
GO:0006457 / protein folding
GO:0019439 / aromatic compound catabolic process
GO:0006613 / cotranslational protein targeting to membrane
GO:0044270 / cellular nitrogen compound catabolic process
GO:0006289 / nucleotide-excision repair
GO:0090305 / nucleic acid phosphodiester bond hydrolysis
GO:0018205 / peptidyl-lysine modification
GO:0042176 / regulation of protein catabolic process
GO:0007346 / regulation of mitotic cell cycle
GO:0006310 / DNA recombination

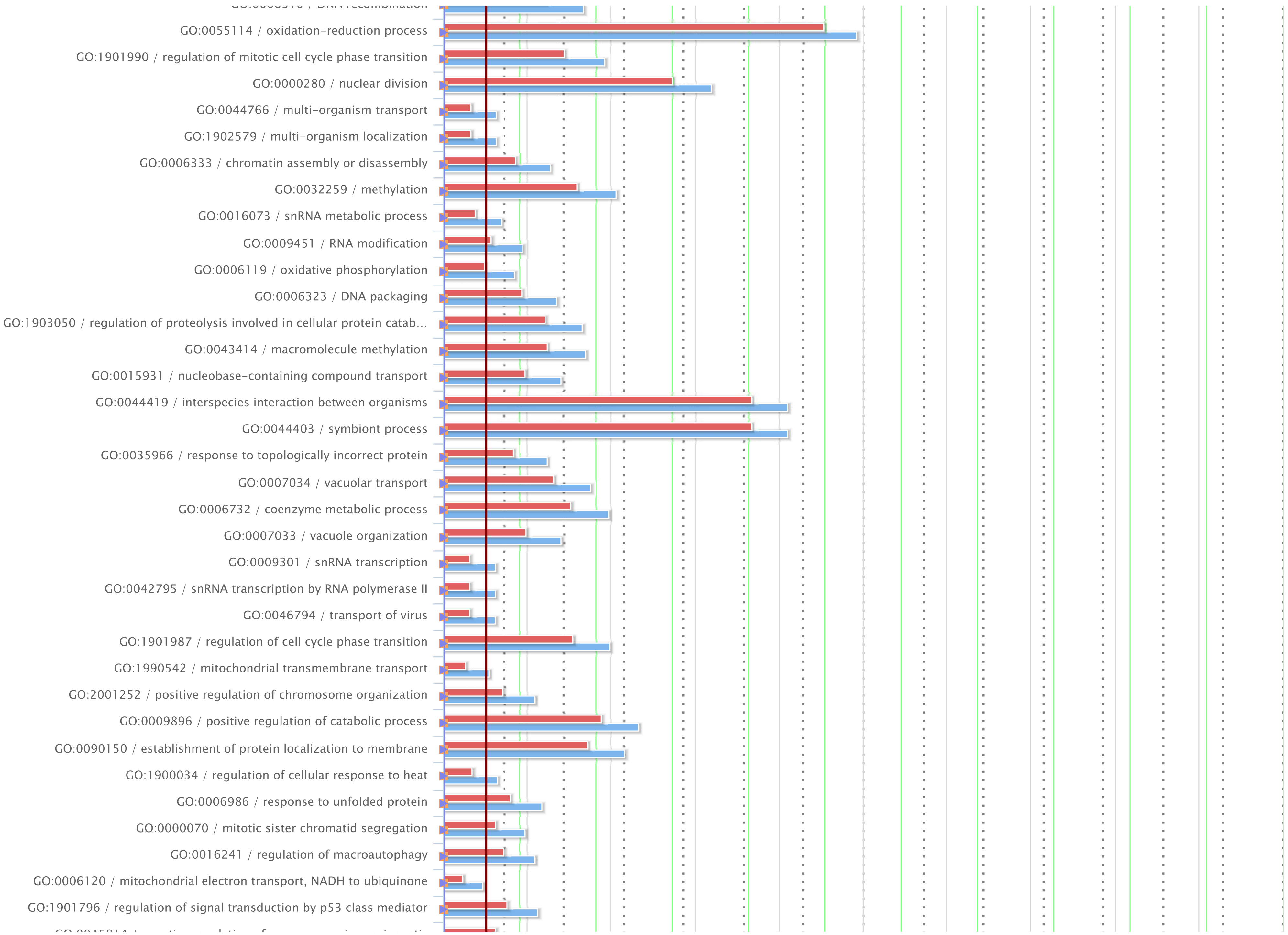
GO:0055114 / oxidation–reduction process
GO:1901990 / regulation of mitotic cell cycle phase transition
GO:0000280 / nuclear division
GO:0044766 / multi–organism transport
GO:1902579 / multi–organism localization
GO:0006333 / chromatin assembly or disassembly
GO:0032259 / methylation
GO:0016073 / snRNA metabolic process
GO:0009451 / RNA modification
GO:0006119 / oxidative phosphorylation
GO:0006323 / DNA packaging
GO:1903050 / regulation of proteolysis involved in cellular protein catab...
GO:0043414 / macromolecule methylation
GO:0015931 / nucleobase–containing compound transport
GO:0044419 / interspecies interaction between organisms
GO:0044403 / symbiont process
GO:0035966 / response to topologically incorrect protein
GO:0007034 / vacuolar transport
GO:0006732 / coenzyme metabolic process
GO:0007033 / vacuole organization
GO:0009301 / snRNA transcription
GO:0042795 / snRNA transcription by RNA polymerase II
GO:0046794 / transport of virus
GO:1901987 / regulation of cell cycle phase transition
GO:1990542 / mitochondrial transmembrane transport
GO:2001252 / positive regulation of chromosome organization
GO:0009896 / positive regulation of catabolic process
GO:0090150 / establishment of protein localization to membrane
GO:1900034 / regulation of cellular response to heat
GO:0006986 / response to unfolded protein
GO:0000070 / mitotic sister chromatid segregation
GO:0016241 / regulation of macroautophagy
GO:0006120 / mitochondrial electron transport, NADH to ubiquinone
GO:1901796 / regulation of signal transduction by p53 class mediator

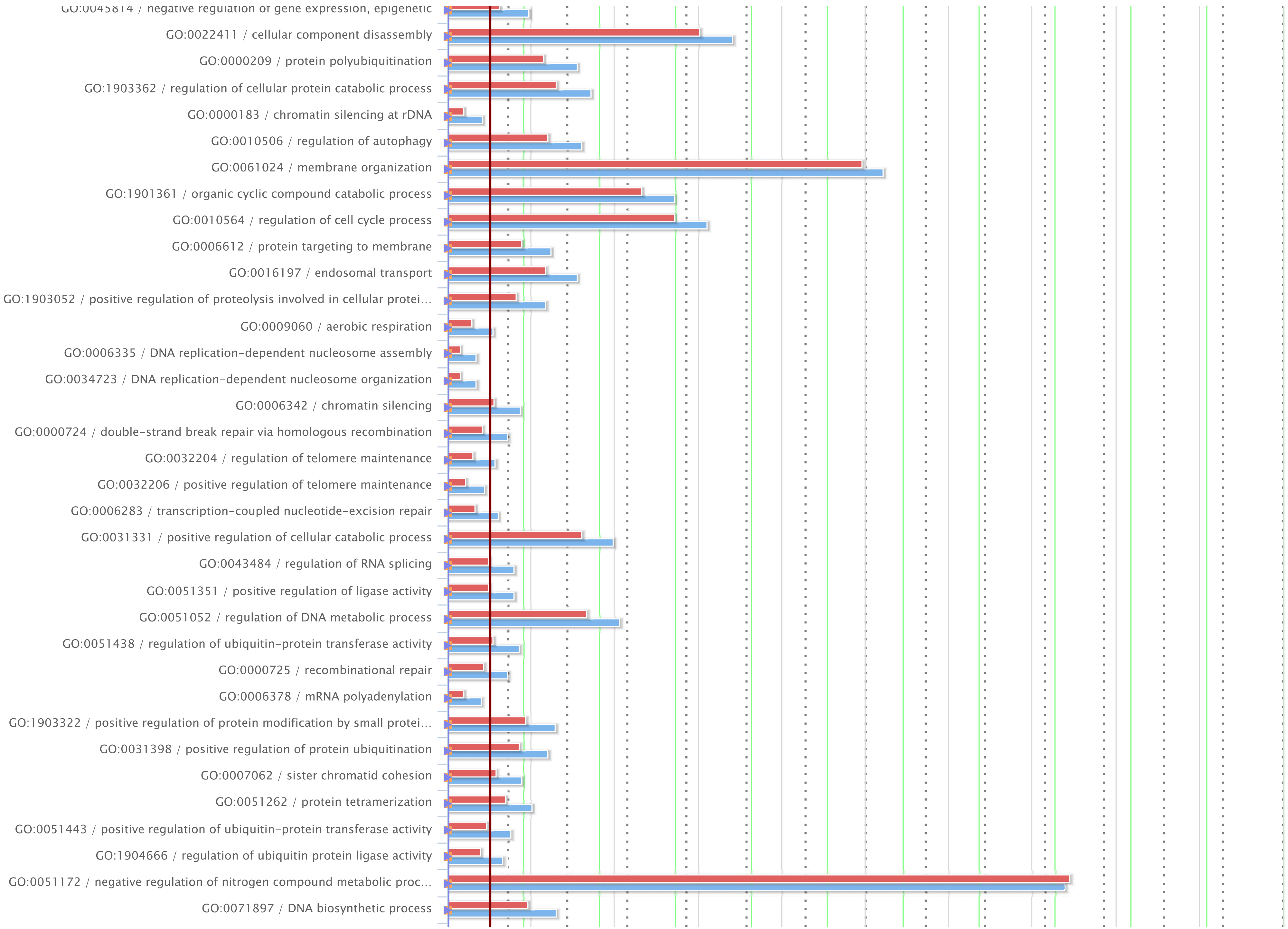
GO:0045814 / negative regulation of gene expression, epigenetic
GO:0022411 / cellular component disassembly
GO:0000209 / protein polyubiquitination
GO:1903362 / regulation of cellular protein catabolic process
GO:0000183 / chromatin silencing at rDNA
GO:0010506 / regulation of autophagy
GO:0061024 / membrane organization
GO:1901361 / organic cyclic compound catabolic process
GO:0010564 / regulation of cell cycle process
GO:0006612 / protein targeting to membrane
GO:0016197 / endosomal transport
GO:1903052 / positive regulation of proteolysis involved in cellular protei...
GO:0009060 / aerobic respiration
GO:0006335 / DNA replication-dependent nucleosome assembly
GO:0034723 / DNA replication-dependent nucleosome organization
GO:0006342 / chromatin silencing
GO:0000724 / double-strand break repair via homologous recombination
GO:0032204 / regulation of telomere maintenance
GO:0032206 / positive regulation of telomere maintenance
GO:0006283 / transcription-coupled nucleotide-excision repair
GO:0031331 / positive regulation of cellular catabolic process
GO:0043484 / regulation of RNA splicing
GO:0051351 / positive regulation of ligase activity
GO:0051052 / regulation of DNA metabolic process
GO:0051438 / regulation of ubiquitin-protein transferase activity
GO:0000725 / recombinational repair
GO:0006378 / mRNA polyadenylation
GO:1903322 / positive regulation of protein modification by small protei...
GO:0031398 / positive regulation of protein ubiquitination
GO:0007062 / sister chromatid cohesion
GO:0051262 / protein tetramerization
GO:0051443 / positive regulation of ubiquitin-protein transferase activity
GO:1904666 / regulation of ubiquitin protein ligase activity
GO:0051172 / negative regulation of nitrogen compound metabolic proc...
GO:0071897 / DNA biosynthetic process

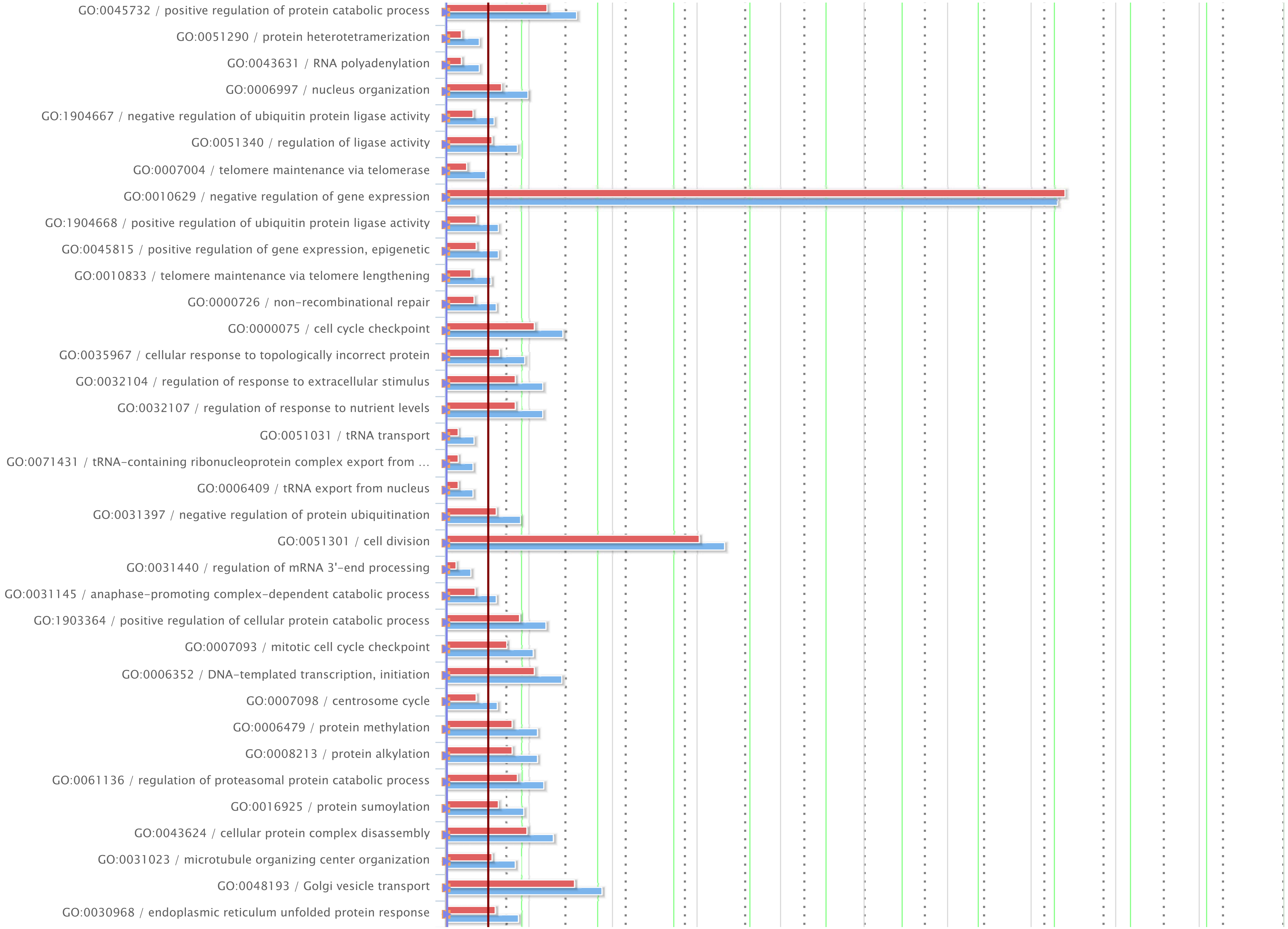
GO:0045732 / positive regulation of protein catabolic process
GO:0051290 / protein heterotetramerization
GO:0043631 / RNA polyadenylation
GO:0006997 / nucleus organization
GO:1904667 / negative regulation of ubiquitin protein ligase activity
GO:0051340 / regulation of ligase activity
GO:0007004 / telomere maintenance via telomerase
GO:0010629 / negative regulation of gene expression
GO:1904668 / positive regulation of ubiquitin protein ligase activity
GO:0045815 / positive regulation of gene expression, epigenetic
GO:0010833 / telomere maintenance via telomere lengthening
GO:0000726 / non−recombinational repair
GO:0000075 / cell cycle checkpoint
GO:0035967 / cellular response to topologically incorrect protein
GO:0032104 / regulation of response to extracellular stimulus
GO:0032107 / regulation of response to nutrient levels
GO:0051031 / tRNA transport
GO:0071431 / tRNA−containing ribonucleoprotein complex export from ...
GO:0006409 / tRNA export from nucleus
GO:0031397 / negative regulation of protein ubiquitination
GO:0051301 / cell division
GO:0031440 / regulation of mRNA 3'−end processing
GO:0031145 / anaphase−promoting complex−dependent catabolic process
GO:1903364 / positive regulation of cellular protein catabolic process
GO:0007093 / mitotic cell cycle checkpoint
GO:0006352 / DNA−templated transcription, initiation
GO:0007098 / centrosome cycle
GO:0006479 / protein methylation
GO:0008213 / protein alkylation
GO:0061136 / regulation of proteasomal protein catabolic process
GO:0016925 / protein sumoylation
GO:0043624 / cellular protein complex disassembly
GO:0031023 / microtubule organizing center organization
GO:0048193 / Golgi vesicle transport
GO:0030968 / endoplasmic reticulum unfolded protein response

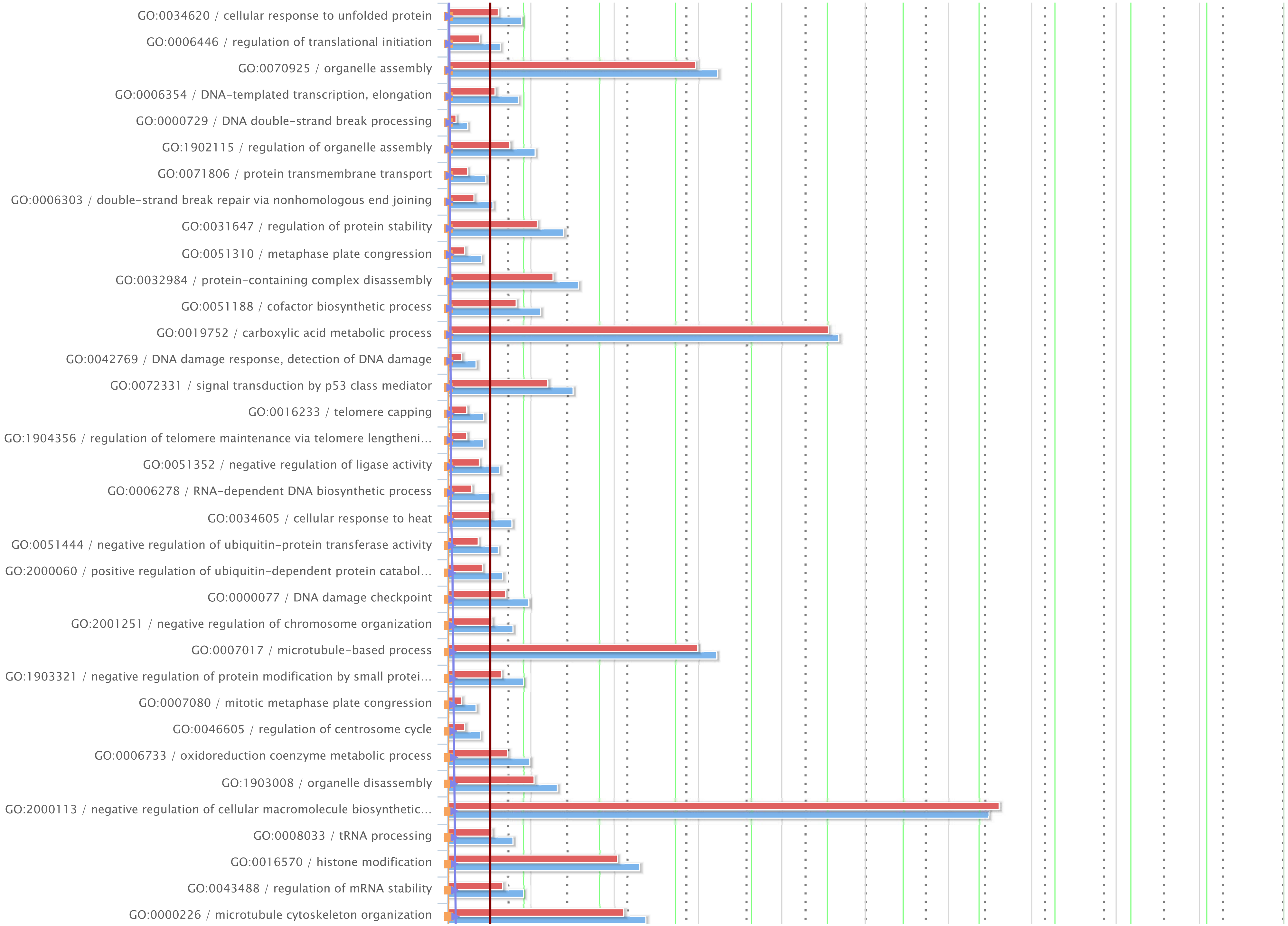
GO:0034620 / cellular response to unfolded protein
GO:0006446 / regulation of translational initiation
GO:0070925 / organelle assembly
GO:0006354 / DNA−templated transcription, elongation
GO:0000729 / DNA double−strand break processing
GO:1902115 / regulation of organelle assembly
GO:0071806 / protein transmembrane transport
GO:0006303 / double−strand break repair via nonhomologous end joining
GO:0031647 / regulation of protein stability
GO:0051310 / metaphase plate congression
GO:0032984 / protein−containing complex disassembly
GO:0051188 / cofactor biosynthetic process
GO:0019752 / carboxylic acid metabolic process
GO:0042769 / DNA damage response, detection of DNA damage
GO:0072331 / signal transduction by p53 class mediator
GO:0016233 / telomere capping
GO:1904356 / regulation of telomere maintenance via telomere lengtheni...
GO:0051352 / negative regulation of ligase activity
GO:0006278 / RNA−dependent DNA biosynthetic process
GO:0034605 / cellular response to heat
GO:0051444 / negative regulation of ubiquitin−protein transferase activity
GO:2000060 / positive regulation of ubiquitin−dependent protein catabol...
GO:0000077 / DNA damage checkpoint
GO:2001251 / negative regulation of chromosome organization
GO:0007017 / microtubule−based process
GO:1903321 / negative regulation of protein modification by small protei...
GO:0007080 / mitotic metaphase plate congression
GO:0046605 / regulation of centrosome cycle
GO:0006733 / oxidoreduction coenzyme metabolic process
GO:1903008 / organelle disassembly
GO:2000113 / negative regulation of cellular macromolecule biosynthetic...
GO:0008033 / tRNA processing
GO:0016570 / histone modification
GO:0043488 / regulation of mRNA stability
GO:0000226 / microtubule cytoskeleton organization

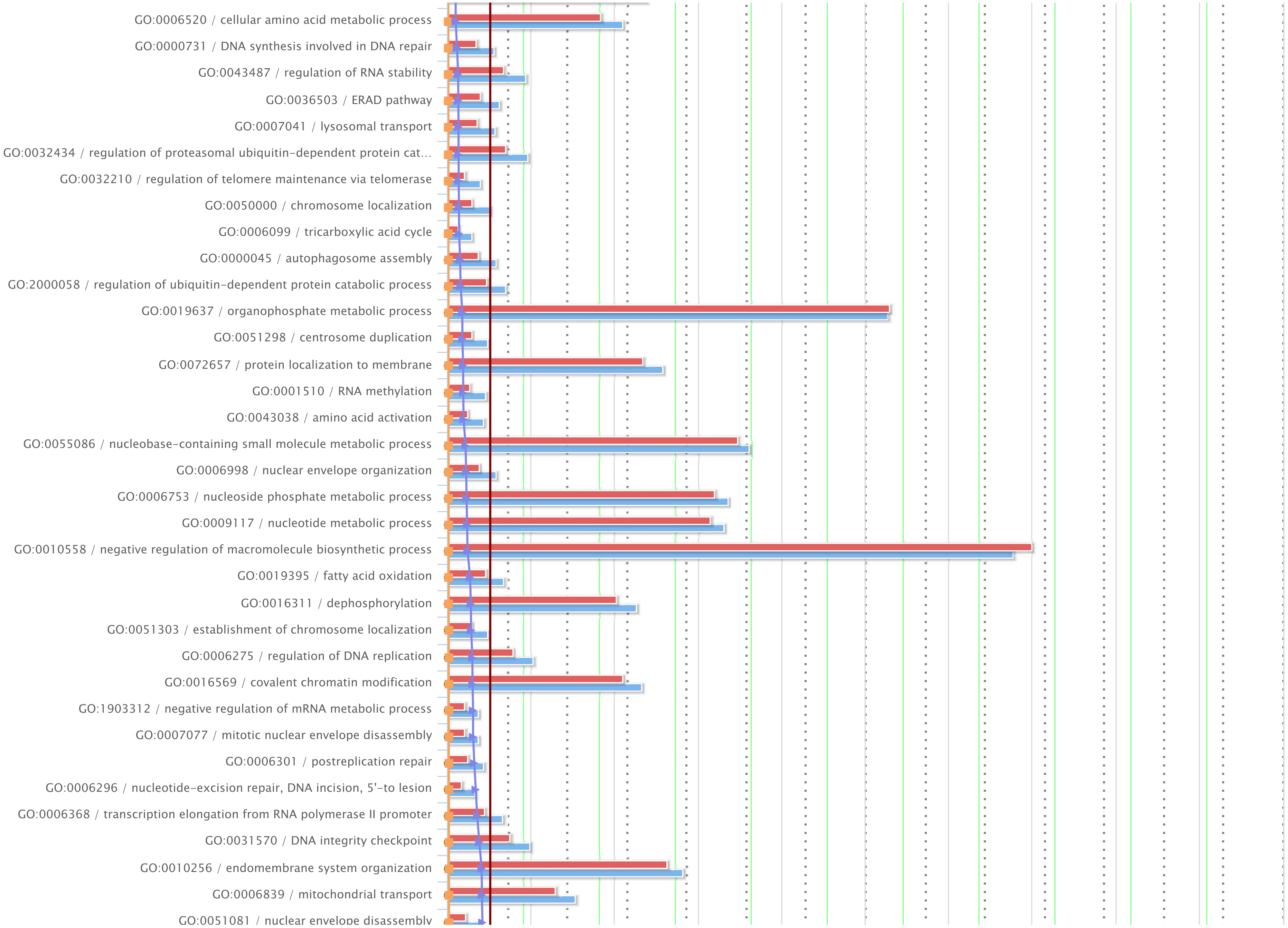
GO:0006520 / cellular amino acid metabolic process
GO:0000731 / DNA synthesis involved in DNA repair
GO:0043487 / regulation of RNA stability
GO:0036503 / ERAD pathway
GO:0007041 / lysosomal transport
GO:0032434 / regulation of proteasomal ubiquitin-dependent protein cat...
GO:0032210 / regulation of telomere maintenance via telomerase
GO:0050000 / chromosome localization
GO:0006099 / tricarboxylic acid cycle
GO:0000045 / autophagosome assembly
GO:2000058 / regulation of ubiquitin-dependent protein catabolic process
GO:0019637 / organophosphate metabolic process
GO:0051298 / centrosome duplication
GO:0072657 / protein localization to membrane
GO:0001510 / RNA methylation
GO:0043038 / amino acid activation
GO:0055086 / nucleobase-containing small molecule metabolic process
GO:0006998 / nuclear envelope organization
GO:0006753 / nucleoside phosphate metabolic process
GO:0009117 / nucleotide metabolic process
GO:0010558 / negative regulation of macromolecule biosynthetic process
GO:0019395 / fatty acid oxidation
GO:0016311 / dephosphorylation
GO:0051303 / establishment of chromosome localization
GO:0006275 / regulation of DNA replication
GO:0016569 / covalent chromatin modification
GO:1903312 / negative regulation of mRNA metabolic process
GO:0007077 / mitotic nuclear envelope disassembly
GO:0006301 / postreplication repair
GO:0006296 / nucleotide-excision repair, DNA incision, 5'-to lesion
GO:0006368 / transcription elongation from RNA polymerase II promoter
GO:0031570 / DNA integrity checkpoint
GO:0010256 / endomembrane system organization
GO:0006839 / mitochondrial transport
GO:0051081 / nuclear envelope disassembly

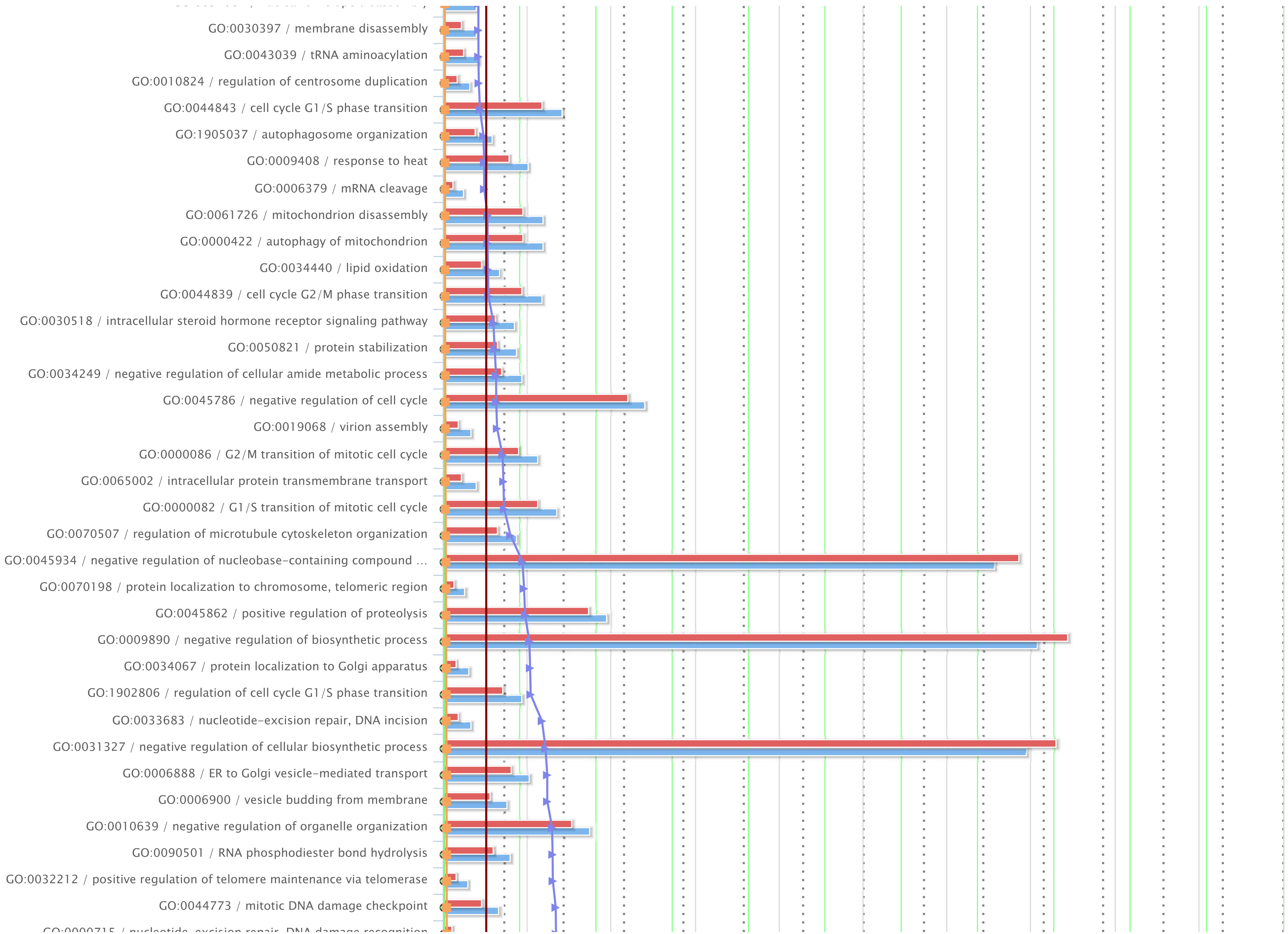
GO:0030397 / membrane disassembly
GO:0043039 / tRNA aminoacylation
GO:0010824 / regulation of centrosome duplication
GO:0044843 / cell cycle G1/S phase transition
GO:1905037 / autophagosome organization
GO:0009408 / response to heat
GO:0006379 / mRNA cleavage
GO:0061726 / mitochondrion disassembly
GO:0000422 / autophagy of mitochondrion
GO:0034440 / lipid oxidation
GO:0044839 / cell cycle G2/M phase transition
GO:0030518 / intracellular steroid hormone receptor signaling pathway
GO:0050821 / protein stabilization
GO:0034249 / negative regulation of cellular amide metabolic process
GO:0045786 / negative regulation of cell cycle
GO:0019068 / virion assembly
GO:0000086 / G2/M transition of mitotic cell cycle
GO:0065002 / intracellular protein transmembrane transport
GO:0000082 / G1/S transition of mitotic cell cycle
GO:0070507 / regulation of microtubule cytoskeleton organization
GO:0045934 / negative regulation of nucleobase-containing compound ...
GO:0070198 / protein localization to chromosome, telomeric region
GO:0045862 / positive regulation of proteolysis
GO:0009890 / negative regulation of biosynthetic process
GO:0034067 / protein localization to Golgi apparatus
GO:1902806 / regulation of cell cycle G1/S phase transition
GO:0033683 / nucleotide-excision repair, DNA incision
GO:0031327 / negative regulation of cellular biosynthetic process
GO:0006888 / ER to Golgi vesicle-mediated transport
GO:0006900 / vesicle budding from membrane
GO:0010639 / negative regulation of organelle organization
GO:0090501 / RNA phosphodiester bond hydrolysis
GO:0032212 / positive regulation of telomere maintenance via telomerase
GO:0044773 / mitotic DNA damage checkpoint

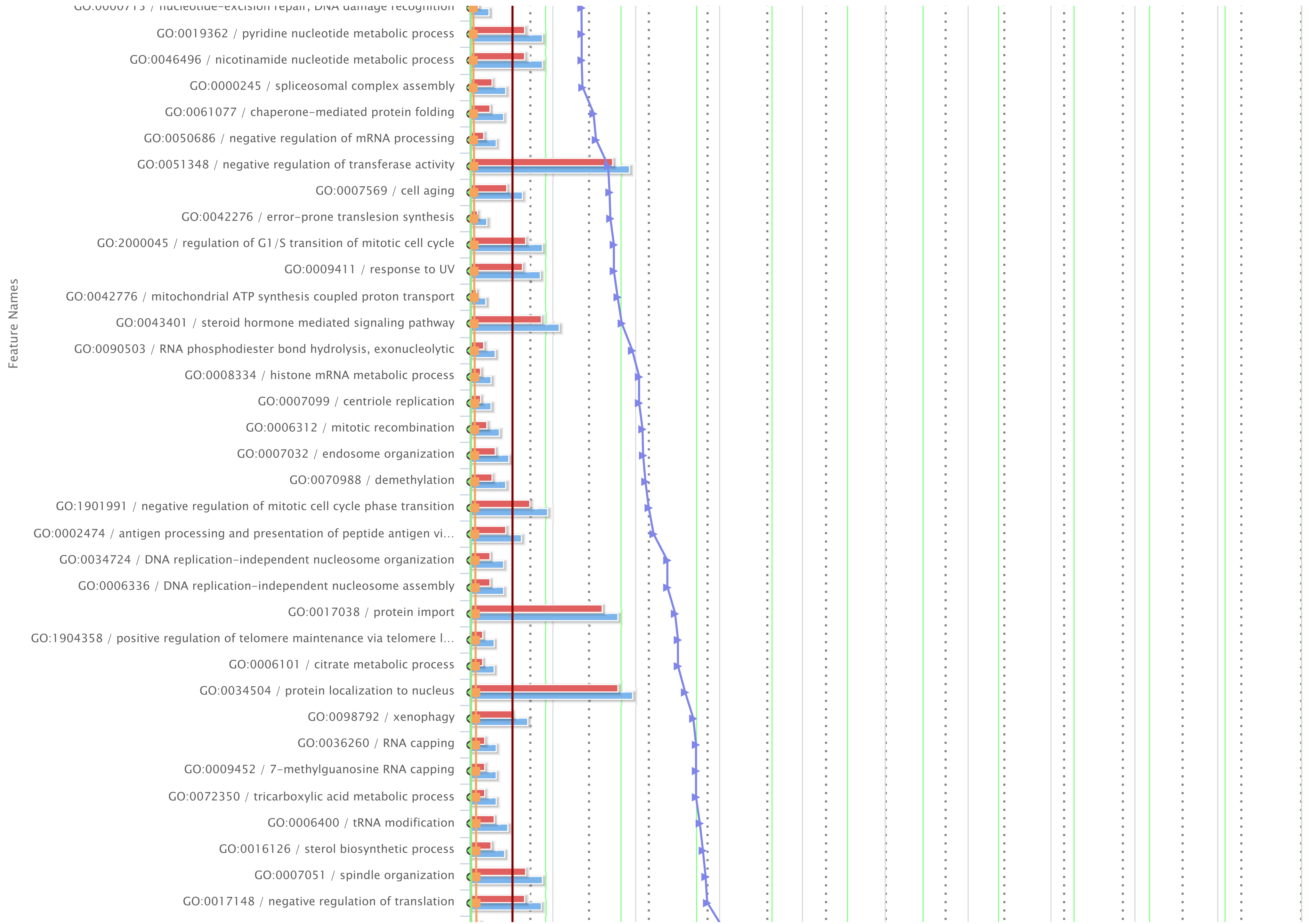
Feature Names
GO:0019362 / pyridine nucleotide metabolic process
GO:0046496 / nicotinamide nucleotide metabolic process
GO:0000245 / spliceosomal complex assembly
GO:0061077 / chaperone−mediated protein folding
GO:0050686 / negative regulation of mRNA processing
GO:0051348 / negative regulation of transferase activity
GO:0007569 / cell aging
GO:0042276 / error−prone translesion synthesis
GO:2000045 / regulation of G1/S transition of mitotic cell cycle
GO:0009411 / response to UV
GO:0042776 / mitochondrial ATP synthesis coupled proton transport
GO:0043401 / steroid hormone mediated signaling pathway
GO:0090503 / RNA phosphodiester bond hydrolysis, exonucleolytic
GO:0008334 / histone mRNA metabolic process
GO:0007099 / centriole replication
GO:0006312 / mitotic recombination
GO:0007032 / endosome organization
GO:0070988 / demethylation
GO:1901991 / negative regulation of mitotic cell cycle phase transition
GO:0002474 / antigen processing and presentation of peptide antigen vi...
GO:0034724 / DNA replication−independent nucleosome organization
GO:0006336 / DNA replication−independent nucleosome assembly
GO:0017038 / protein import
GO:1904358 / positive regulation of telomere maintenance via telomere l...
GO:0006101 / citrate metabolic process
GO:0034504 / protein localization to nucleus
GO:0098792 / xenophagy
GO:0036260 / RNA capping
GO:0009452 / 7−methylguanosine RNA capping
GO:0072350 / tricarboxylic acid metabolic process
GO:0006400 / tRNA modification
GO:0016126 / sterol biosynthetic process
GO:0007051 / spindle organization
GO:0017148 / negative regulation of translation

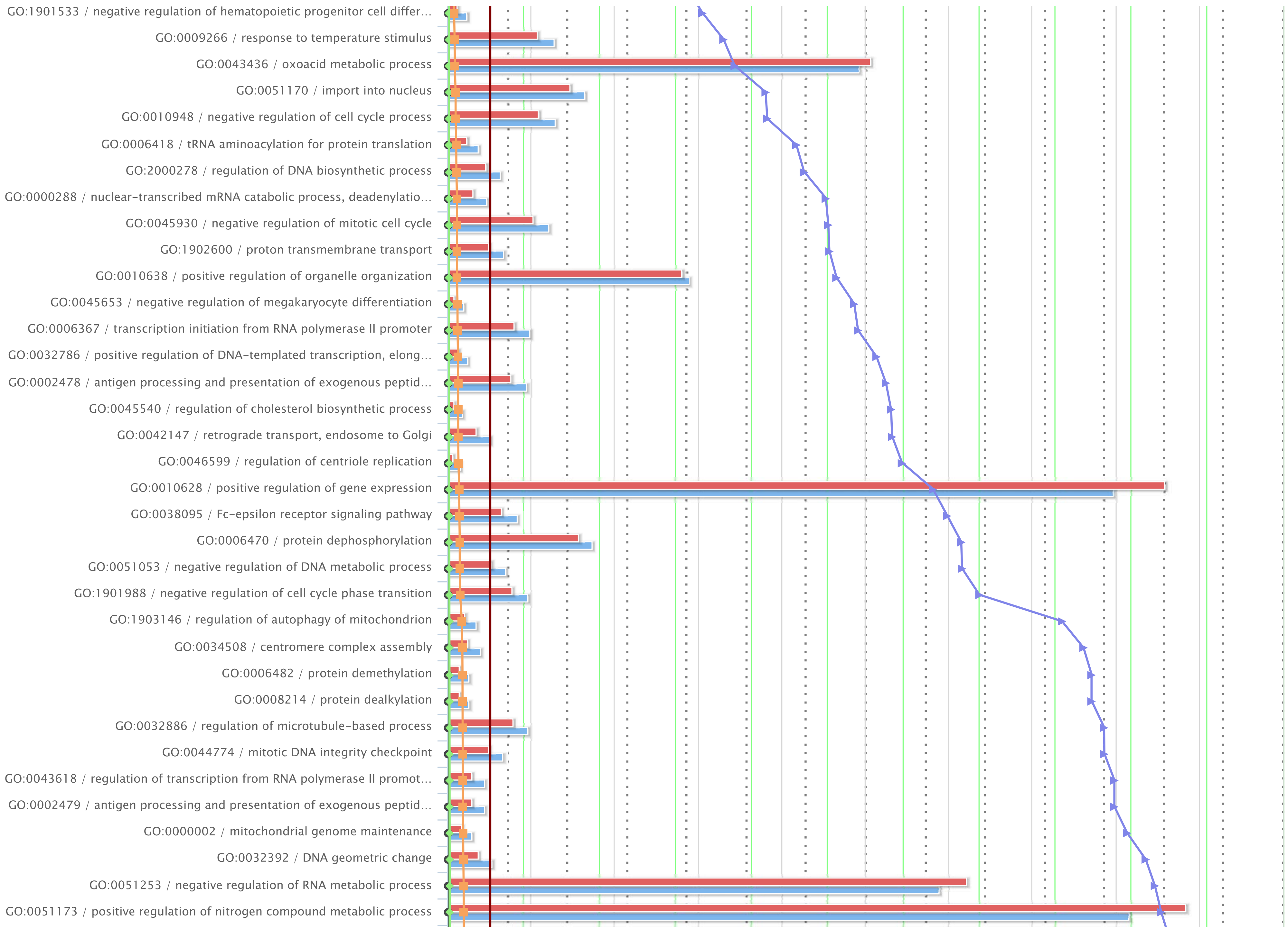

GO:1901533 / negative regulation of hematopoietic progenitor cell differ...
GO:0009266 / response to temperature stimulus
GO:0043436 / oxoacid metabolic process
GO:0051170 / import into nucleus
GO:0010948 / negative regulation of cell cycle process
GO:0006418 / tRNA aminoacylation for protein translation
GO:2000278 / regulation of DNA biosynthetic process
GO:0000288 / nuclear-transcribed mRNA catabolic process, deadenylatio...
GO:0045930 / negative regulation of mitotic cell cycle
GO:1902600 / proton transmembrane transport
GO:0010638 / positive regulation of organelle organization
GO:0045653 / negative regulation of megakaryocyte differentiation
GO:0006367 / transcription initiation from RNA polymerase II promoter
GO:0032786 / positive regulation of DNA-templated transcription, elong...
GO:0002478 / antigen processing and presentation of exogenous peptid...
GO:0045540 / regulation of cholesterol biosynthetic process
GO:0042147 / retrograde transport, endosome to Golgi
GO:0046599 / regulation of centriole replication
GO:0010628 / positive regulation of gene expression
GO:0038095 / Fc-epsilon receptor signaling pathway
GO:0006470 / protein dephosphorylation
GO:0051053 / negative regulation of DNA metabolic process
GO:1901988 / negative regulation of cell cycle phase transition
GO:1903146 / regulation of autophagy of mitochondrion
GO:0034508 / centromere complex assembly
GO:0006482 / protein demethylation
GO:0008214 / protein dealkylation
GO:0032886 / regulation of microtubule-based process
GO:0044774 / mitotic DNA integrity checkpoint
GO:0043618 / regulation of transcription from RNA polymerase II promot...
GO:0002479 / antigen processing and presentation of exogenous peptid...
GO:0000002 / mitochondrial genome maintenance
GO:0032392 / DNA geometric change
GO:0051253 / negative regulation of RNA metabolic process
GO:0051173 / positive regulation of nitrogen compound metabolic process

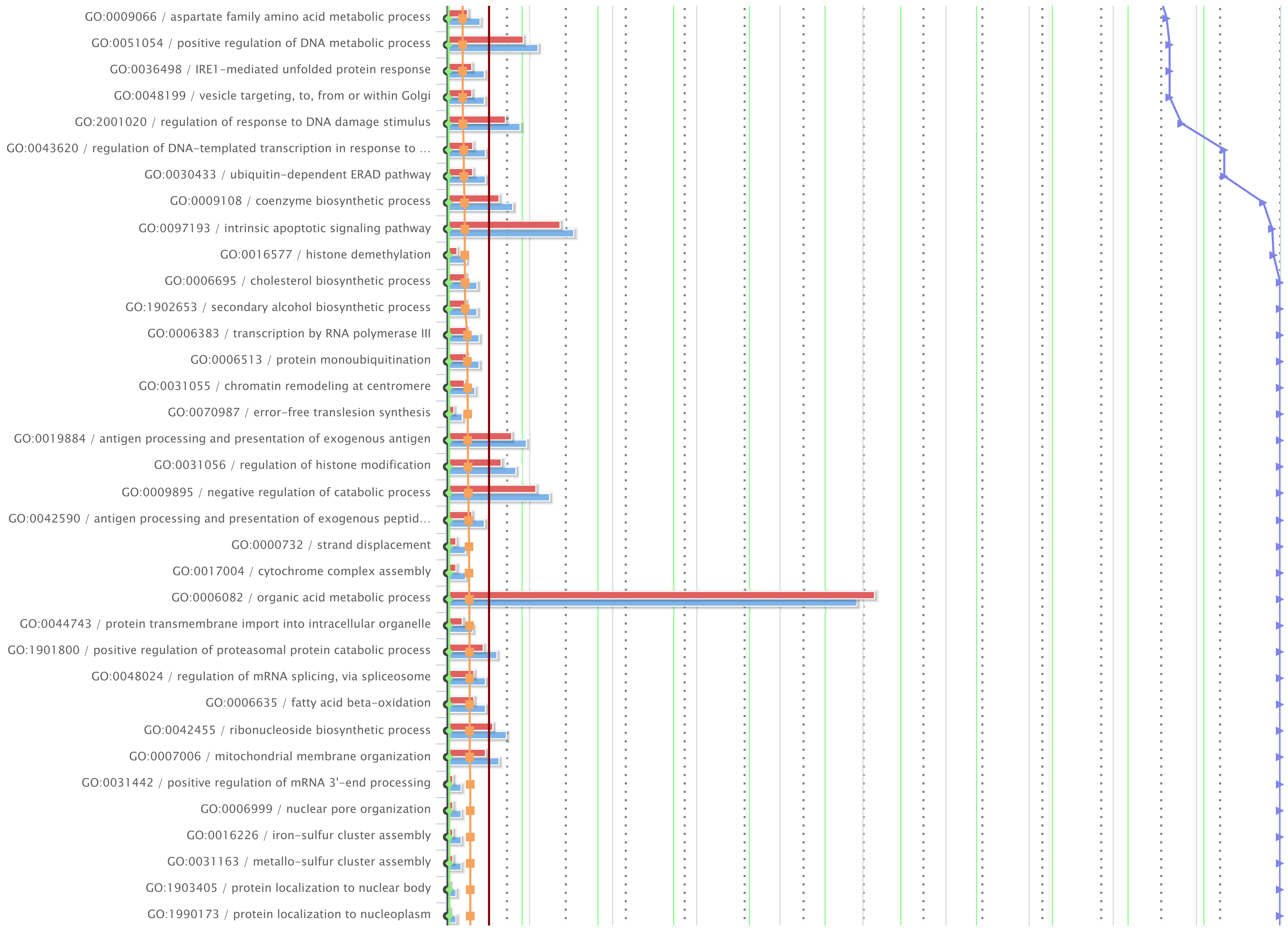
GO:0009066 / aspartate family amino acid metabolic process
GO:0051054 / positive regulation of DNA metabolic process
GO:0036498 / IRE1−mediated unfolded protein response
GO:0048199 / vesicle targeting, to, from or within Golgi
GO:2001020 / regulation of response to DNA damage stimulus
GO:0043620 / regulation of DNA−templated transcription in response to ...
GO:0030433 / ubiquitin−dependent ERAD pathway
GO:0009108 / coenzyme biosynthetic process
GO:0097193 / intrinsic apoptotic signaling pathway
GO:0016577 / histone demethylation
GO:0006695 / cholesterol biosynthetic process
GO:1902653 / secondary alcohol biosynthetic process
GO:0006383 / transcription by RNA polymerase III
GO:0006513 / protein monoubiquitination
GO:0031055 / chromatin remodeling at centromere
GO:0070987 / error−free translesion synthesis
GO:0019884 / antigen processing and presentation of exogenous antigen
GO:0031056 / regulation of histone modification
GO:0009895 / negative regulation of catabolic process
GO:0042590 / antigen processing and presentation of exogenous peptid...
GO:0000732 / strand displacement
GO:0017004 / cytochrome complex assembly
GO:0006082 / organic acid metabolic process
GO:0044743 / protein transmembrane import into intracellular organelle
GO:1901800 / positive regulation of proteasomal protein catabolic process
GO:0048024 / regulation of mRNA splicing, via spliceosome
GO:0006635 / fatty acid beta−oxidation
GO:0042455 / ribonucleoside biosynthetic process
GO:0007006 / mitochondrial membrane organization
GO:0031442 / positive regulation of mRNA 3'−end processing
GO:0006999 / nuclear pore organization
GO:0016226 / iron−sulfur cluster assembly
GO:0031163 / metallo−sulfur cluster assembly
GO:1903405 / protein localization to nuclear body
GO:1990173 / protein localization to nucleoplasm

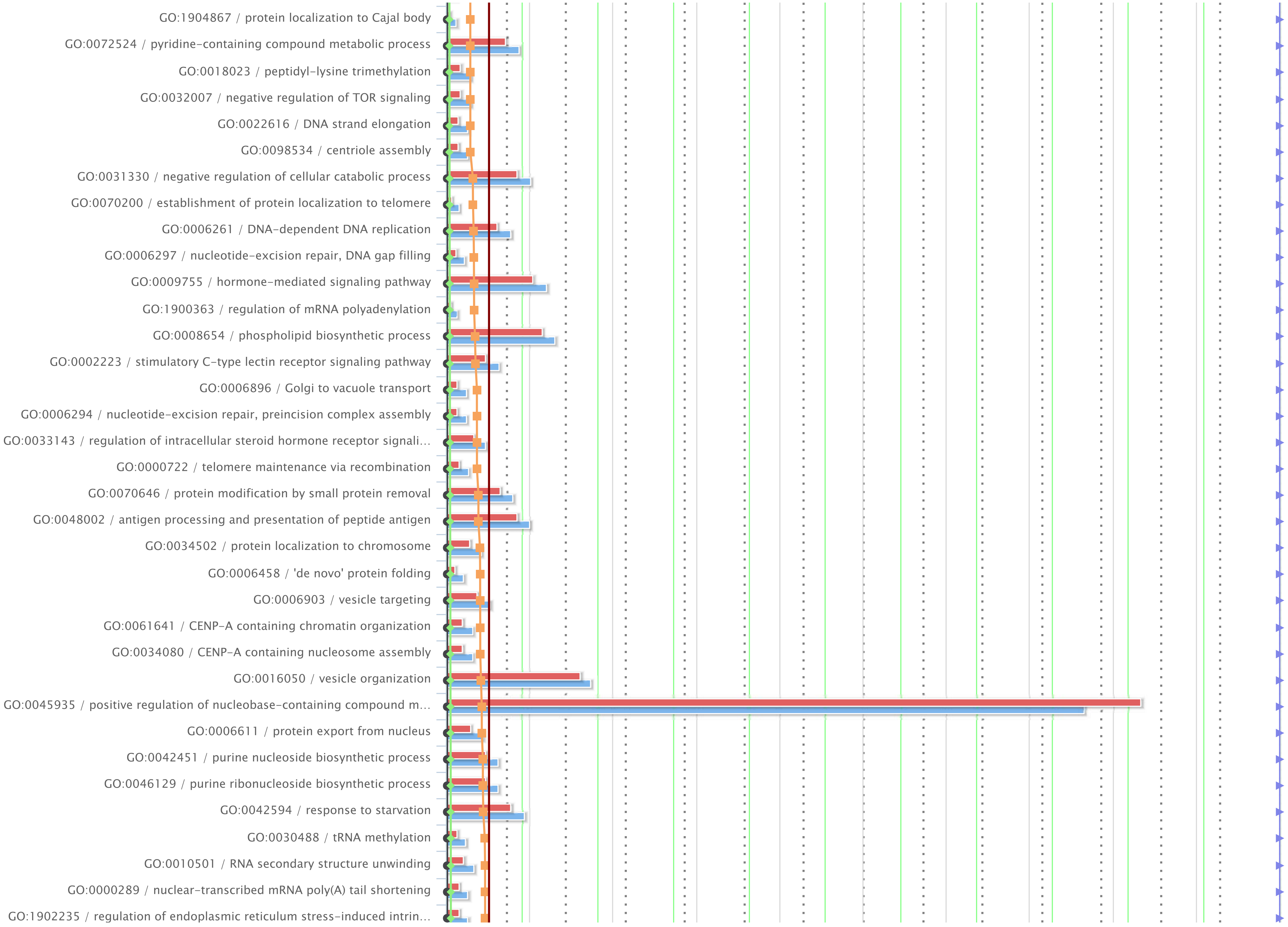

GO:1904867 / protein localization to Cajal body
GO:0072524 / pyridine-containing compound metabolic process
GO:0018023 / peptidyl-lysine trimethylation
GO:0032007 / negative regulation of TOR signaling
GO:0022616 / DNA strand elongation
GO:0098534 / centriole assembly
GO:0031330 / negative regulation of cellular catabolic process
GO:0070200 / establishment of protein localization to telomere
GO:0006261 / DNA-dependent DNA replication
GO:0006297 / nucleotide-excision repair, DNA gap filling
GO:0009755 / hormone-mediated signaling pathway
GO:1900363 / regulation of mRNA polyadenylation
GO:0008654 / phospholipid biosynthetic process
GO:0002223 / stimulatory C-type lectin receptor signaling pathway
GO:0006896 / Golgi to vacuole transport
GO:0006294 / nucleotide-excision repair, preincision complex assembly
GO:0033143 / regulation of intracellular steroid hormone receptor signali...
GO:0000722 / telomere maintenance via recombination
GO:0070646 / protein modification by small protein removal
GO:0048002 / antigen processing and presentation of peptide antigen
GO:0034502 / protein localization to chromosome
GO:0006458 / 'de novo' protein folding
GO:0006903 / vesicle targeting
GO:0061641 / CENP-A containing chromatin organization
GO:0034080 / CENP-A containing nucleosome assembly
GO:0016050 / vesicle organization
GO:0045935 / positive regulation of nucleobase-containing compound m...
GO:0006611 / protein export from nucleus
GO:0042451 / purine nucleoside biosynthetic process
GO:0046129 / purine ribonucleoside biosynthetic process
GO:0042594 / response to starvation
GO:0030488 / tRNA methylation
GO:0010501 / RNA secondary structure unwinding
GO:0000289 / nuclear-transcribed mRNA poly(A) tail shortening
GO:1902235 / regulation of endoplasmic reticulum stress-induced intrin...

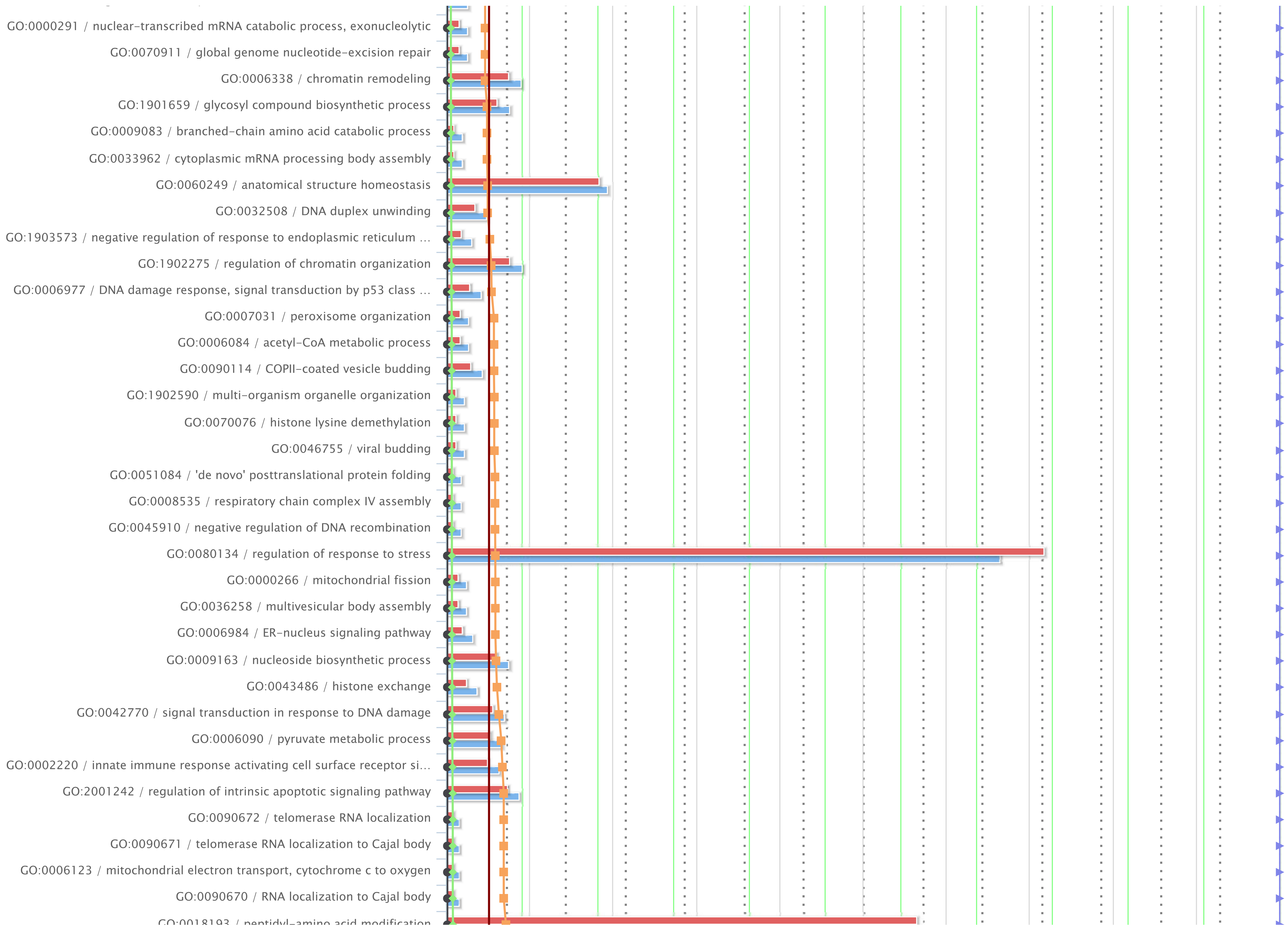
GO:0000291 / nuclear–transcribed mRNA catabolic process, exonucleolytic
GO:0070911 / global genome nucleotide–excision repair
GO:0006338 / chromatin remodeling
GO:1901659 / glycosyl compound biosynthetic process
GO:0009083 / branched–chain amino acid catabolic process
GO:0033962 / cytoplasmic mRNA processing body assembly
GO:0060249 / anatomical structure homeostasis
GO:0032508 / DNA duplex unwinding
GO:1903573 / negative regulation of response to endoplasmic reticulum ...
GO:1902275 / regulation of chromatin organization
GO:0006977 / DNA damage response, signal transduction by p53 class ...
GO:0007031 / peroxisome organization
GO:0006084 / acetyl–CoA metabolic process
GO:0090114 / COPII–coated vesicle budding
GO:1902590 / multi–organism organelle organization
GO:0070076 / histone lysine demethylation
GO:0046755 / viral budding
GO:0051084 / 'de novo' posttranslational protein folding
GO:0008535 / respiratory chain complex IV assembly
GO:0045910 / negative regulation of DNA recombination
GO:0080134 / regulation of response to stress
GO:0000266 / mitochondrial fission
GO:0036258 / multivesicular body assembly
GO:0006984 / ER–nucleus signaling pathway
GO:0009163 / nucleoside biosynthetic process
GO:0043486 / histone exchange
GO:0042770 / signal transduction in response to DNA damage
GO:0006090 / pyruvate metabolic process
GO:0002220 / innate immune response activating cell surface receptor si...
GO:2001242 / regulation of intrinsic apoptotic signaling pathway
GO:0090672 / telomerase RNA localization
GO:0090671 / telomerase RNA localization to Cajal body
GO:0006123 / mitochondrial electron transport, cytochrome c to oxygen
GO:0090670 / RNA localization to Cajal body
GO:0018193 / peptidyl–amino acid modification

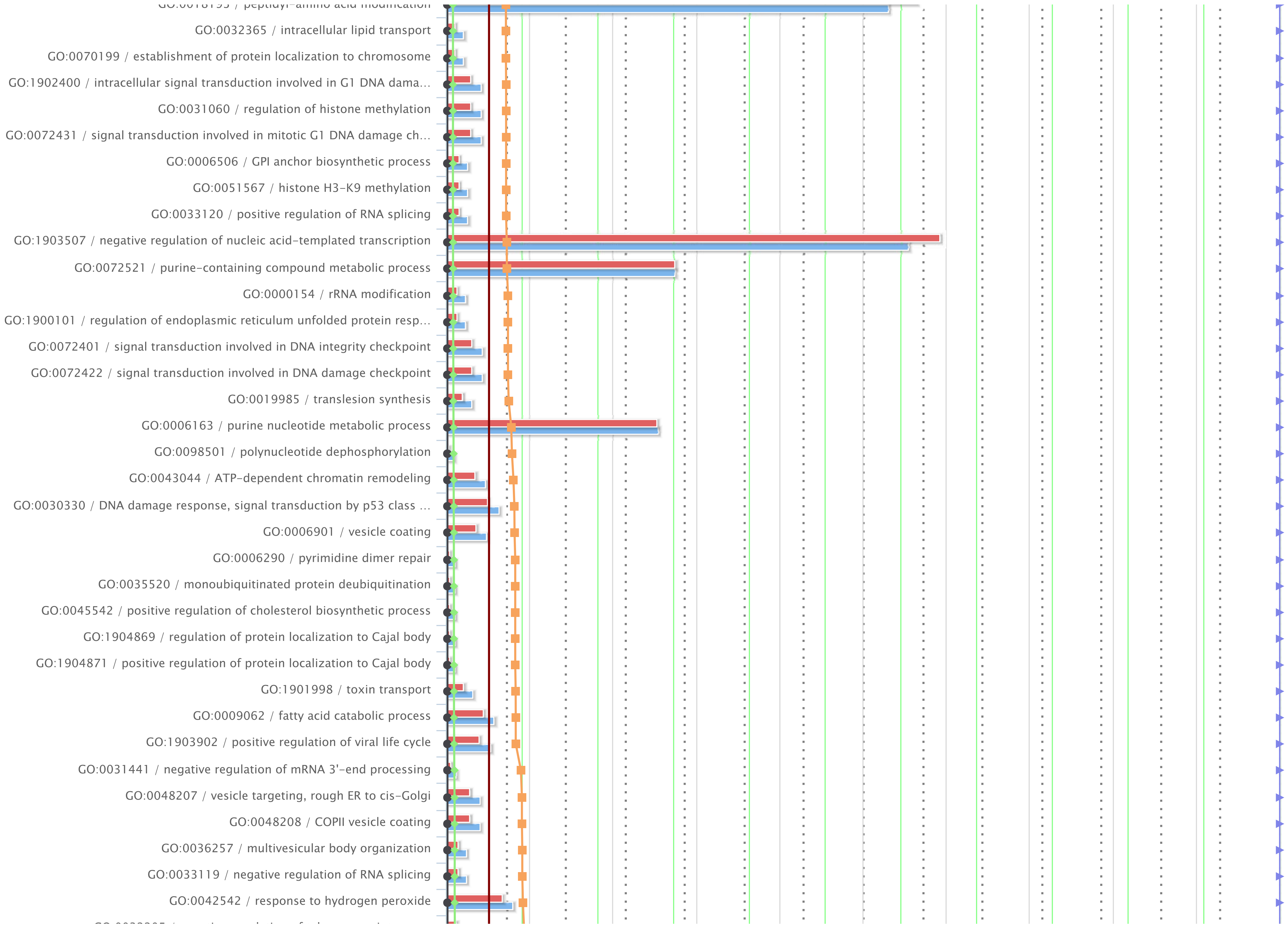

GO:0032365 / intracellular lipid transport
GO:0070199 / establishment of protein localization to chromosome
GO:1902400 / intracellular signal transduction involved in G1 DNA dama...
GO:0031060 / regulation of histone methylation
GO:0072431 / signal transduction involved in mitotic G1 DNA damage ch...
GO:0006506 / GPI anchor biosynthetic process
GO:0051567 / histone H3−K9 methylation
GO:0033120 / positive regulation of RNA splicing
GO:1903507 / negative regulation of nucleic acid−templated transcription
GO:0072521 / purine−containing compound metabolic process
GO:0000154 / rRNA modification
GO:1900101 / regulation of endoplasmic reticulum unfolded protein resp...
GO:0072401 / signal transduction involved in DNA integrity checkpoint
GO:0072422 / signal transduction involved in DNA damage checkpoint
GO:0019985 / translesion synthesis
GO:0006163 / purine nucleotide metabolic process
GO:0098501 / polynucleotide dephosphorylation
GO:0043044 / ATP−dependent chromatin remodeling
GO:0030330 / DNA damage response, signal transduction by p53 class ...
GO:0006901 / vesicle coating
GO:0006290 / pyrimidine dimer repair
GO:0035520 / monoubiquitinated protein deubiquitination
GO:0045542 / positive regulation of cholesterol biosynthetic process
GO:1904869 / regulation of protein localization to Cajal body
GO:1904871 / positive regulation of protein localization to Cajal body
GO:1901998 / toxin transport
GO:0009062 / fatty acid catabolic process
GO:1903902 / positive regulation of viral life cycle
GO:0031441 / negative regulation of mRNA 3'−end processing
GO:0048207 / vesicle targeting, rough ER to cis−Golgi
GO:0048208 / COPII vesicle coating
GO:0036257 / multivesicular body organization
GO:0033119 / negative regulation of RNA splicing
GO:0042542 / response to hydrogen peroxide

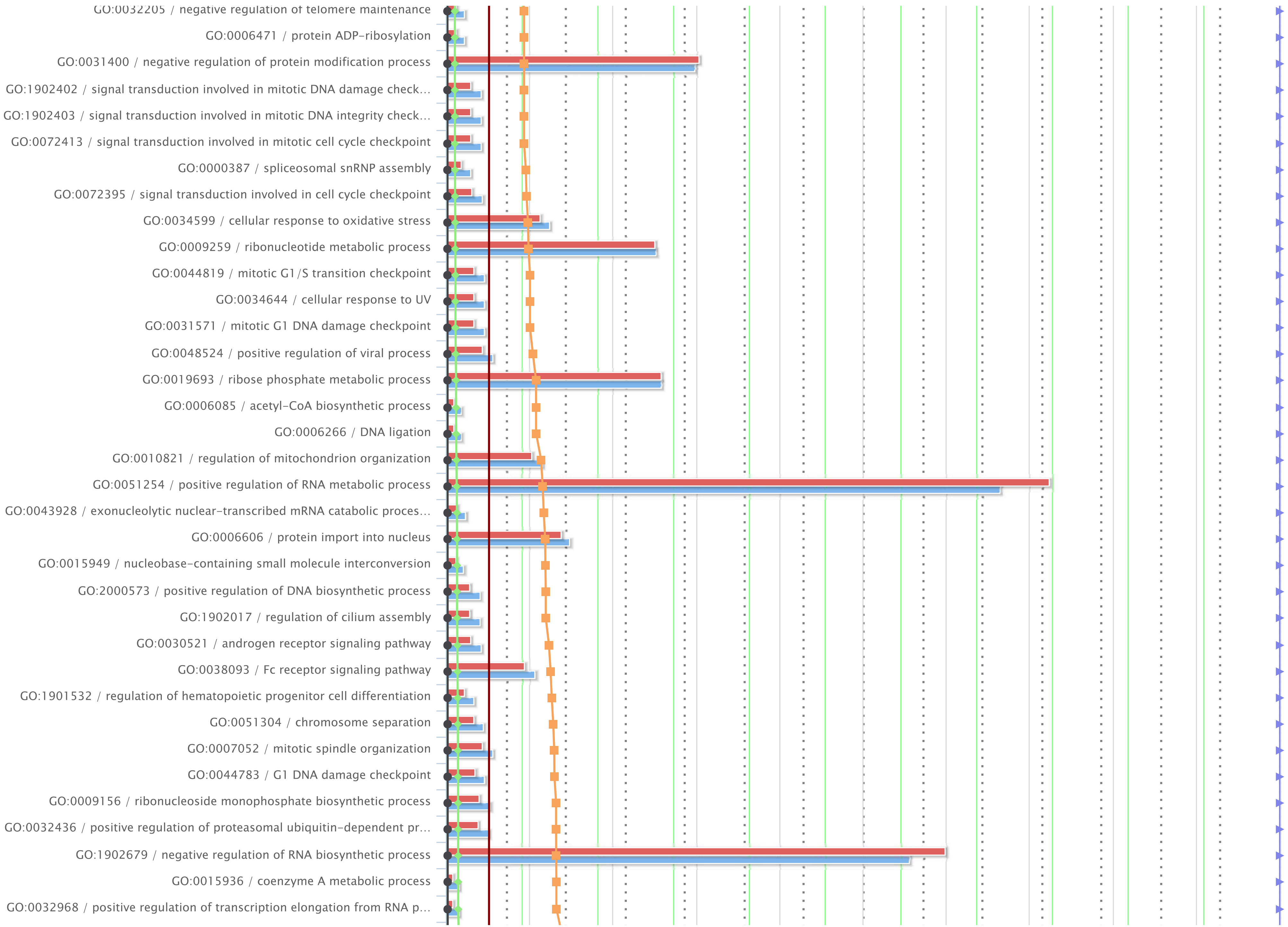
GO:0032205 / negative regulation of telomere maintenance
GO:0006471 / protein ADP–ribosylation
GO:0031400 / negative regulation of protein modification process
GO:1902402 / signal transduction involved in mitotic DNA damage check...
GO:1902403 / signal transduction involved in mitotic DNA integrity check...
GO:0072413 / signal transduction involved in mitotic cell cycle checkpoint
GO:0000387 / spliceosomal snRNP assembly
GO:0072395 / signal transduction involved in cell cycle checkpoint
GO:0034599 / cellular response to oxidative stress
GO:0009259 / ribonucleotide metabolic process
GO:0044819 / mitotic G1/S transition checkpoint
GO:0034644 / cellular response to UV
GO:0031571 / mitotic G1 DNA damage checkpoint
GO:0048524 / positive regulation of viral process
GO:0019693 / ribose phosphate metabolic process
GO:0006085 / acetyl–CoA biosynthetic process
GO:0006266 / DNA ligation
GO:0010821 / regulation of mitochondrion organization
GO:0051254 / positive regulation of RNA metabolic process
GO:0043928 / exonucleolytic nuclear–transcribed mRNA catabolic proces...
GO:0006606 / protein import into nucleus
GO:0015949 / nucleobase–containing small molecule interconversion
GO:2000573 / positive regulation of DNA biosynthetic process
GO:1902017 / regulation of cilium assembly
GO:0030521 / androgen receptor signaling pathway
GO:0038093 / Fc receptor signaling pathway
GO:1901532 / regulation of hematopoietic progenitor cell differentiation
GO:0051304 / chromosome separation
GO:0007052 / mitotic spindle organization
GO:0044783 / G1 DNA damage checkpoint
GO:0009156 / ribonucleoside monophosphate biosynthetic process
GO:0032436 / positive regulation of proteasomal ubiquitin–dependent pr...
GO:1902679 / negative regulation of RNA biosynthetic process
GO:0015936 / coenzyme A metabolic process
GO:0032968 / positive regulation of transcription elongation from RNA p...

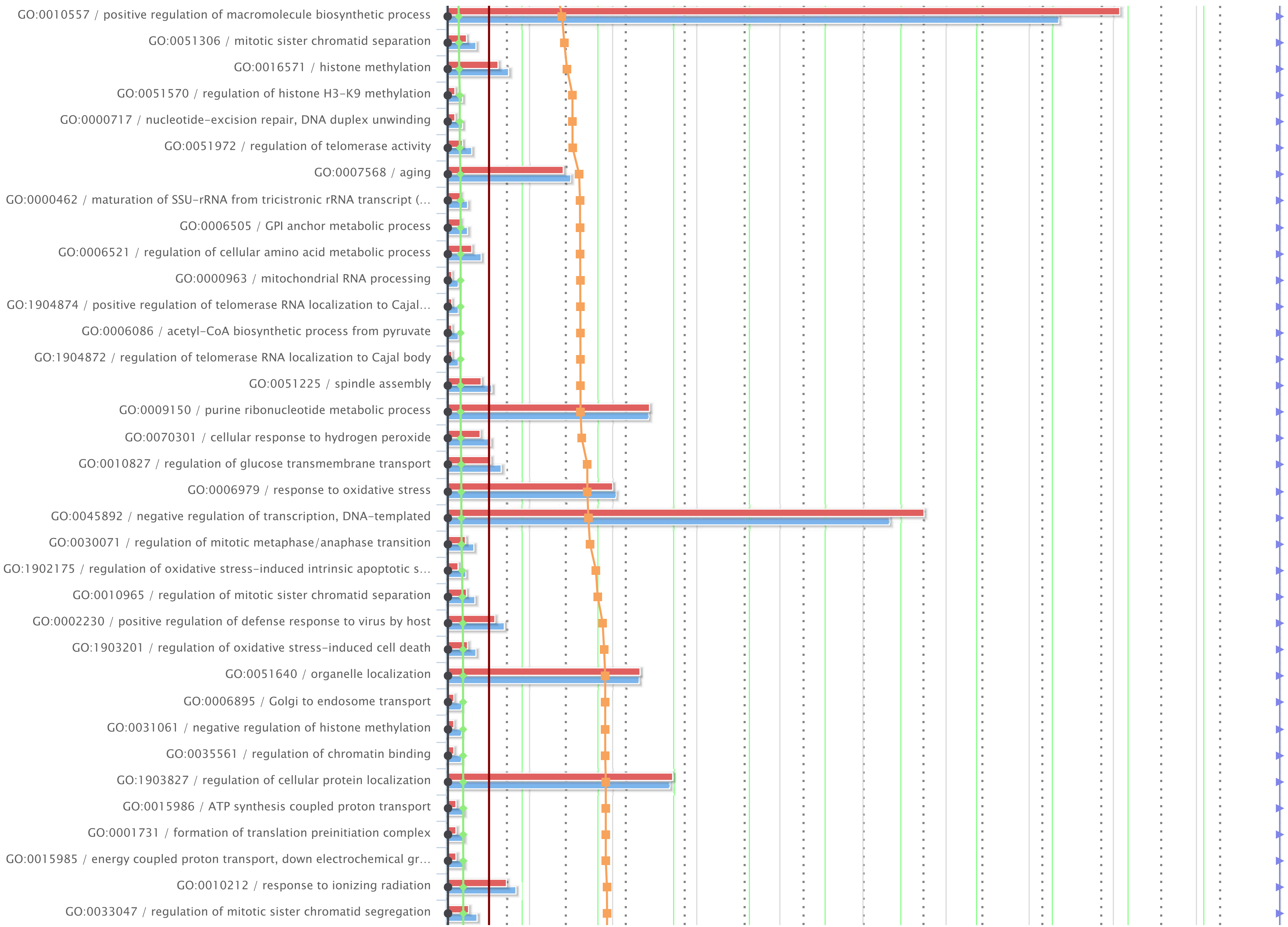
GO:0010557 / positive regulation of macromolecule biosynthetic process
GO:0051306 / mitotic sister chromatid separation
GO:0016571 / histone methylation
GO:0051570 / regulation of histone H3-K9 methylation
GO:0000717 / nucleotide-excision repair, DNA duplex unwinding
GO:0051972 / regulation of telomerase activity
GO:0007568 / aging
GO:0000462 / maturation of SSU-rRNA from tricistronic rRNA transcript (...
GO:0006505 / GPI anchor metabolic process
GO:0006521 / regulation of cellular amino acid metabolic process
GO:0000963 / mitochondrial RNA processing
GO:1904874 / positive regulation of telomerase RNA localization to Cajal...
GO:0006086 / acetyl-CoA biosynthetic process from pyruvate
GO:1904872 / regulation of telomerase RNA localization to Cajal body
GO:0051225 / spindle assembly
GO:0009150 / purine ribonucleotide metabolic process
GO:0070301 / cellular response to hydrogen peroxide
GO:0010827 / regulation of glucose transmembrane transport
GO:0006979 / response to oxidative stress
GO:0045892 / negative regulation of transcription, DNA-templated
GO:0030071 / regulation of mitotic metaphase/anaphase transition
GO:1902175 / regulation of oxidative stress-induced intrinsic apoptotic s...
GO:0010965 / regulation of mitotic sister chromatid separation
GO:0002230 / positive regulation of defense response to virus by host
GO:1903201 / regulation of oxidative stress-induced cell death
GO:0051640 / organelle localization
GO:0006895 / Golgi to endosome transport
GO:0031061 / negative regulation of histone methylation
GO:0035561 / regulation of chromatin binding
GO:1903827 / regulation of cellular protein localization
GO:0015986 / ATP synthesis coupled proton transport
GO:0001731 / formation of translation preinitiation complex
GO:0015985 / energy coupled proton transport, down electrochemical gr...
GO:0010212 / response to ionizing radiation
GO:0033047 / regulation of mitotic sister chromatid segregation

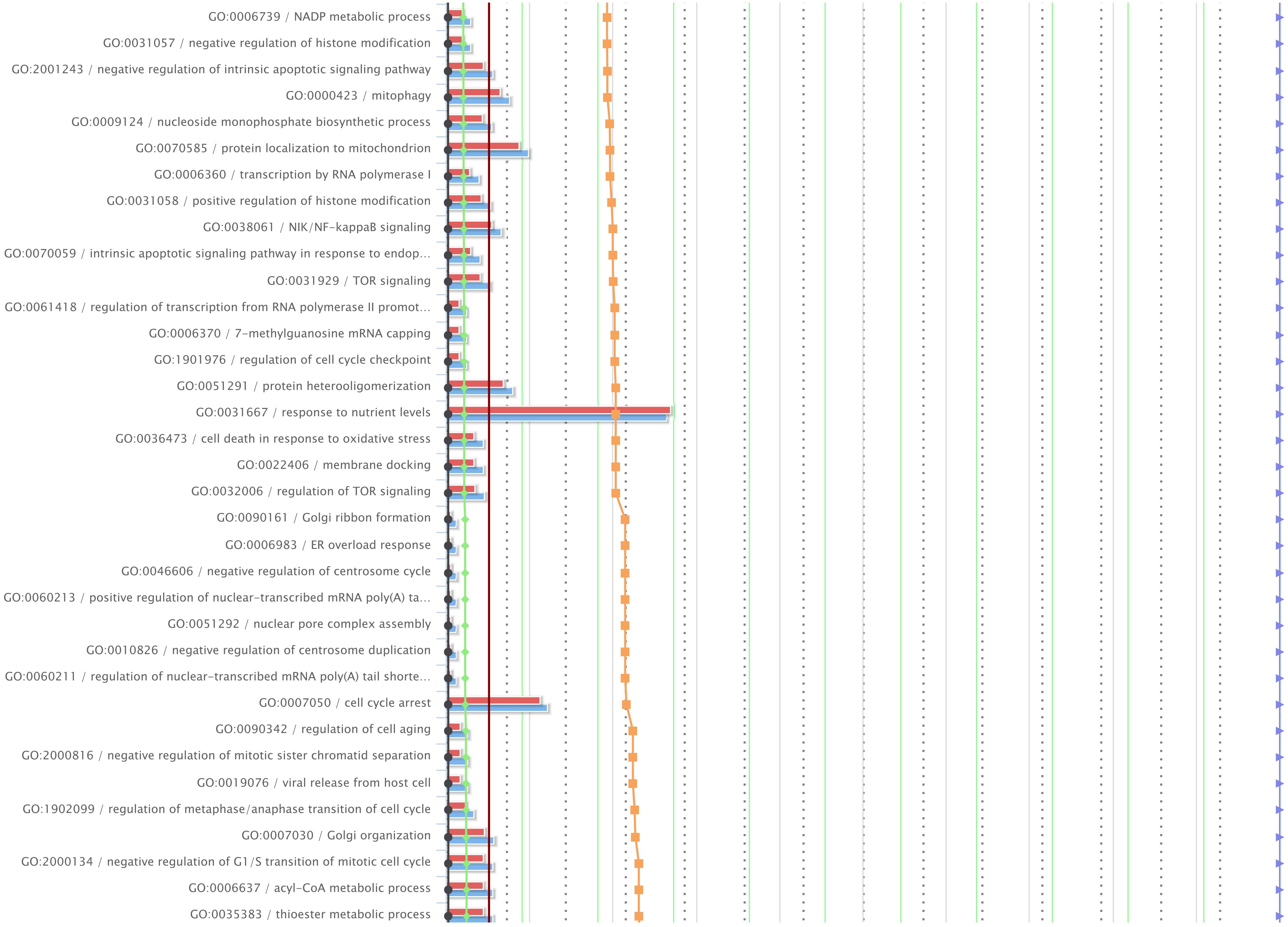
GO:0006739 / NADP metabolic process
GO:0031057 / negative regulation of histone modification
GO:2001243 / negative regulation of intrinsic apoptotic signaling pathway
GO:0000423 / mitophagy
GO:0009124 / nucleoside monophosphate biosynthetic process
GO:0070585 / protein localization to mitochondrion
GO:0006360 / transcription by RNA polymerase I
GO:0031058 / positive regulation of histone modification
GO:0038061 / NIK/NF-kappaB signaling
GO:0070059 / intrinsic apoptotic signaling pathway in response to endop...
GO:0031929 / TOR signaling
GO:0061418 / regulation of transcription from RNA polymerase II promot...
GO:0006370 / 7-methylguanosine mRNA capping
GO:1901976 / regulation of cell cycle checkpoint
GO:0051291 / protein heterooligomerization
GO:0031667 / response to nutrient levels
GO:0036473 / cell death in response to oxidative stress
GO:0022406 / membrane docking
GO:0032006 / regulation of TOR signaling
GO:0090161 / Golgi ribbon formation
GO:0006983 / ER overload response
GO:0046606 / negative regulation of centrosome cycle
GO:0060213 / positive regulation of nuclear-transcribed mRNA poly(A) ta...
GO:0051292 / nuclear pore complex assembly
GO:0010826 / negative regulation of centrosome duplication
GO:0060211 / regulation of nuclear-transcribed mRNA poly(A) tail shorte...
GO:0007050 / cell cycle arrest
GO:0090342 / regulation of cell aging
GO:2000816 / negative regulation of mitotic sister chromatid separation
GO:0019076 / viral release from host cell
GO:1902099 / regulation of metaphase/anaphase transition of cell cycle
GO:0007030 / Golgi organization
GO:2000134 / negative regulation of G1/S transition of mitotic cell cycle
GO:0006637 / acyl-CoA metabolic process
GO:0035383 / thioester metabolic process

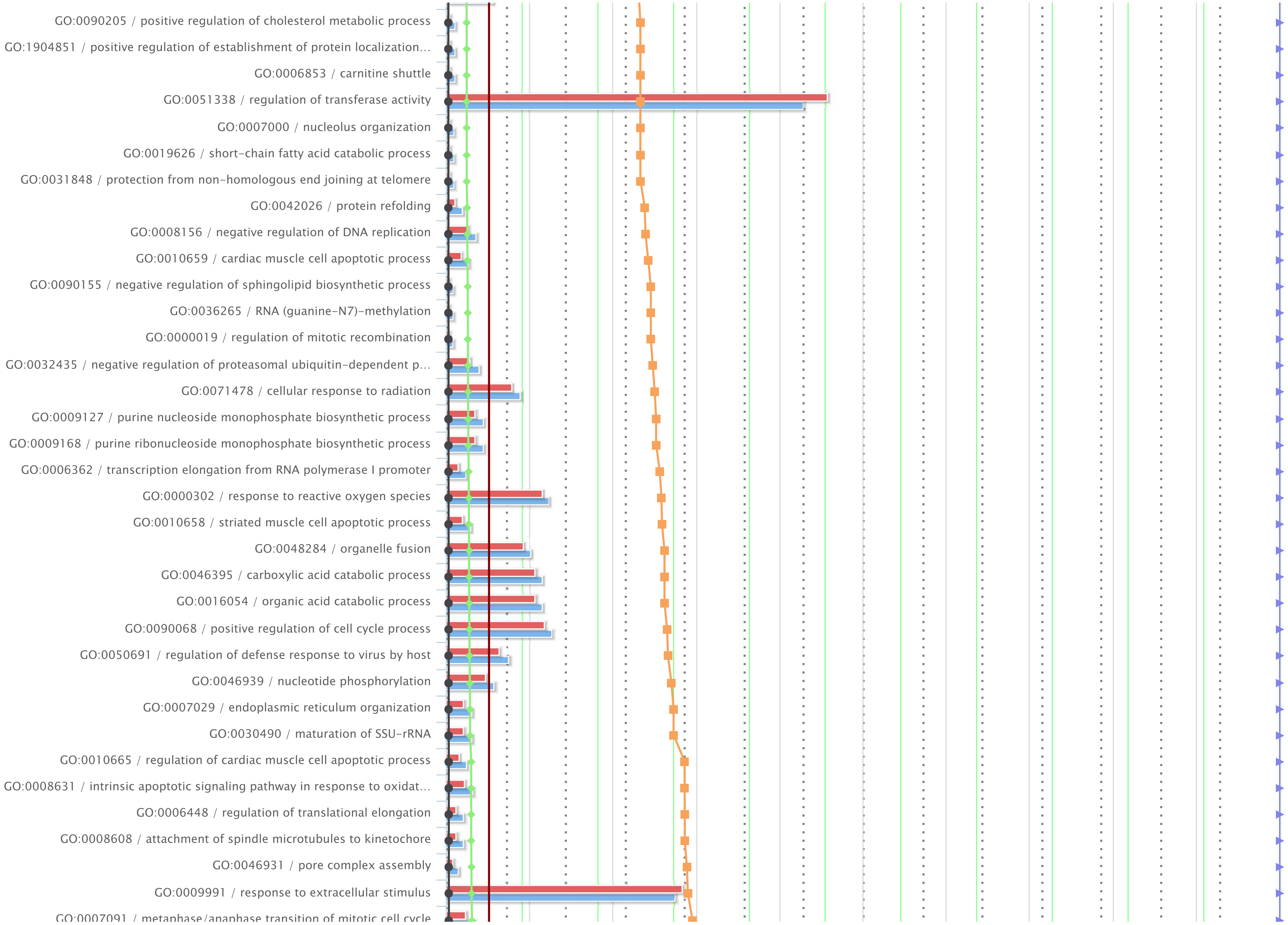

GO:0090205 / positive regulation of cholesterol metabolic process
GO:1904851 / positive regulation of establishment of protein localization...
GO:0006853 / carnitine shuttle
GO:0051338 / regulation of transferase activity
GO:0007000 / nucleolus organization
GO:0019626 / short-chain fatty acid catabolic process
GO:0031848 / protection from non-homologous end joining at telomere
GO:0042026 / protein refolding
GO:0008156 / negative regulation of DNA replication
GO:0010659 / cardiac muscle cell apoptotic process
GO:0090155 / negative regulation of sphingolipid biosynthetic process
GO:0036265 / RNA (guanine-N7)-methylation
GO:0000019 / regulation of mitotic recombination
GO:0032435 / negative regulation of proteasomal ubiquitin-dependent p...
GO:0071478 / cellular response to radiation
GO:0009127 / purine nucleoside monophosphate biosynthetic process
GO:0009168 / purine ribonucleoside monophosphate biosynthetic process
GO:0006362 / transcription elongation from RNA polymerase I promoter
GO:0000302 / response to reactive oxygen species
GO:0010658 / striated muscle cell apoptotic process
GO:0048284 / organelle fusion
GO:0046395 / carboxylic acid catabolic process
GO:0016054 / organic acid catabolic process
GO:0090068 / positive regulation of cell cycle process
GO:0050691 / regulation of defense response to virus by host
GO:0046939 / nucleotide phosphorylation
GO:0007029 / endoplasmic reticulum organization
GO:0030490 / maturation of SSU-rRNA
GO:0010665 / regulation of cardiac muscle cell apoptotic process
GO:0008631 / intrinsic apoptotic signaling pathway in response to oxidat...
GO:0006448 / regulation of translational elongation
GO:0008608 / attachment of spindle microtubules to kinetochore
GO:0046931 / pore complex assembly
GO:0009991 / response to extracellular stimulus
GO:0007091 / metaphase/anaphase transition of mitotic cell cycle

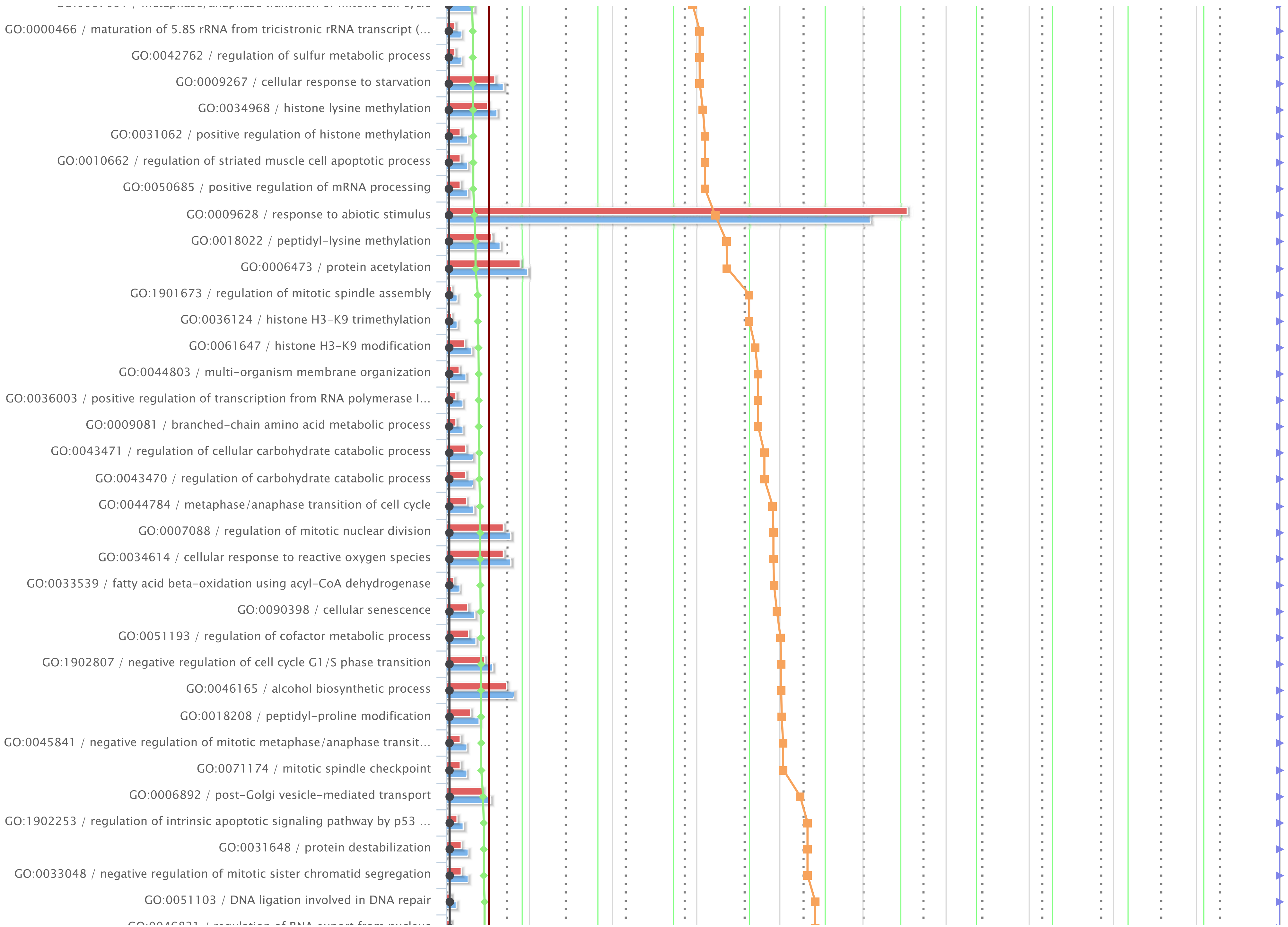

GO:0000466 / maturation of 5.8S rRNA from tricistronic rRNA transcript (...
GO:0042762 / regulation of sulfur metabolic process
GO:0009267 / cellular response to starvation
GO:0034968 / histone lysine methylation
GO:0031062 / positive regulation of histone methylation
GO:0010662 / regulation of striated muscle cell apoptotic process
GO:0050685 / positive regulation of mRNA processing
GO:0009628 / response to abiotic stimulus
GO:0018022 / peptidyl-lysine methylation
GO:0006473 / protein acetylation
GO:1901673 / regulation of mitotic spindle assembly
GO:0036124 / histone H3-K9 trimethylation
GO:0061647 / histone H3-K9 modification
GO:0044803 / multi-organism membrane organization
GO:0036003 / positive regulation of transcription from RNA polymerase I...
GO:0009081 / branched-chain amino acid metabolic process
GO:0043471 / regulation of cellular carbohydrate catabolic process
GO:0043470 / regulation of carbohydrate catabolic process
GO:0044784 / metaphase/anaphase transition of cell cycle
GO:0007088 / regulation of mitotic nuclear division
GO:0034614 / cellular response to reactive oxygen species
GO:0033539 / fatty acid beta-oxidation using acyl-CoA dehydrogenase
GO:0090398 / cellular senescence
GO:0051193 / regulation of cofactor metabolic process
GO:1902807 / negative regulation of cell cycle G1/S phase transition
GO:0046165 / alcohol biosynthetic process
GO:0018208 / peptidyl-proline modification
GO:0045841 / negative regulation of mitotic metaphase/anaphase transit...
GO:0071174 / mitotic spindle checkpoint
GO:0006892 / post-Golgi vesicle-mediated transport
GO:1902253 / regulation of intrinsic apoptotic signaling pathway by p53 ...
GO:0031648 / protein destabilization
GO:0033048 / negative regulation of mitotic sister chromatid segregation
GO:0051103 / DNA ligation involved in DNA repair

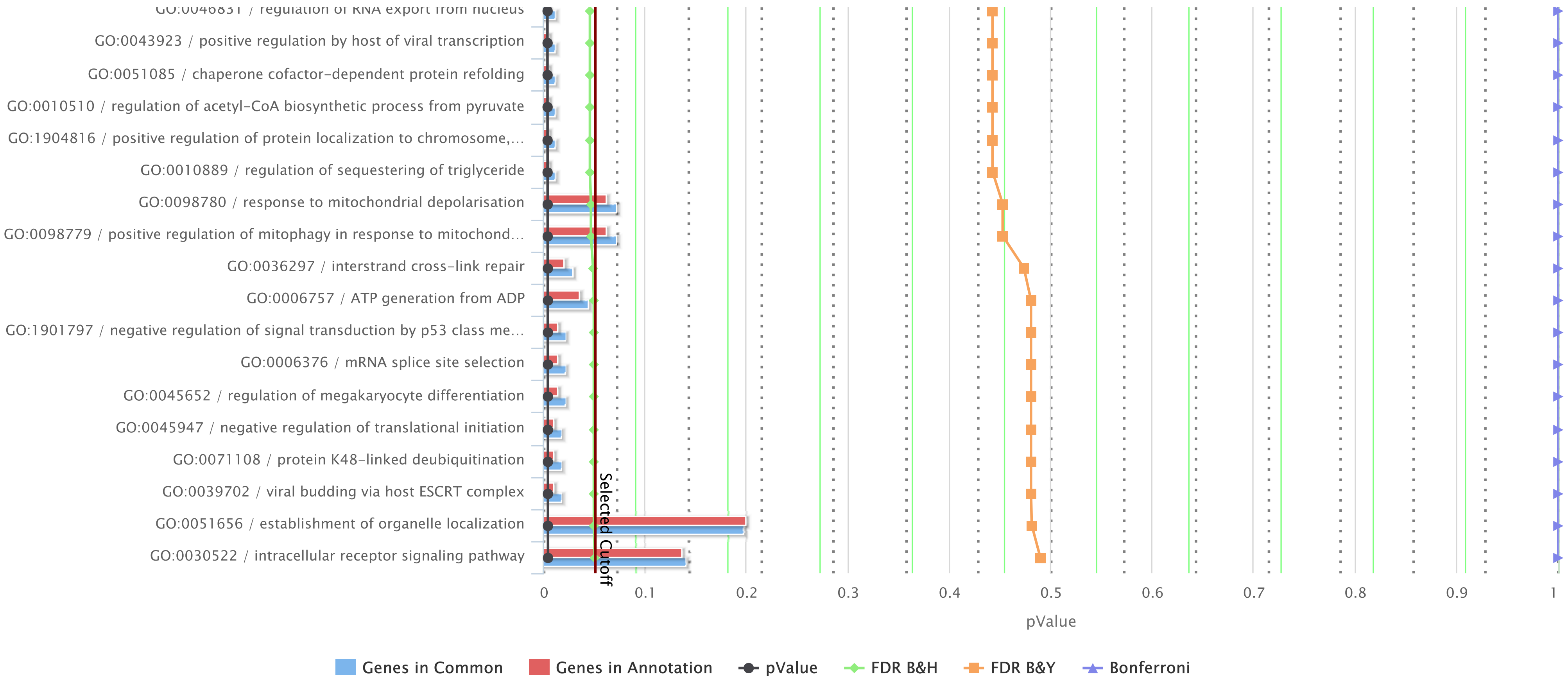
GO:0046831 / regulation of RNA export from nucleus
GO:0043923 / positive regulation by host of viral transcription
GO:0051085 / chaperone cofactor-dependent protein refolding
GO:0010510 / regulation of acetyl-CoA biosynthetic process from pyruvate
GO:1904816 / positive regulation of protein localization to chromosome,...
GO:0010889 / regulation of sequestering of triglyceride
GO:0098780 / response to mitochondrial depolarisation
GO:0098779 / positive regulation of mitophagy in response to mitochond...
GO:0036297 / interstrand cross-link repair
GO:0006757 / ATP generation from ADP
GO:1901797 / negative regulation of signal transduction by p53 class me...
GO:0006376 / mRNA splice site selection
GO:0045652 / regulation of megakaryocyte differentiation
GO:0045947 / negative regulation of translational initiation
GO:0071108 / protein K48-linked deubiquitination
GO:0039702 / viral budding via host ESCRT complex
GO:0051656 / establishment of organelle localization
GO:0030522 / intracellular receptor signaling pathway
Selected Cutoff
0
0.1
0.2
0.3
0.4
0.5
0.6
0.7
0.8
0.9
1
pValue
Genes in Common
Genes in Annotation
pValue
FDR B&H
FDR B&Y
Bonferroni

Supplementary Figure 5F
Hypoacetylated genes

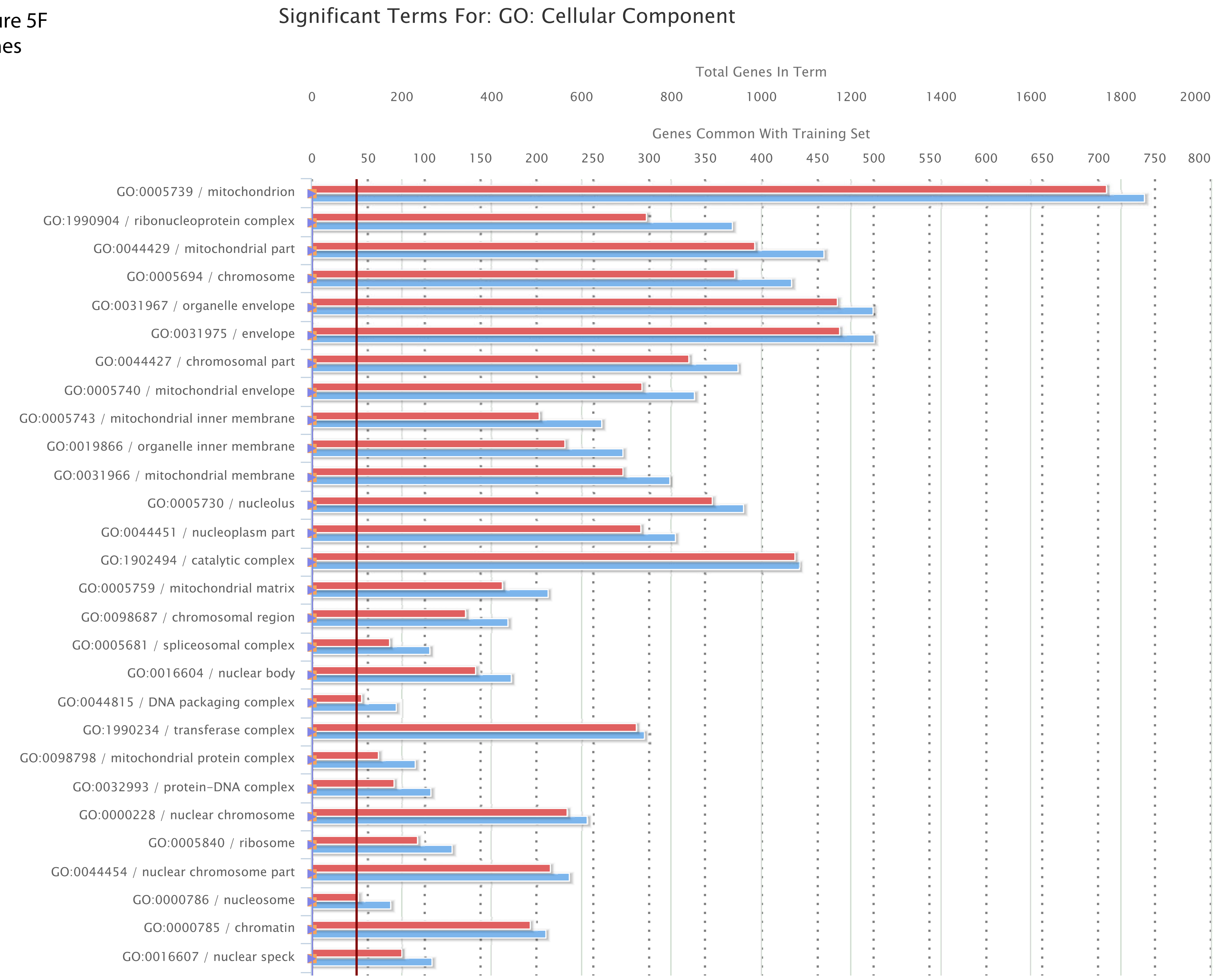

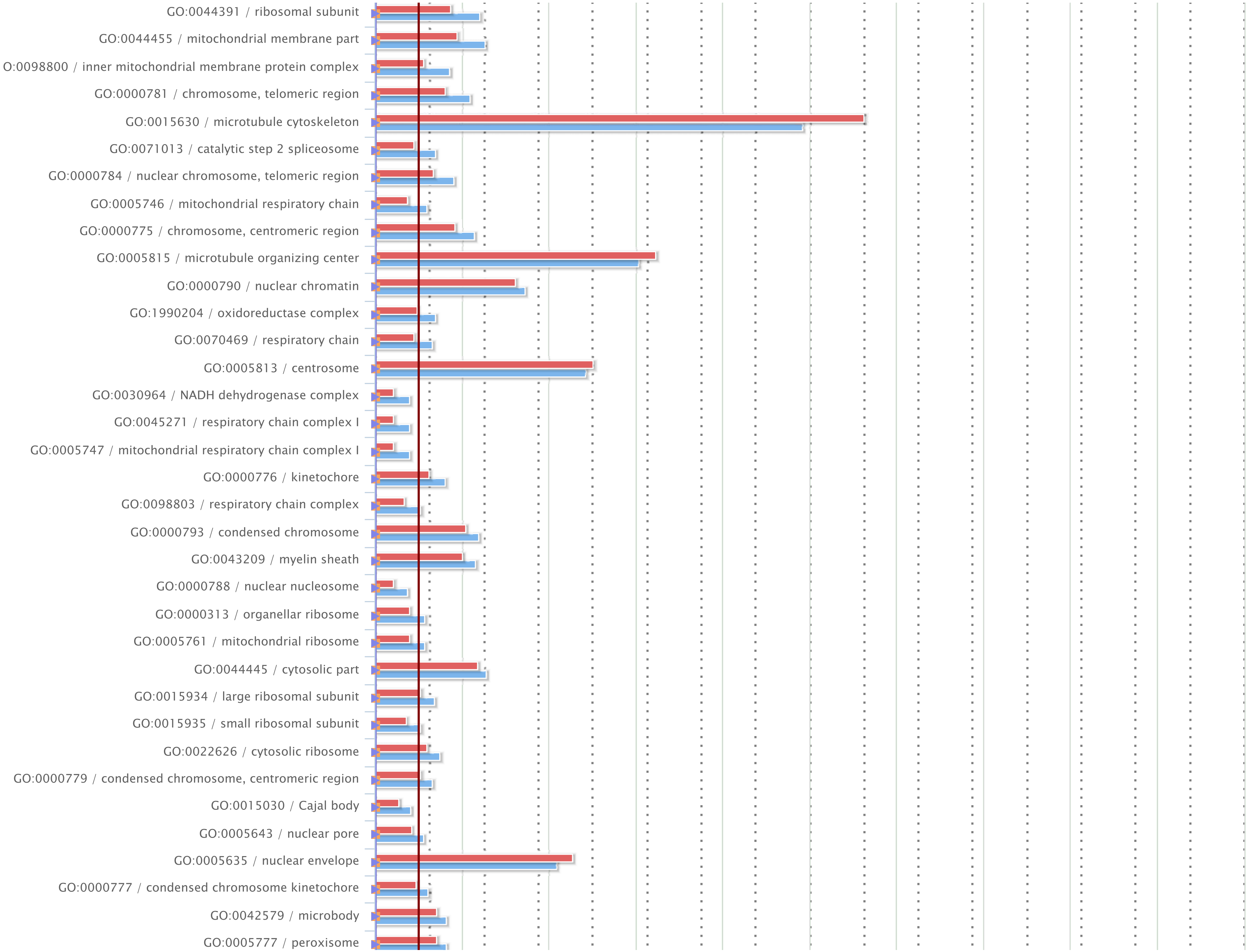
GO:0044391 / ribosomal subunit
GO:0044455 / mitochondrial membrane part
O:0098800 / inner mitochondrial membrane protein complex
GO:0000781 / chromosome, telomeric region
GO:0015630 / microtubule cytoskeleton
GO:0071013 / catalytic step 2 spliceosome
GO:0000784 / nuclear chromosome, telomeric region
GO:0005746 / mitochondrial respiratory chain
GO:0000775 / chromosome, centromeric region
GO:0005815 / microtubule organizing center
GO:0000790 / nuclear chromatin
GO:1990204 / oxidoreductase complex
GO:0070469 / respiratory chain
GO:0005813 / centrosome
GO:0030964 / NADH dehydrogenase complex
GO:0045271 / respiratory chain complex I
GO:0005747 / mitochondrial respiratory chain complex I
GO:0000776 / kinetochore
GO:0098803 / respiratory chain complex
GO:0000793 / condensed chromosome
GO:0043209 / myelin sheath
GO:0000788 / nuclear nucleosome
GO:0000313 / organellar ribosome
GO:0005761 / mitochondrial ribosome
GO:0044445 / cytosolic part
GO:0015934 / large ribosomal subunit
GO:0015935 / small ribosomal subunit
GO:0022626 / cytosolic ribosome
GO:0000779 / condensed chromosome, centromeric region
GO:0015030 / Cajal body
GO:0005643 / nuclear pore
GO:0005635 / nuclear envelope
GO:0000777 / condensed chromosome kinetochore
GO:0042579 / microbody
GO:0005777 / peroxisome

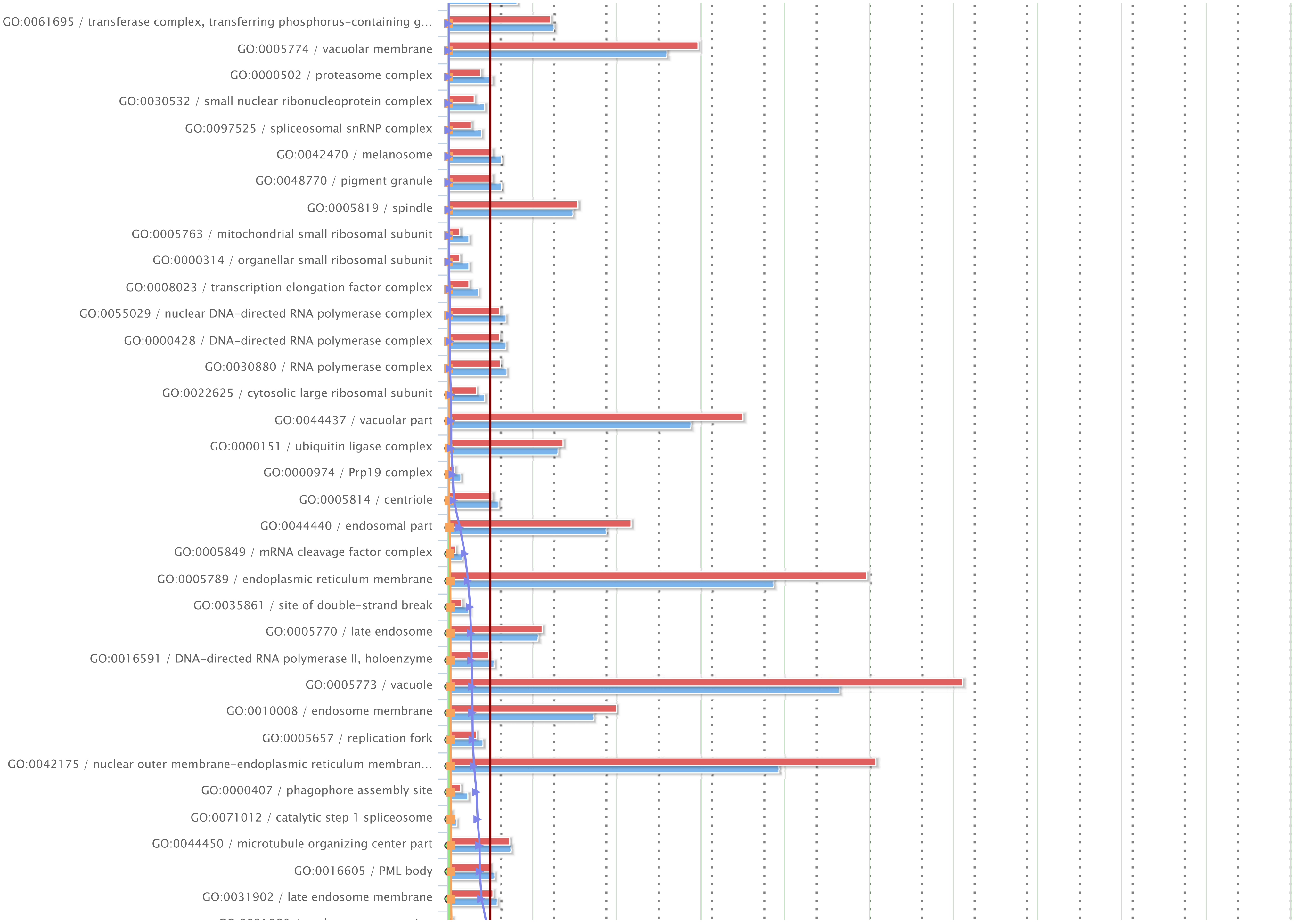
GO:0061695 / transferase complex, transferring phosphorus-containing g...
GO:0005774 / vacuolar membrane
GO:0000502 / proteasome complex
GO:0030532 / small nuclear ribonucleoprotein complex
GO:0097525 / spliceosomal snRNP complex
GO:0042470 / melanosome
GO:0048770 / pigment granule
GO:0005819 / spindle
GO:0005763 / mitochondrial small ribosomal subunit
GO:0000314 / organellar small ribosomal subunit
GO:0008023 / transcription elongation factor complex
GO:0055029 / nuclear DNA-directed RNA polymerase complex
GO:0000428 / DNA-directed RNA polymerase complex
GO:0030880 / RNA polymerase complex
GO:0022625 / cytosolic large ribosomal subunit
GO:0044437 / vacuolar part
GO:0000151 / ubiquitin ligase complex
GO:0000974 / Prp19 complex
GO:0005814 / centriole
GO:0044440 / endosomal part
GO:0005849 / mRNA cleavage factor complex
GO:0005789 / endoplasmic reticulum membrane
GO:0035861 / site of double-strand break
GO:0005770 / late endosome
GO:0016591 / DNA-directed RNA polymerase II, holoenzyme
GO:0005773 / vacuole
GO:0010008 / endosome membrane
GO:0005657 / replication fork
GO:0042175 / nuclear outer membrane-endoplasmic reticulum membran...
GO:0000407 / phagophore assembly site
GO:0071012 / catalytic step 1 spliceosome
GO:0044450 / microtubule organizing center part
GO:0016605 / PML body
GO:0031902 / late endosome membrane

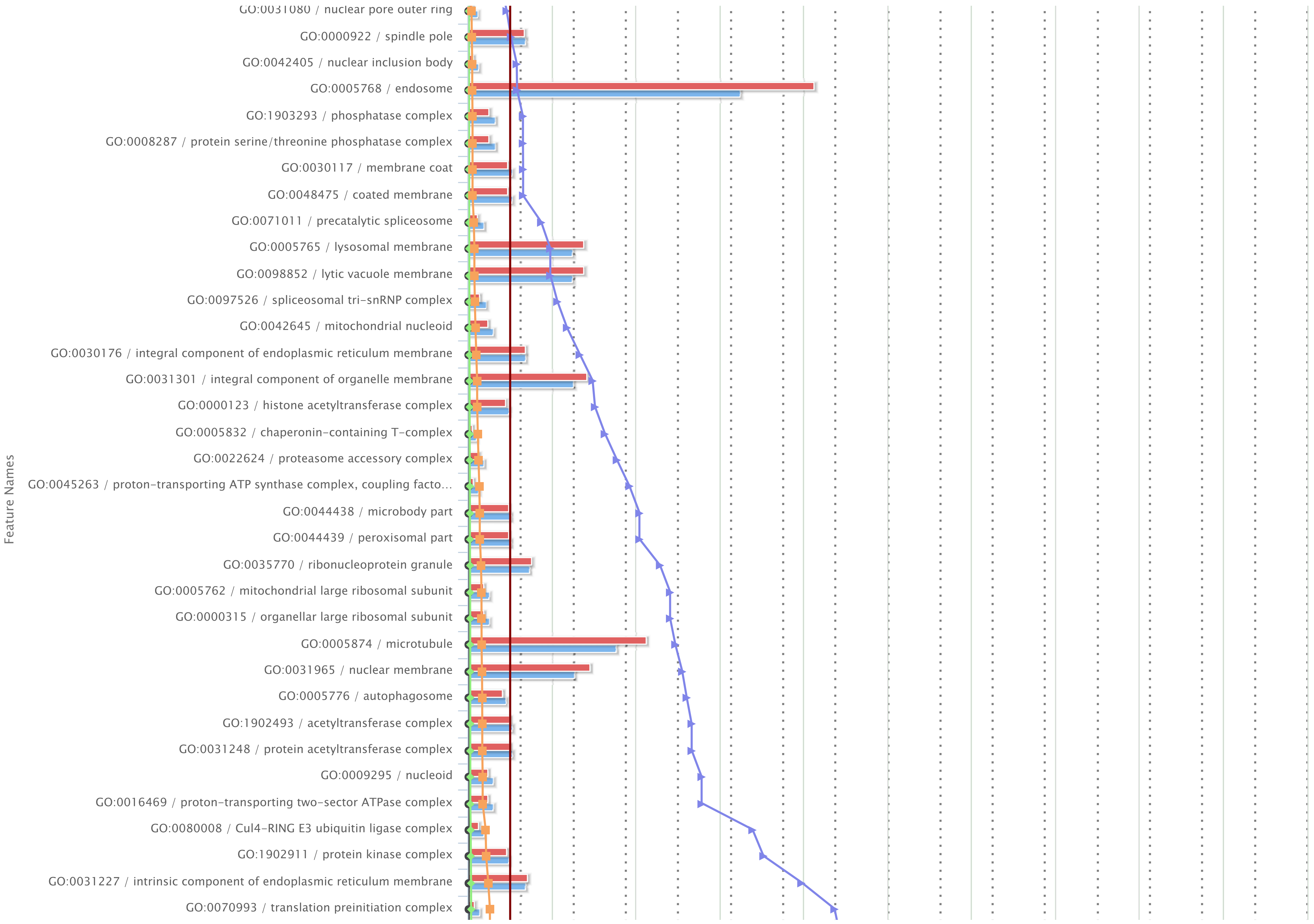

Feature Names
GO:0031080 / nuclear pore outer ring
GO:0000922 / spindle pole
GO:0042405 / nuclear inclusion body
GO:0005768 / endosome
GO:1903293 / phosphatase complex
GO:0008287 / protein serine/threonine phosphatase complex
GO:0030117 / membrane coat
GO:0048475 / coated membrane
GO:0071011 / precatalytic spliceosome
GO:0005765 / lysosomal membrane
GO:0098852 / lytic vacuole membrane
GO:0097526 / spliceosomal tri−snRNP complex
GO:0042645 / mitochondrial nucleoid
GO:0030176 / integral component of endoplasmic reticulum membrane
GO:0031301 / integral component of organelle membrane
GO:0000123 / histone acetyltransferase complex
GO:0005832 / chaperonin−containing T−complex
GO:0022624 / proteasome accessory complex
GO:0045263 / proton−transporting ATP synthase complex, coupling facto...
GO:0044438 / microbody part
GO:0044439 / peroxisomal part
GO:0035770 / ribonucleoprotein granule
GO:0005762 / mitochondrial large ribosomal subunit
GO:0000315 / organellar large ribosomal subunit
GO:0005874 / microtubule
GO:0031965 / nuclear membrane
GO:0005776 / autophagosome
GO:1902493 / acetyltransferase complex
GO:0031248 / protein acetyltransferase complex
GO:0009295 / nucleoid
GO:0016469 / proton−transporting two−sector ATPase complex
GO:0080008 / Cul4−RING E3 ubiquitin ligase complex
GO:1902911 / protein kinase complex
GO:0031227 / intrinsic component of endoplasmic reticulum membrane
GO:0070993 / translation preinitiation complex

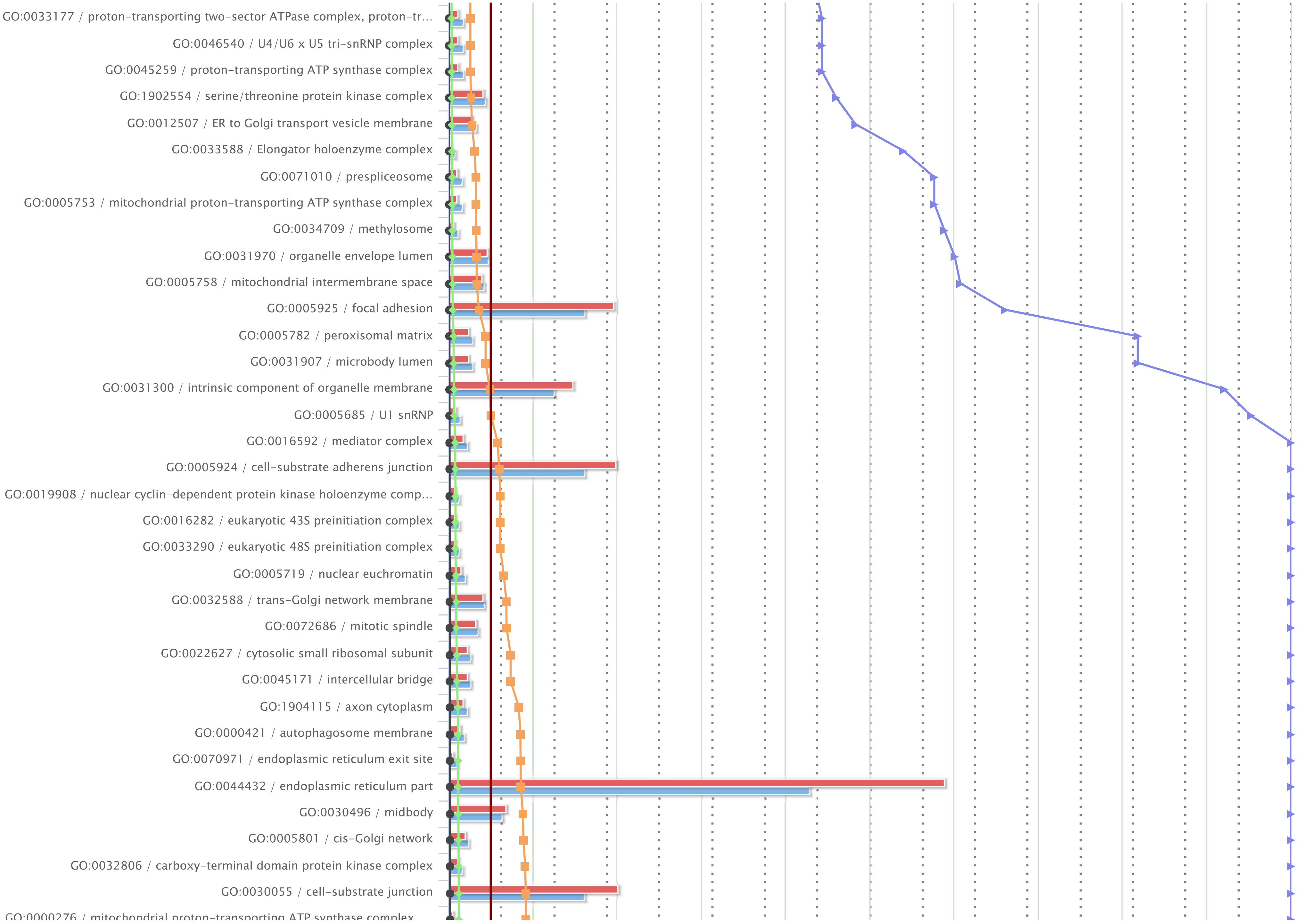
GO:0033177 / proton-transporting two-sector ATPase complex, proton-tr...
GO:0046540 / U4/U6 x U5 tri-snRNP complex
GO:0045259 / proton-transporting ATP synthase complex
GO:1902554 / serine/threonine protein kinase complex
GO:0012507 / ER to Golgi transport vesicle membrane
GO:0033588 / Elongator holoenzyme complex
GO:0071010 / prespliceosome
GO:0005753 / mitochondrial proton-transporting ATP synthase complex
GO:0034709 / methylosome
GO:0031970 / organelle envelope lumen
GO:0005758 / mitochondrial intermembrane space
GO:0005925 / focal adhesion
GO:0005782 / peroxisomal matrix
GO:0031907 / microbody lumen
GO:0031300 / intrinsic component of organelle membrane
GO:0005685 / U1 snRNP
GO:0016592 / mediator complex
GO:0005924 / cell-substrate adherens junction
GO:0019908 / nuclear cyclin-dependent protein kinase holoenzyme comp...
GO:0016282 / eukaryotic 43S preinitiation complex
GO:0033290 / eukaryotic 48S preinitiation complex
GO:0005719 / nuclear euchromatin
GO:0032588 / trans-Golgi network membrane
GO:0072686 / mitotic spindle
GO:0022627 / cytosolic small ribosomal subunit
GO:0045171 / intercellular bridge
GO:1904115 / axon cytoplasm
GO:0000421 / autophagosome membrane
GO:0070971 / endoplasmic reticulum exit site
GO:0044432 / endoplasmic reticulum part
GO:0030496 / midbody
GO:0005801 / cis-Golgi network
GO:0032806 / carboxy-terminal domain protein kinase complex
GO:0030055 / cell-substrate junction
GO:0000276 / mitochondrial proton-transporting ATP synthase complex

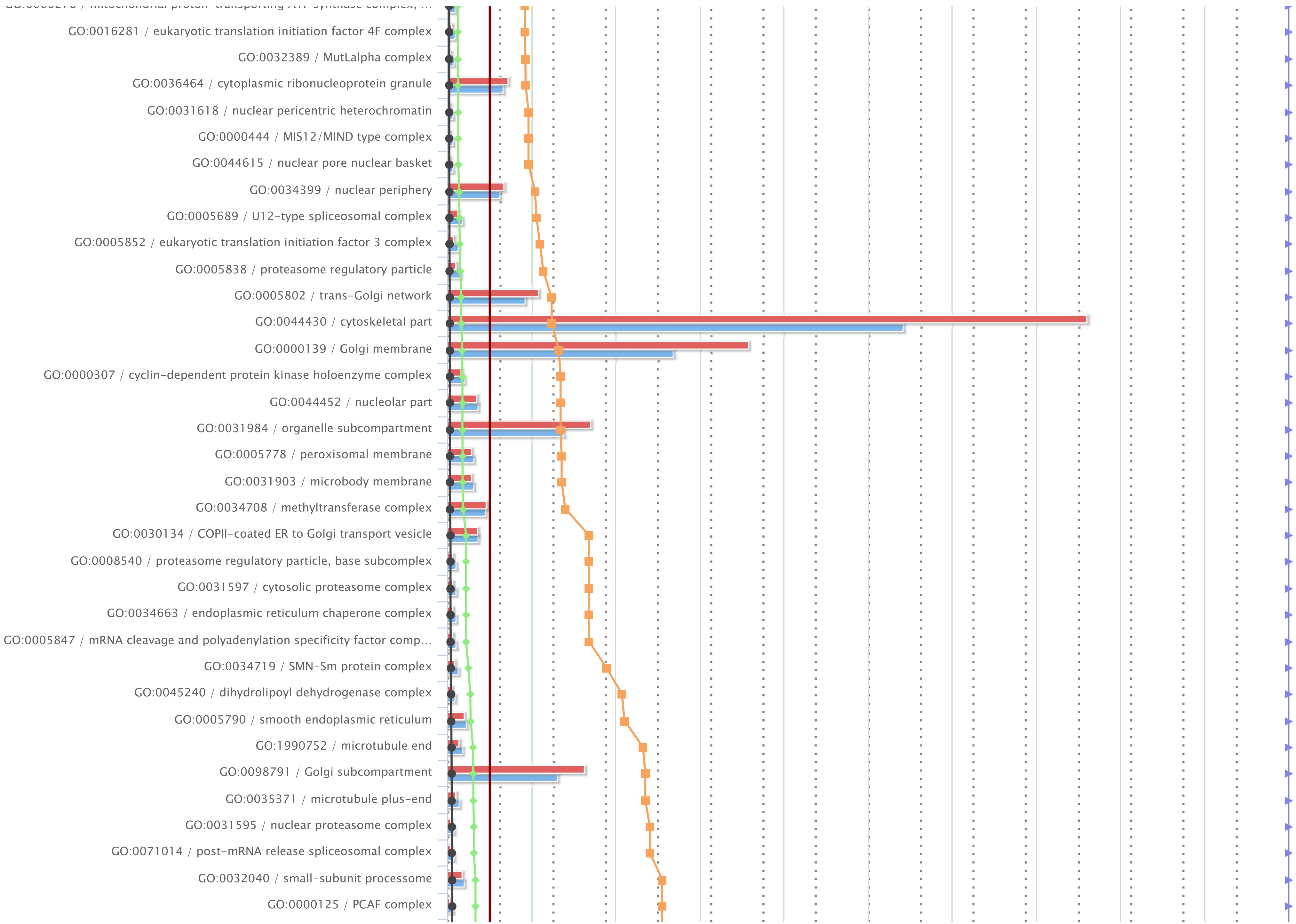

GO:0016281 / eukaryotic translation initiation factor 4F complex
GO:0032389 / MutLalpha complex
GO:0036464 / cytoplasmic ribonucleoprotein granule
GO:0031618 / nuclear pericentric heterochromatin
GO:0000444 / MIS12/MIND type complex
GO:0044615 / nuclear pore nuclear basket
GO:0034399 / nuclear periphery
GO:0005689 / U12-type spliceosomal complex
GO:0005852 / eukaryotic translation initiation factor 3 complex
GO:0005838 / proteasome regulatory particle
GO:0005802 / trans-Golgi network
GO:0044430 / cytoskeletal part
GO:0000139 / Golgi membrane
GO:0000307 / cyclin-dependent protein kinase holoenzyme complex
GO:0044452 / nucleolar part
GO:0031984 / organelle subcompartment
GO:0005778 / peroxisomal membrane
GO:0031903 / microbody membrane
GO:0034708 / methyltransferase complex
GO:0030134 / COPII-coated ER to Golgi transport vesicle
GO:0008540 / proteasome regulatory particle, base subcomplex
GO:0031597 / cytosolic proteasome complex
GO:0034663 / endoplasmic reticulum chaperone complex
GO:0005847 / mRNA cleavage and polyadenylation specificity factor comp...
GO:0034719 / SMN-Sm protein complex
GO:0045240 / dihydrolipoyl dehydrogenase complex
GO:0005790 / smooth endoplasmic reticulum
GO:1990752 / microtubule end
GO:0098791 / Golgi subcompartment
GO:0035371 / microtubule plus-end
GO:0031595 / nuclear proteasome complex
GO:0071014 / post-mRNA release spliceosomal complex
GO:0032040 / small-subunit processome
GO:0000125 / PCAF complex

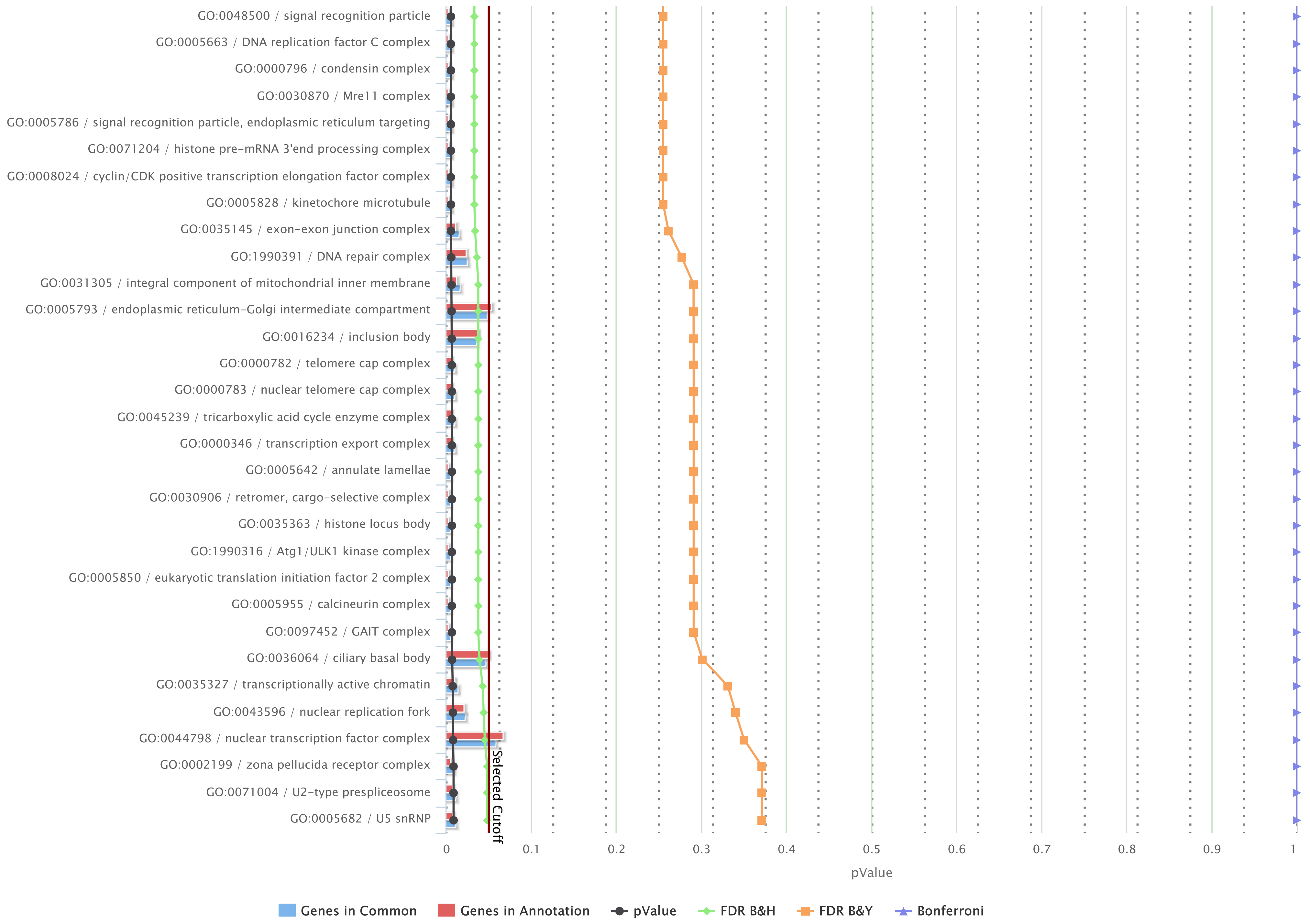
GO:0048500 / signal recognition particle
GO:0005663 / DNA replication factor C complex
GO:0000796 / condensin complex
GO:0030870 / Mre11 complex
GO:0005786 / signal recognition particle, endoplasmic reticulum targeting
GO:0071204 / histone pre-mRNA 3'end processing complex
GO:0008024 / cyclin/CDK positive transcription elongation factor complex
GO:0005828 / kinetochore microtubule
GO:0035145 / exon-exon junction complex
GO:1990391 / DNA repair complex
GO:0031305 / integral component of mitochondrial inner membrane
GO:0005793 / endoplasmic reticulum-Golgi intermediate compartment
GO:0016234 / inclusion body
GO:0000782 / telomere cap complex
GO:0000783 / nuclear telomere cap complex
GO:0045239 / tricarboxylic acid cycle enzyme complex
GO:0000346 / transcription export complex
GO:0005642 / annulate lamellae
GO:0030906 / retromer, cargo-selective complex
GO:0035363 / histone locus body
GO:1990316 / Atg1/ULK1 kinase complex
GO:0005850 / eukaryotic translation initiation factor 2 complex
GO:0005955 / calcineurin complex
GO:0097452 / GAIT complex
GO:0036064 / ciliary basal body
GO:0035327 / transcriptionally active chromatin
GO:0043596 / nuclear replication fork
GO:0044798 / nuclear transcription factor complex
GO:0002199 / zona pellucida receptor complex
GO:0071004 / U2-type prespliceosome
GO:0005682 / U5 snRNP
Selected Cutoff
0
0.1
0.2
0.3
0.4
0.5
0.6
0.7
0.8
0.9
1
pValue
Genes in Common
Genes in Annotation
pValue
FDR B&H
FDR B&Y
Bonferroni
