# Supplementary material for: H3K27ac acetylome signatures reveal the epigenomic reorganization in remodeled non-failing human hearts
Source: Clin Epigenetics. 2020 Jul 14;12:106. doi: 10.1186/s13148-020-00895-5 (PMC7362435; doi:10.1186/s13148-020-00895-5)
Supplement: Supplementary file 12 — Additional file 12. Supplementary Table 6. a An overview of general information of all included human cardiac samples. b An overview of detailed clinical parameters of the included AS patients. [file 13148_2020_895_MOESM12_ESM.docx]

**Supplementary Table 6A.** An overview of general information on all included human cardiac samples.

| Group | Sample ID | Septum Location | Source | Obtained Methods | Age | Gender | Left Ventricular Pattern |
| --- | --- | --- | --- | --- | --- | --- | --- |
| Controls | Control_1 | The septal region | St. Louis | Donor heart | 50 | Man | Normal |
|  | Control_2 | Halfway between the atrioventricular valves and the apex | UMC | Donor heart | NA | Woman | Normal |
|  | Control_3 | Left ventricule | UMC | Donor heart | NA | Man | Normal |
|  | Control_4 | Halfway between the atrioventricular valves and the apex | UMC | Post-mortem material | NA | Man | Normal |
|  | Control_5 | Halfway between the atrioventricular valves and the apex | UMC | Donor heart | NA | Woman | Normal |
| Patients | AS_1 | Near the atrioventricular valves | UCL | Fresh frozen biopsy | 65 | Man | LVH |
|  | AS_2 | Near the atrioventricular valves | UCL | Fresh frozen biopsy | 67 | Man | Concentric Remodelling |
|  | AS_3 | Near the atrioventricular valves | UCL | Fresh frozen biopsy | 77 | Woman | Concentric Remodelling |
|  | AS_4 | Near the atrioventricular valves | UCL | Fresh frozen biopsy | 57 | Man | LVH |
|  | AS_5 | Near the atrioventricular valves | UCL | Fresh frozen biopsy | 80 | Woman | Normal |
|  | AS_6 | Near the atrioventricular valves | UCL | Fresh frozen biopsy | 58 | Woman | Normal |
|  | AS_7 | Near the atrioventricular valves | UCL | Fresh frozen biopsy | 78 | Woman | Concentric Remodelling |
|  | AS_8 | Near the atrioventricular valves | UCL | Fresh frozen biopsy | 85 | Woman | Concentric remodelling |
|  | AS_9 | Near the atrioventricular valves | UCL | Fresh frozen biopsy | 65 | Woman | Concentric remodelling |
|  | AS_10 | Near the atrioventricular valves | UCL | Fresh frozen biopsy | 82 | Man | Concentric remodelling |
|  | AS_11 | Near the atrioventricular valves | UCL | Fresh frozen biopsy | 63 | Man | Concentric remodelling |
|  | AS_12 | Near the atrioventricular valves | UCL | Fresh frozen biopsy | 75 | Man | LVH |
|  | AS_13 | Near the atrioventricular valves | UCL | Fresh frozen biopsy | 81 | Man | concentric remodelling |
|  | AS_14 | Near the atrioventricular valves | UCL | Fresh frozen biopsy | 75 | Woman | LVH |
|  | AS_15 | Near the atrioventricular valves | UCL | Fresh frozen biopsy | 73 | Man | Concentric remodelling |
|  | AS_16 | Near the atrioventricular valves | UCL | Fresh frozen biopsy | 69 | Man | LVH |
|  | AS_17 | Near the atrioventricular valves | UCL | Fresh frozen biopsy | 76 | Man | Normal geometry |
|  | AS_18 | Near the atrioventricular valves | UCL | Fresh frozen biopsy | 64 | Man | Decompensation |
|  | AS_19 | Near the atrioventricular valves | UCL | Fresh frozen biopsy | 67 | Man | LVH |
|  | AS_20 | Near the atrioventricular valves | UCL | Fresh frozen biopsy | 65 | Man | LVH |

AS: aortic stenosis; UMC: University Medical Center Utrecht; UCL: University College London; LVH: left ventricular hypertrophy; NA: not available.

**Supplementary Table 6B.** An overview of detailed clinical parameters in included AS patients.

| Sample ID | LVEF (%) | LAAi (cm^2/m^2) | E/e' | EDVi | iMass index  (gram/m2) | M/V | EDV  (mls) | ESV  (mls) | SV | HR | BMI | NT-proBNP | Hypertension |
| --- | --- | --- | --- | --- | --- | --- | --- | --- | --- | --- | --- | --- | --- |
| AS_1 | 55.3 | 19.5 | 12.9 | 80.0 | 106.3 | 1.3 | 152 | 68 | 84 | 85 | 26.0 | 352 | + |
| AS_2 | 70.5 | 13.0 | 7.3 | 40.6 | 83.3 | 2.1 | 78 | 23 | 55 | 74 | 23.9 | 18 | - |
| AS_3 | 88.6 | 12.2 | 25.0 | 28.3 | 48.8 | 1.7 | 44 | 5 | 39 | 89 | 24.3 | 14 | - |
| AS_4 | 78.3 | 11.6 | 13.2 | 66.8 | 85.1 | 1.3 | 161 | 35 | 126 | 58 | 37.1 | 10 | + |
| AS_5 | 77.1 | 12.3 | NA | 56.1 | 52.6 | 0.9 | 96 | 22 | 74 | 67 | 28.3 | 26 | - |
| AS_6 | 82.0 | 15.9 | NA | 69.1 | 72.5 | 1.1 | 100 | 18 | 82 | 66 | 21.9 | 30 | - |
| AS_7 | 87.7 | 9.8 | 12.0 | 41.7 | 57.1 | 1.4 | 81 | 10 | 71 | 105 | 29.8 | 16 | + |
| AS_8 | 74.6 | 12.3 | 16.0 | 48.4 | 69.5 | 1.4 | 71 | 18 | 53 | 71 | 22.1 | 71 | + |
| AS_9 | 72.9 | 14.2 | 20.0 | 52.5 | 75.4 | 1.4 | 85 | 23 | 62 | 73 | 24.3 | NA | - |
| AS_10 | 85.5 | 16.4 | 7.3 | 55.5 | 65.6 | 1.2 | 110 | 16 | 94 | 72 | 24.7 | 18 | + |
| AS_11 | 78.0 | 10.3 | 10.1 | 49.5 | 79.7 | 1.6 | 82 | 18 | 64 | 81 | 23.2 | 12 | + |
| AS_12 | 57.4 | 19.6 | 12.9 | 63.4 | 123.9 | 2.0 | 136 | 58 | 78 | 72 | 30.2 | 422 | + |
| AS_13 | 86.7 | 11.6 | 9.0 | 41.9 | 69.7 | 1.7 | 83 | 11 | 72 | 62 | 29.8 | 19 | + |
| AS_14 | 77.4 | 11.4 | 12.3 | 56.8 | 95.3 | 1.7 | 115 | 26 | 89 | 84 | 33.5 | 97 | + |
| AS_15 | 78.4 | 10.3 | 12.7 | 65.9 | 75.3 | 1.1 | 134 | 29 | 105 | 77 | 27.8 | 29 | + |
| AS_16 | 62.0 | 10.9 | 12.5 | 43.6 | 106.6 | 2.4 | 92 | 35 | 57 | 75 | 34.4 | 91 | + |
| AS_17 | 76.8 | 13.6 | 12.7 | 57.6 | 91.7 | 1.6 | 125 | 29 | 96 | 61 | 35.8 | 159 | + |
| AS_18 | 34.9 | 14.3 | NA | 115.8 | 107.9 | 0.9 | 218 | 142 | 76 | 78 | 26.0 | 370 | NA |
| AS_19 | 51.9 | 12.3 | NA | 78.8 | 93.1 | 1.2 | 154 | 74 | 80 | 84 | 27.0 | 93 | NA |
| AS_20 | 64.3 | NA | NA | 65.0 | 107.2 | 1.7 | 143 | 51 | 92 | 68 | 34.9 | 47 | NA |

LVEF: left ventricular ejection fraction; LAAi: left atrial area index to body surface area; E/e': The maximum velocity of the E-wave of mitral valve inflow divided by the maximal velocity of E; EDVi: left ventricular end diastolic volume index; iMass index: left ventricular mass index; M/V: mass to volume ratio; EDV: end left ventricular diastolic volume; ESV: end left ventricular systolic volume; SV: stroke volume; HR: heart rate; BMI: body mass index; NT-proBNP: N-terminal pro b-type natriuretic peptide; LVH: left ventricular hypertrophy; NA: not available; +: with hypertension; -: without hypertension.
