# Supplementary material for: H3K27ac acetylome signatures reveal the epigenomic reorganization in remodeled non-failing human hearts
Source: Clin Epigenetics. 2020 Jul 14;12:106. doi: 10.1186/s13148-020-00895-5 (PMC7362435; doi:10.1186/s13148-020-00895-5)
Supplement: Supplementary file 13 — Additional file 13. Supplementary Table 7. Overview of included samples in H3K27ac ChIP-seq, RNAPII ChIP-seq, and RNA-seq experiments. [file 13148_2020_895_MOESM13_ESM.docx]

**Supplementary Table 7.** An overview of included samples in H3K27ac ChIP-seq, RNAPII ChIP-seq, and RNA-seq experiments.

| Group | Sample ID | H3K27ac ChIP-seq | RNAPII ChIP-seq | RNA-seq* |
| --- | --- | --- | --- | --- |
| Controls | Control_1 | Yes | -- | -- |
|  | Control_2 | Yes | Yes | Yes |
|  | Control_3 | Yes | Yes | Yes |
|  | Control_4 | Yes | Yes | Yes |
|  | Control_5 | Yes | Yes | Yes |
| Patients | AS_1 | Yes | -- | Yes |
|  | AS_2 | Yes | Yes | Yes |
|  | AS_3 | Yes | Yes | Yes |
|  | AS_4 | Yes | Yes | Yes |
|  | AS_5 | Yes | -- | Yes |
|  | AS_6 | Yes | Yes | Yes |
|  | AS_7 | Yes | -- | Yes |
|  | AS_8 | Yes | -- | Yes |
|  | AS_9 | Yes | -- | Yes |
|  | AS_10 | Yes | -- | Yes |
|  | AS_11 | Yes | -- | Yes |
|  | AS_12 | Yes | Yes | Yes |
|  | AS_13 | Yes | -- | Yes |
|  | AS_14 | Yes | -- | Yes |
|  | AS_15 | Yes | -- | Yes |
|  | AS_16 | Yes | -- | Yes |
|  | AS_17 | Yes | -- | Yes |
|  | AS_18 | Yes | Yes | Yes |
|  | AS_19 | Yes | -- | -- |
|  | AS_20 | Yes | -- | -- |

*: Standard RNA-seq was performed in control samples. Due to the limited sample size and RNA amounts in patient samples, adjusted RNA-seq from CEL-seq was performed for the transcriptional comparison analysis between patients and controls.
